# Developmental trajectory of *Caenorhabditis elegans* nervous system governs its structural organization

**DOI:** 10.1371/journal.pcbi.1007602

**Published:** 2020-01-02

**Authors:** Anand Pathak, Nivedita Chatterjee, Sitabhra Sinha

**Affiliations:** 1 The Institute of Mathematical Sciences, CIT Campus, Taramani, Chennai, India; 2 Homi Bhabha National Institute, Anushaktinagar, Mumbai, India; 3 Vision Research Foundation, Sankara Nethralaya, Chennai, India; Ghent University, BELGIUM

## Abstract

A central problem of neuroscience involves uncovering the principles governing the organization of nervous systems which ensure robustness in brain development. The nematode *Caenorhabditis elegans* provides us with a model organism for studying this question. In this paper, we focus on the invariant connection structure and spatial arrangement of the neurons comprising the somatic neuronal network of this organism to understand the key developmental constraints underlying its design. We observe that neurons with certain shared characteristics—such as, neural process lengths, birth time cohort, lineage and bilateral symmetry—exhibit a preference for connecting to each other. Recognizing the existence of such homophily and their relative degree of importance in determining connection probability within neurons (for example, in synapses, symmetric pairing is the most dominant factor followed by birth time cohort, process length and lineage) helps in connecting specific neuronal attributes to the topological organization of the network. Further, the functional identities of neurons appear to dictate the temporal hierarchy of their appearance during the course of development. Providing crucial insights into principles that may be common across many organisms, our study shows how the trajectory in the developmental landscape constrains the structural organization of a nervous system.

## Author summary

Nervous systems of even the simplest of organisms involve a large array of neurons communicating with each other through a bewildering pattern of interconnections. What are the forces that govern the formation of this complex network? Using the somatic nervous system of the nematode Caenorhabditis elegans, we show how developmental constraints reflected by the symmetric pairing, birth times, process lengths and cell lineages of individual neurons are related to the observed spatial arrangement of neurons, as well as, the structural and functional organization of the network in the adult nematode. Our work suggests that focusing on the developmental trajectory, which complements approaches involving optimality considerations, may reveal the strategies underlying the development of a nervous system that may be robust across organisms.

## Introduction

The presence of an efficient machinery for responding immediately to changes in the environment with appropriate actions is essential for the survival of any organism. In almost all multicellular animals, this role is played by the nervous system comprising networks of neurons, specialized cells that rapidly exchange signals with a high degree of accuracy. It allows information about the environment obtained via sensory receptors to be processed and translated into output signals conveyed to effectors such as muscle cells. In even the simplest of such organisms, the structural description of the interconnections between neurons provided by the connectome presents an extremely complicated picture [1]. How the complex organization of the nervous system is generated in the course of development of an organism, occasionally referred to as the “brain wiring problem” [2], is one of the most challenging questions in biology [3, 4]. Only over the past few decades is the intricate interplay of different developmental phenomena, including cellular differentiation, migration, axon guidance and synapse formation, responsible for the formation of the network, being gradually revealed [5–9].

The free-living nematode *Caenorhabditis elegans*, the only organism whose entire connectome has been reconstructed so far [10, 11], is the natural choice for a system in which to look for principles governing the development of complexity in the nervous system [12]. The nervous system of the mature hermaphrodite individuals of the species comprises 302 neurons, which is about a third of the total complement of 959 somatic cells in the animal. Their lineage, spatial position and connections to each other appear to be almost invariant across individuals [10, 13]. The small number of cells constituting the worm has made it a relatively tractable system for understanding the genetic basis of metazoan development and behavior. This, however, belies the sophistication of the organism which exhibits almost all the important specialized tissue types that occur in larger, more complex animals, prompting it to be dubbed as a “microchip animal” [14]. The availability of its complete genome sequence [15] along with detailed information about the cell lineage [16, 17] means that, in principle, the developmental program can be understood as a consequence of genetically-encoded instructions and self-organized emergence arising from interactions between diverse molecules and cells [18].

The “wiring problem” for the *C. elegans* nervous system had been posed early on with Brenner essentially raising the following questions: how are the neurons spatially localized in their specific positions, how they connect to each other through synapses and gap junctions forming a network with a precisely delineated connection topology, and what governs the temporal sequence in which different neurons appear over the course of development [19]. Subsequent work has identified several mechanisms underlying the guidance of specific axons and formation of synapses between particular neurons [20–22]. However, the minutiae of the diverse molecular processes at work may be too overwhelming for us to arrive at a comprehensive understanding of how the complexity manifest in the nervous system of the worm arises. Indeed, it is not even clear that all the guidance cues that are involved in organizing the wiring are known [23]. An analogous situation had prevailed five decades earlier when *C. elegans* had been first pressed into service to understand how genetic mutations lead to changes in behavior of an organism. Brenner had responded to this challenge by analyzing the system at a level intermediate between genes and behavior [19]. Thus, the problem was decomposed into trying to understand (a) the means by which genes specify the nervous system (*how is it built?*) and (b) the way behavior is produced by the activity of the nervous system (*how does it work?*) [18, 19]. In a similar spirit, for a resolution of the “wiring problem”, we may need to view it at a level intermediate between the detailed molecular machinery involving diffusible factors, contact mediated interactions, growth cone guidance, etc., and the organization of the neuronal network in the mature worm. Specifically, in this paper, we have focused on uncovering a set of guiding principles that appear to govern the neuronal wiring and spatial localization of cell bodies, and which are implemented by the molecular mechanisms mentioned earlier (and thus genetically encoded). From the perspective of the three-level framework proposed by Marr [24, 25] for understanding the brain [4], viz., comprising (i) goals (and the logic of strategies for achieving them), (ii) algorithmic and (iii) implementation levels, such principles can be viewed as *strategies* for achieving specific network designs realized over the course of development [2].

For this purpose, we have used the analytical framework of graph theory, which has been successfully applied to understand various aspects of brain structure and function, in both healthy and pathological conditions [26–31]. For the specific case of the *C. elegans* nematode, application of such tools has revealed the existence of network motifs [32], hierarchical structure [33], community (or modular) organization [34] and a rich club of highly connected neurons [35]. Comparatively fewer studies have focused on the evolution of the network during development of the nematode nervous system that we consider here [36, 37]. We have integrated information about spatial location of cells, their lineage, time of appearance, neurite lengths and network connectivity to understand how its developmental history constrains the design of the somatic nervous system of *C. elegans*, specifically the 279 connected neurons which control all activity of the worm except the pharyngeal movements. Thus, our study complements existing work that has focused more on understanding the structural organization of the network using efficiency and optimality criteria such as minimization of the wiring cost, delineated by the physical distance between neurons [38–44].

The key questions related to development that we address here involve the spatial location of the cell bodies (*why is the neuron where it is, relative to other neurons?*), the temporal sequence in which the cells appear (*why is it that certain neurons are born much earlier than others?*) and the topological arrangement of their inter-connections (*why does a neuron have the links it does?*). As reported in detail below, we find that these questions are related to the existence of general principles that can be expressed in terms of different types of homophily, the tendency of entities sharing a certain feature to preferentially connect to each other. We discern four different types of homophily, involving respectively, process or neurite length of neurons, the time of their appearance, their lineage history and bilateral symmetry. We also estimate the relative contributions of each of these four factors (which we show are linearly independent of each other) in determining the connectivity. Although it had been reported earlier that the probability of connection between two neurons decreases with increasing difference in their birth times [36], we show this result to be much more nuanced in that birth cohort homophily is predominant only for connections between neurons whose cell bodies are physically proximate. Our results also help reveal that the ganglia, anatomically distinct bundles into which the neurons are clustered in the nematode, are formed of several groups (or families) of cells, neurons within each group being closely related. Furthermore, as lineage relation between neurons is an important factor that influences the structure of the neuronal network, we have presented a stochastic generative model for the lineage tree of cells. By invoking a simple asymmetric branching process, such a model captures several features of the empirically observed lineage tree.

At a higher level of network organization, we show that neurons which play a vital role in coordinating activity spanning large distances across the network by connecting together distinct neuronal communities (or modules) also appear quite early in the sequence of development. This observation (along with others, such as linking the functional type of neurons, viz., sensory, motor and inter, to their time of appearance) helps link the situation of a specific cell in the temporal hierarchy to which all neurons belong, with its function. We also provide an analysis of the inter-relation between functional, structural and developmental aspects, focusing on neurons identified to belong to different functional circuits, such as those associated with mechanosensation [45–47], chemosensation [48], etc. This provides us with a more nuanced understanding of the relation between the time of appearance of a neuron and the number of its connections. Our results suggest that developmental history plays a critical role in regulating the connectivity and spatial localization of neurons in the *C. elegans* nervous system. In other words, development itself provides key constraints on the system design. In addition, the tools we employ here for revealing patterns hidden in the lineage and connectivity information, including novel visual representations of developmental history, such as chrono-dendrograms, provide insights into principles governing the wiring of nervous systems that may be common across several organisms.

## Results

### Homophily based on multiple cellular properties governs neuronal inter-connectivity

Direct contact between neurons whose cell bodies are located relatively far apart, through synapses or gap junctions located on their extended processes, plays a crucial role in reducing communication delay of signals across the entire nervous system [49]. This is particularly relevant for *C. elegans* where the majority of synapses occur *en passant* (forming at axonal swellings) between parallel nerve process shafts that can remain close to each other over long distances [13]. Therefore, in order to understand the principles governing the wiring organization of the nematode nervous system, it is appropriate to first focus on understanding how the connectivity of neurons is influenced by the length of their neurites.

**Fig 1:**
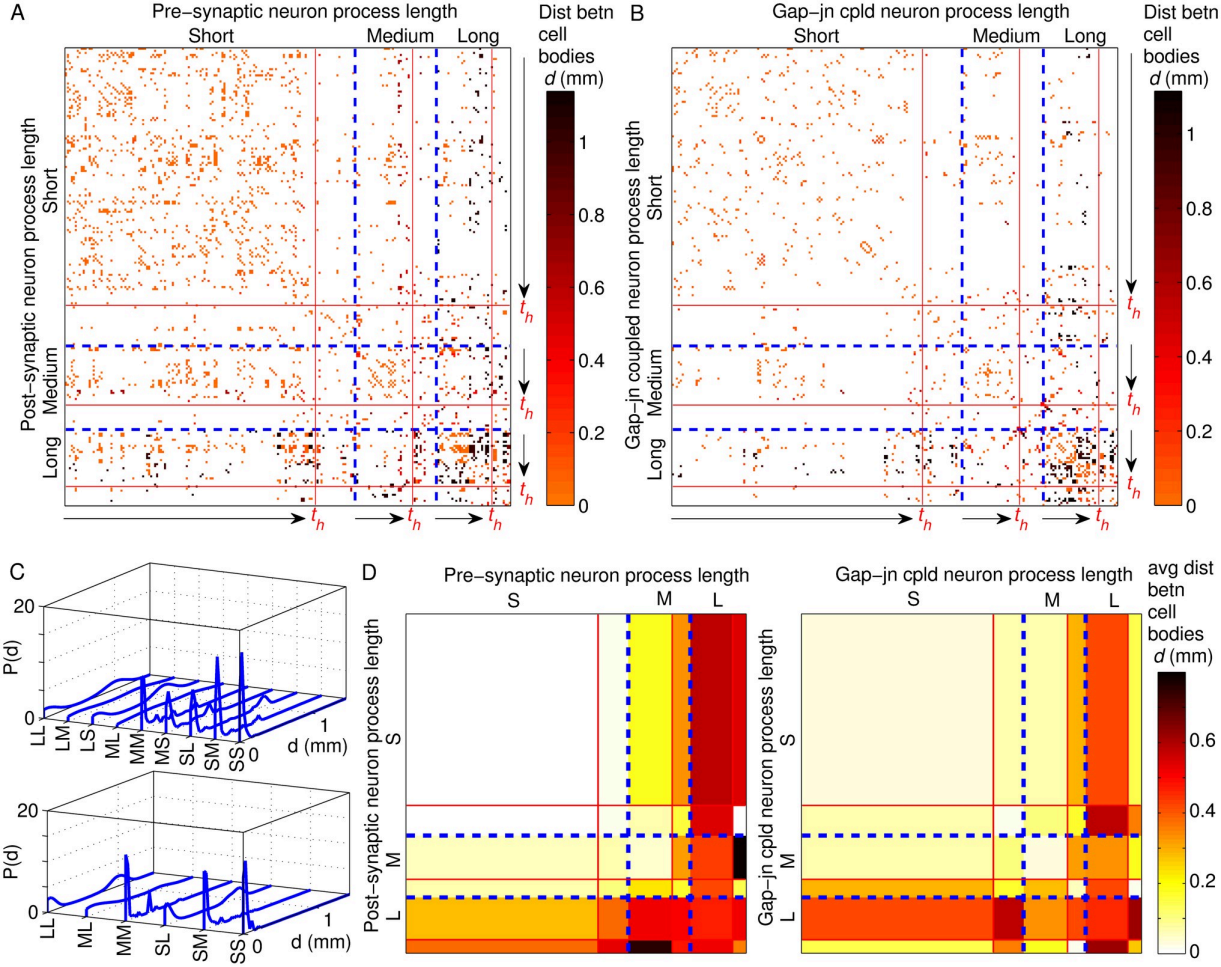
Birth time cohort membership and neurite lengths of neurons govern their connectivity. (A-B) Matrices representing synaptic (A) and gap-junctional (B) connections that exist between neurons, grouped into three classes [indicated by blue broken lines] according to their process lengths *ℓ* measured relative to the worm body length *L*, viz., short (*ℓ* ≤ *L*/3), medium (*L*/3 < *ℓ* ≤ 2*L*/3) and long (*ℓ* > 2*L*/3), and ordered within each class according to birth time. Increasing birth time is indicated by arrows, with red lines marked *t*_*h*_ (time of hatching) separating neurons which differentiate in the embryonic stage from those born later. Matrix entries correspond to the existence of a connection, with its color representing the distance (measured in mm) between cell bodies of the corresponding neurons (see legend). We observe that there is evidence of birth time assortative mixing, with neurons born early(later) having a higher probability of connecting with other early(late) born neurons, which is particularly marked in the case of neurons having short processes. The gap junction matrix shows a large number of entries adjacent to the diagonal which correspond to connections between paired neurons [see Fig 5(A)]. (C) Distribution of distances *d* between cell bodies of pairs of neurons distinguished in terms of their respective process lengths (S: short, M: medium, L: long), which are connected by synapses (top) and gap junctions (bottom). As synaptic connections are directed, there are nine possible combinations of pairs of the classes (S/M/L) to which the pre- and post-synaptic neurons belong (e.g., SL refers to a synapse from a neuron with a short process to a long process length neuron). On the other hand, as gap junctions are undirected, only six possible combinations need be considered. We note the bimodal distributions of *d* when at least one of the two neurons connected by synapse or gap junction has a long (or medium) process. (D) The mean distance 〈*d*〉 between cell bodies of neurons connected by synapses (left) and gap junctions (right) are grouped according to their process lengths (L/M/S) [indicated by blue broken lines] and further subdivided into those born early (i.e., embryonic stage) and those born late (i.e., L1, L2 or L3 stages) [separated by red lines]. Distances are expressed in mm (see legend for the color code). We note that pre-synaptic neurons with long processes tend to connect with post-synaptic neurons having short processes which are located far from them, corresponding to the higher peak in the bimodal distribution for LS in top panel of (C). Note that we have considered in this analysis the subset of 225 neurons for which information about process length is available.

It has also been observed that connected pairs of neurons very often differentiate close to each other in time [36]. This may suggest that preferential connectivity among neurons according to the time of their birth (i.e., *birth cohort homophily*) is a possible basis for guiding the network architecture. However, we need to explore the possibility that it could be a consequence of the restrictions on connections between neurons imposed by their respective process lengths. For instance, a large majority of the neurons that are born early, i.e., prior to hatching, are localized in the head region and have short processes extending to less than a third of the body length of the nematode. This could, in principle, be sufficient to explain the temporal closeness of connected neurons. We have accordingly investigated the joint dependence of the occurrence of connections (synapses and gap-junctions) between neurons on the lengths *ℓ* of their respective processes, as well as, their birth times *t*_*b*_ in Fig 1(A) and 1(B). The distance *d* between the cell bodies for each pair of connected neurons is also indicated, which makes apparent the restriction on connectivity imposed by the process lengths. This information adds a temporal dimension to our understanding of the organization of long-range connections (corresponding to high values of *d*) in the nematode nervous system. The neurons are grouped according to their process lengths *ℓ*. These are categorized as short (*ℓ* ≤ *L*/3), medium (*L*/3 < *ℓ* ≤ 2*L*/3) and long (*ℓ* > 2*L*/3) relative to the total body length of the worm *L*. Moreover, within each category, the neurons are arranged by their time of birth in increasing order.

#### Process length homophily

Even a perfunctory perusal of the two matrices in Fig 1(A) and 1(B) makes it apparent that the diagonal blocks in the two matrices have relatively higher density of points. This observation indicates that there is a preponderance of connections between neurons having similar process lengths. However, to establish that there is indeed *process length homophily* which would imply an explicit preference for neurons to connect to other neurons whose neurites extend to similar distances as them, we will have to compare the empirically observed number of such connected pairs with that expected to arise by chance given the degree (i.e., the total number of connections) of each neuron. For this, we cluster the cells into three *communities* or *modules* which are characterized by all their members having short, medium or long processes, respectively. This allows us to calculate the *modularity* *Q*, a measure of the extent to which like prefers connecting to like in a network [50, 51] (see Methods for details). A positive value of *Q* for a particular module would suggest that there is a bias for its members to preferentially connect to each other, while *Q* ∼ 0 indicates the absence of any evidence for homophily. For the entire synaptic network, we measure *Q* to be 0.125, while for the network of neurons connected by gap junctions, it is 0.18. We find these empirical *Q* values to be significantly higher than the corresponding values, viz., −0.003 ± 0.008 and −0.003 ± 0.013, calculated for ensembles of randomized surrogates for the synaptic and gap-junctional networks, respectively, obtained by randomly permuting the process length category membership of each neuron (see Methods). This suggests that neurons having similar process lengths do indeed have many more of their connections with each other than would be expected simply on the basis of the number of synapses and gap junctions possessed by each of them. We have additionally considered another surrogate ensemble obtained by randomly permuting the connections of the network keeping the degree of each neuron unchanged, subject to constraints imposed by the neuronal process lengths given the spatial positions of the cell bodies (see Methods). With respect to this ensemble also the empirical *Q* values are seen to be significantly higher (see Supporting Information, S1 Table). Thus, although the empirical values of the modularity appear to be small, they cannot be attributed simply to noise and suggests the existence of specific mechanisms that make connections between two neurons, both of which have short (or long) processes, more likely. Moreover, individually considering the three communities comprising neurons having short, medium and long processes, respectively, yields class-specific *Q* values (see Methods) which are also significantly higher than the *Q* values obtained from the corresponding randomized surrogates. In contrast, the class-specific *Q* values obtained for connections between neurons belonging to different categories of process lengths are either lower or about the same as the *Q* values obtained from the corresponding randomized surrogates, as indicated by the respective *z*-scores (see Supporting Information, S1 Table). This further underlines the existence of a significant bias for connections to occur between neurons having similar process lengths.

#### Birth cohort homophily

We observe a relatively high density of points in Fig 1(A) and 1(B) in the blocks corresponding to connections between cells having short processes (i.e., SS) that are *born at the same epoch*, i.e., either pre- or post-hatching. This is also seen for connections between neurons having medium length processes (MM), as well as, those between neurons having short and other neurons having medium process lengths (MS or SM). This suggests that, apart from process length, the time of birth of the cells also determine neuronal inter-connectivity. Indeed, earlier studies [36] have shown that most of the neurons that are connected to each other happen to be born close in time, with the probability of connection between contemporaneous neurons being much more than what is expected by chance. However, we find that the actual temporal separation between the time of birth of different neurons does not have any significant correlation (viz., *p* ≫ 0.05) with the probability of there being a connection between them, either synaptic or gap-junctional. This apparent contradiction is resolved on noting the following. While, within the group of neurons born in the embryonic stage and those born post-embryonic there may be a great diversity in terms of birth times (thereby significantly weakening any correlation with connection probability), these differences are minor when viewed from the perspective of membership in the cohorts of those born pre- and post-hatching, respectively. As reported earlier [36], these correspond to two distinct, temporally separated bursts of neuronal differentiation, which provides a natural demarcation of the neurons into early and late-born categories.

We observe that neurons prefer to connect to other members of their cohort (viz., early or later-born). This is indicative of *birth cohort homophily*, which is quantitatively established by segregating the neurons born pre- and post-hatching into two communities and then calculating *Q* values. As shown in Supporting Information, S2 Table, the *Q* for synapses is 0.09 and that for gap junctions is 0.07, which are significantly higher than the corresponding values obtained from the randomized surrogate ensemble (obtained by keeping the degree sequence unchanged, subject to constraints imposed by process lengths given the spatial position of cell bodies), viz., 0.02 ± 0.005 for synaptic and 0.03 ± 0.01, respectively.

We note that this homophily is restricted to neurons whose cell bodies are located in close physical proximity (see Supporting Information, S1 Fig). By comparing with randomized surrogates, we observe that connections between neurons are not significantly enhanced if they are born in the same epoch except for the case when the distance *d* between their cell bodies is short (*d* < *L*/3).

#### Process lengths affect the spatial arrangement of neurons

So far, in our consideration of how connections between neurons is affected by their process lengths, we have not considered the information concerning the spatial position of the cell bodies of the connected neurons. Consideration of this information is important if we want to understand how activity of spatially distant parts of the organism are coordinated through long-range connections that allow signals to be rapidly transmitted across relatively large physical distances. Fig 1(C) and 1(D) show how the distance *d* between cell bodies of connected pairs of neurons are distributed differently according to their respective process lengths.

The top panel in Fig 1(C) corresponds to the probability distribution function of distance between cell bodies *d* for neurons connected by synapses, while the bottom panel considers gap junctions. When both the pre- and post-synaptic neurons have short processes (indicated by SS in the figure), it is expected that the cell bodies will be located close to each other. This is indeed what is observed, with a prominent peak of *P*(*d*) occurring at extremely low values of *d*. On the other hand, when at least one of the neurons has a long or medium length process, we observe that the distributions for neurons connected through synapse are much more extended towards higher values. For SL, LS and LL connections, we in fact observed a distinct bimodal character in the corresponding distribution of *d*. This can be linked to the observation that neurons having short as well as long processes tend to predominantly have their cell bodies located at the head or in the tail of the worm. In contrast, neurons whose processes are intermediate in length have cell bodies distributed relatively more homogeneously across the body of the organism (see Supporting Information, S2 Fig). This can be quantified by measuring the extent to which the cell bodies themselves are distributed along the longitudinal axis of the nematode body in a bimodal manner using the Bimodality Coefficient (*BC*) metric [52] (see Methods). A distribution is said to be prominently bimodal if its *BC* ≫ BC* (= 5/9), the value of the metric for an uniform distribution. We find that while the spatial positions of the cell bodies of neurons having short, as well as, long process are distributed in a bimodal manner (*BC*_*S*_ = 0.93 and *BC*_*L*_ = 0.83, respectively), that of neurons with intermediate length process (*BC*_*M*_ = 0.67) are relatively more uniformly distributed. Accordingly, we observe that synaptically connected pairs, in which at least one neuron has process of medium length, exhibit distributions of *d* where bimodality is either muted (as in SM, MM and MS) or absent (ML and LM), even though all of these distributions span a much larger range of *d* than SS. This indicates that process length is an important determinative factor for the occurrence of long-range connections in the nematode nervous system.

When we consider the distribution of distances between cell bodies of neurons connected by gap junctions [lower panel of Fig 1(C)], we observe that connections are more likely to occur between spatially adjacent cell bodies. This is manifest in the distributions of *d* being much less extended than those seen in the case of synapses, with the exception of SL and LL which exhibit bimodality. The distinction between the situations seen in the upper and lower panels may arise from the fact that while synapses between two neurons can in principle be located anywhere on their processes, gap junctions predominantly occur close to the cell body of at least one of the participating neurons.

The detailed nature of the information about the number of neuronal pairs with given process lengths whose cell bodies are placed a specific distance *d* apart that is provided by the distributions shown in Fig 1(C) tends to obscure certain gross features. The latter can impart important insights into how process length facilitates connections between spatially distal neurons. Therefore, in Fig 1(D) we display the average physical distance between cell bodies of *connected* neurons which are distinguished in terms of their process lengths (short/medium/long), and further subdivided into those appearing in the embryonic stage, i.e., prior to hatching (referred to as early), and those which appear at the post-embryonic stage (referred to as late). For synaptic connections (shown at left), the average *d* for neurons with long processes (pre-synaptic) connected to neurons having short processes (post-synaptic) is the highest (〈*d*_*LS*_〉 = 0.57 mm) of all the categories considered, higher even than that when both neurons in a connected pair have long processes (〈*d*_*LL*_〉 = 0.50 mm). Intriguingly, both of these values are larger than the average distance between cell bodies for connected neurons when the pre-synaptic neurons have short processes while the post-synaptic ones have long processes, viz, (〈*d*_*SL*_〉 = 0.33 mm). This is consistent with the two peaks of the bimodal distribution of *d* corresponding to these connections differing substantially in amplitude—the peak at lower *d* being higher for SL, while the one at higher *d* being larger for LS. To a lesser extent, a similar asymmetry is seen for the average distance between connected cell bodies when one has short process while the process of the other is of medium length (viz., 〈*d*_*MS*_〉 = 0.22 mm as compared to 〈*d*_*SM*_〉 = 0.09 mm).

We can compare these values with the average distance between cell bodies of *all* neurons, whether connected or not. For instance, the mean separation *D* between cell bodies of all neurons with long process lengths is 〈*D*〉_*L*,*L*_ = 0.55 mm which is almost the same as the average distance between every pair of neurons in which one has a short process and the other has a long one (〈*D*〉_*L*,*S*_ = 0.54 mm). To ensure that the difference between 〈*d*_*XY*_〉 and 〈*D*〉_*X*,*Y*_ (where *X*, *Y* ∈ {*S*, *L*, *M*}) is statistically significant, we show that it is extremely unlikely that the observed values of *d* will arise by chance if random surrogates are constructed having the same number of connected neurons as is observed empirically (by sampling the set of all neuronal pairs without replacement). For instance, the *z*-score (see Methods) for the distance between cell bodies of pre-synaptic neurons with long processes connected to post-synaptic neurons with short processes is *z*_*LS*_ = 1. By contrast, considering the reverse, i.e., synapses from neurons with short processes to those having long processes, we obtain *z*_*SL*_ = −7.8. Thus, neurons with long processes appear to form a synapse with neurons having short processes whose cell bodies are located far away from their own much more often than that expected by chance given the spatial positions of the cell bodies. On the other hand, neurons with short processes prefer to connect to neurons with long processes whose cell bodies are much closer to their own. Indeed, excepting the class of LS and ML synaptically connected neuron pairs (i.e., pre-synaptic neurons with long process with post-synaptic neuron with short process and pre-synaptic neurons with medium process with post-synaptic neuron with long process), all other connected neural pair classes, distinguished in terms of the process lengths of the two neurons, have negative values for *z*-score (see Supporting Information, S3 Table, and S3 and S4 Figs). The results indicate that the process length of the pre-synaptic neuron is a dominant influence deciding the average distance between cell bodies connected by synapses. It is also consistent with the possibility that a high proportion of synaptic contacts are occurring close to the cell body of the post-synaptic neuron (which is closer to the classical concept of the pre-synaptic axon connecting to a dendrite close to cell body of the post-synaptic neuron and not just making a synaptic contact anywhere on the process). Such asymmetry between LS and SL may also have the advantage of functional efficiency in that the resulting connection architecture allows signals to rapidly travel large distances across the nematode body through long processes—thereby spreading globally using L to S connections—and then being disseminated locally using neurons with short processes.

If we now consider the case of neurons connected by gap-junctions [Fig 1 (D, right)], we note that the average value of *d* is highest for the case of cells with long processes connecting to each other. In particular, unlike the situation seen above for synaptically connected neurons, 〈*d*_*LS*_〉(= 0.44 mm) is lower than 〈*d*_*LL*_〉(= 0.5 mm). The *z*-score for the distance between cell bodies of neuron pairs whose members belong to any of the classes S, M and L are seen to be strongly negative, ranging between *z*_*LL*_ = −1.6 and *z*_*SS*_ = −12.7. The high statistical significance of 〈*d*〉 when compared against the average separation between neurons 〈*D*〉 suggests that gap junctions occur between neurons whose cell bodies lie close to each other far more often than expected by chance (given their positions). This is consistent with the belief that gap junctions predominantly act to coordinate activity locally between neurons [53]. We also note in passing another feature of gap junctional connections between neurons which is manifest in Fig 1(B) as a large number of entries in the adjacency matrix immediately neighboring the diagonal. These correspond to a very high proportion of connections between bilaterally symmetric pair of neurons, e.g., AVAL and AVAR, that is discussed later (see bilateral symmetric pairing homophily). These connections may have the possible functional goal of coordinating response of the nematode nervous system to sensory inputs between the left and right sides of the body [54].

The process length homophily between neurons that we demonstrated above can be attributed to multiple possible factors. For instance, the preference of neurons having long process for connecting to other neurons with long processes could be an outcome of the geometry resulting from parallel fibers extending over relatively large distances, which have a proportionately higher probability of forming *en passant* synapses with each other. On the other hand, the preference of neurons having short processes to connect to each other could be tied to the fact that many of their cell bodies are located in close physical proximity. This suggests an important role for the physical distance *d* between cell bodies in deciding connectivity between neurons. When we look at the correlations between *d* and the probability that the cells are connected, we do not find any statistically significant correlation for either synapse or gap junctions. Focusing only on neuron pairs whose cell bodies are located close to each other (i.e., *d* ≤ *L*/3 where *L* is the total body length of the worm), however, we observe a very strong correlation of −0.92 (*p* = 0.003) between *d* and the probability of a synaptic connection between the two (for gap junctions, the correlation is −0.89 with *p* = 0.007). This high value indicates that synapse formation between neurons whose cell bodies are located near each other is indeed strongly dependent on the distance between them. Moreover, it cannot be explained in terms of simple physical limits imposed by the process lengths of neurons on the farthest distance allowed between cell bodies of connected neurons. This is because if we consider the correlation between *d* and probability of connection only between neurons having short processes, we obtain a value of −0.87 (*p* = 0.012) for synapses and −0.89 (*p* = 0.008) for gap junctions (see Supporting Information, S5 Fig).

A possible explanation for the weakening of the relation between connection probability and the physical distance separating the cell bodies when all neurons are considered could be because, even though neurons born in close physical proximity have a higher probability of getting connected, it is masked by the cells moving apart subsequently over the course of development. In the absence of information about the location of the cell bodies at the time synaptogenesis happens, we can probe this indirectly by considering how the probability of connection between two cells depends on how closely they are related in terms of lineage—as cells having common ancestry also tend to be born adjacent to each other.

#### Lineage homophily

**Fig 2:**
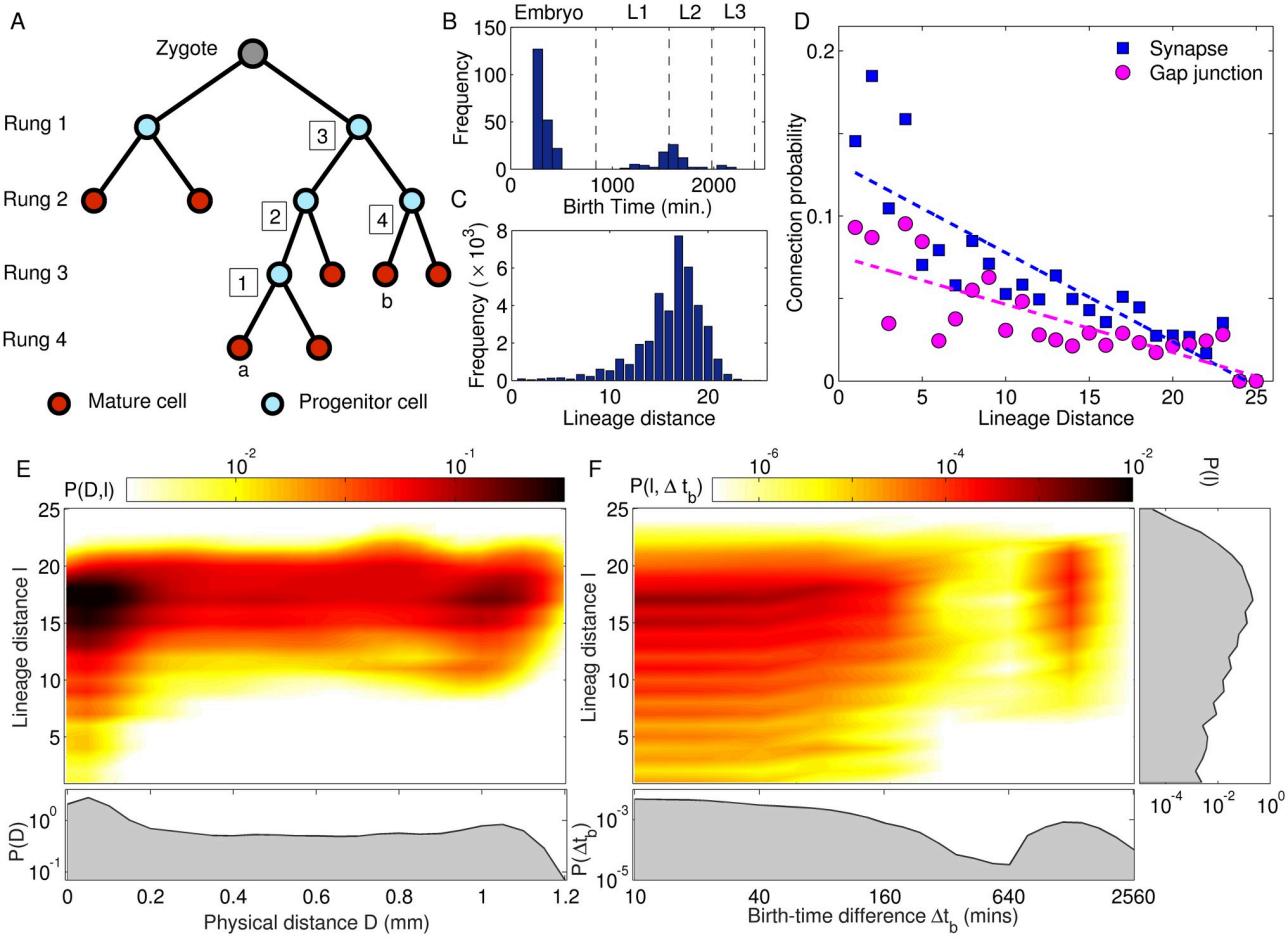
Lineage of neurons affects their synaptic connectivity and spatial localization. (A) Schematic diagram of a lineage tree of cells resulting from consecutive mitotic divisions of the zygote. The terminal nodes of the tree correspond to terminally differentiated mature cells (shown in red) while other nodes represent progenitors (shown in blue) that appear at different rounds of cell division. Cells born at each round of cell division are indicated by the corresponding rung of the tree they belong to, the numerical value for the rung (shown at the left) being the number of divisions starting from the zygote. The lineage distance *l* between a pair of mature cells is measured as the total number of cell divisions leading to each from their common progenitor. An example of lineage distance measurement is shown in the figure for the pair of cells *a* and *b* which are separated by four cell divisions (the distance of *a* from each of the intermediate dividing progenitors is indicated in the figure). (B-C) Frequency distributions of the birth time of different neurons (B, separated into the different developmental stages) and the lineage distances for each pair of neurons (C). (D) The probability of a pair of neurons to be connected through a synapse decreases with increasing lineage distance between them, as indicated by a statistically significant linear correlation between the two (*r* = −0.87, *p* < 10^−7^). For gap junctional connections, the correlation is marginally weaker (*r* = −0.79, *p* < 10^−5^). (E-F) Joint probability distributions of lineage distance *l* along with distance between cell bodies *D* (E) and birth time difference Δ*t*_*b*_ (F) between all pairs of neurons. The marginal distributions for the corresponding quantities are also shown. We notice that the distribution of physical distances in (E) exhibit a bimodal nature. However, cells which are closely related in terms of lineage (*l* < 5) also has a high probability of being physically located nearby (indicated by a prominent peak at the lower end of the distribution of *D*) which suggests that lineage influences spatial localization of cells. In panel (F), the distribution shows peaks at odd values of the lineage distance (particularly for low Δ*t*_*b*_) suggesting that neurons born close in time are located at the same rung on the lineage tree.

Cell lineage provides knowledge of the developmental trajectory in all metazoa, being defined by successive divisions starting from the zygote to the final differentiated cell. In most animals, the identity of any terminal node of the lineage tree, known as cell fate, is determined by intrinsic and extrinsic factors, as well as, interactions with neighboring cells. This introduces sufficient variability in the developmental path so as to make lineage relationships discernible only at the level of cell groups rather than individual cells [55]. However, some organisms such as nematodes exhibit an almost invariant pattern of somatic cell divisions that is identical across individuals, and in the case of *Caenorhabditis elegans*, is known in its entirety [16, 17]. Thus, the lineage tree of the organism provides us with a complete fate map at single-cell resolution [56]. The schematic representation of such a tree shown in Fig 2(A) depicts successive mitotic cell divisions starting from a zygote that, through intermediate progenitor cells, eventually differentiate into mature neuronal cells. Each successive cell division (beginning from the zygote) corresponds to different rungs in the tree used to label the resulting daughter cells. The difference between any two cells in terms of their lineage can thus be quantified by their lineage distance, i.e., their separation on the tree measured as the total number of cell divisions that leads to each of them from their last common progenitor.

Apart from the lineage tree, crucial information on the relationships between different cells that stem from their developmental history is provided by the knowledge of birth times of the individual mature neurons, i.e., the specific instant in developmental chronology of the nervous system at which each neuron differentiates. Fig 2(B) shows the distribution of birth times for all cells belonging to the somatic nervous system of *C. elegans*, indicating that development of the system occurs in two bursts clearly separated in time [36]. The ‘early burst’, during which the bulk, viz., 72%, of the neurons are born, occurs at the embryonic stage of development, while the more temporally extended ‘late burst’ spans across the L1 and L2 stages. This information, in conjunction with a simple generative model for reconstructing the lineage tree through successive cell divisions, can be used to explain the distribution of lineage distance shown in Fig 2(C). As at each node of the lineage tree a cell divides into at most two daughter cells, we can view it—at least in the first few rungs belonging to the early proliferative phase—as a balanced binary tree, with the number of cells that appear in each rung *R* increasing exponentially with *R* (upto *R* = 10 in *C. elegans*, see Supporting Information, S6 Fig). Within the AB sub-lineage of cells to which almost all the neurons belong, the maximum lineage distance that can occur between two cells which are placed in rungs *R*_1_ and *R*_2_, respectively, is given by *l*_*max*_(*R*_1_, *R*_2_) = (*R*_1_ − 1) + (*R*_2_ − 1) − 1. Thus, the distribution of lineage distances has an exponential profile upto *l* = 17. Beyond rung 10, the subsequent branching of the nodes in the binary tree reduce markedly as many of the divisions terminate in differentiated neurons (and occasionally programmed cell death) or lead to non-neuronal fates (so that their further divisions are not considered for the purpose of this study). This can be seen to result in the lineage distance distribution *decreasing* exponentially for *l* > 17, with a maximum lineage distance of 25. A more detailed theoretical model of the lineage relationships between neurons resulting from their developmental history can be constructed as an asymmetric stochastic branching process (see Methods). Here, beginning with a single node that corresponds to the zygote, at each iteration every node that appeared during the preceding iteration is considered in turn for giving rise to each of two possible branches with probabilities *P*1 and *P*2 (*P*1 ≥ *P*2) that result in further nodes. By considering the actual lineage tree, these asymmetric branching probabilities in the model were fixed as *P*1 = 1 and *P*2 = 0.85 until rung 9 and for later rungs they were set to *P*1 = 0.25 and *P*2 = 0.2. For these values of *P*1 and *P*2, the trees generated by the model exhibited properties that were statistically similar to the empirical lineage tree (see Supporting Information, S6 Fig).

Going back to the question we had posed earlier, viz., how does the lineage distance *l* between cells affect the probability that they are connected by synapses, we observe from Fig 2(D) that there is indeed a strong correlation of −0.87 (*p* < 10^−7^) between the two. For gap-junctions, we again observe a correlation between lineage distance and connection probability that is only marginally weaker, viz., −0.79 (*p* < 10^−5^), than that seen for synapses. This observation provides evidence of *lineage homophily* being one of the key principles governing connectivity of the nematode nervous system. The linear fits for the dependence of the connection probabilities on lineage distance (shown using broken lines) imply that synaptic connection probability has a slightly stronger dependence on the lineage distance compared to gap junctions, as indicated by the higher slope of the regression line for the former. These observations suggest that changes in the locations of cell bodies from that they occupied initially (i.e., at the time the corresponding neurons differentiated) which are brought about by the appearance of cells born later through subsequent cell-divisions, result in a weak correlation between connection probability and physical distance separating the cell bodies, as alluded to earlier.

#### Lineage relation between neurons constrains distance between their cell bodies

The connection between lineage distance *l* and physical distance *D* between cell bodies of neurons (whether connected or not), which has been mentioned earlier, is illustrated by the joint probability distribution *P*(*D*, *l*) shown in Fig 2(E). In particular, cells having short lineage distance, viz., *l* ≤ 5, tend to have their cell bodies located close to each other, as indicated by the function being peaked towards lower values of *D*. However, cells that are farther apart in terms of lineage can occur at different distances from each other, resulting in the overall bimodal form for the marginal distribution of *D*. A similar nuanced relation between lineage distance for two neurons and the difference of the times Δ*t*_*b*_ in which they are born is indicated by the joint probability distribution *P*(*l*, Δ*t*_*b*_) shown in Fig 2(F). We note that for small *l* (*l* ≤ 5), the distribution peaks at low values of Δ*t*_*b*_ indicating that closely related neurons tend to be born within a short time interval of each other. We also observe that the distribution of *l* between neurons that differentiate at around the same time (i.e., for low Δ*t*_*b*_) tends to alternate between peaks and troughs for odd and even values, respectively. This is easy to explain if neurons that are contemporaneous occur at the same rung (as, by definition, neurons at the same rung will have odd values of lineage distance between themselves).

#### The different ganglia comprise clusters of closely related neurons

The compelling association between lineage and physical proximity of neurons alluded to above is manifest in the spatial organization of the cell body locations. It is particularly conspicuous in the clustering of neurons into anatomically distinct bundles that are referred to as ganglia. These structures, characteristic of nematode nervous systems, contain only cell bodies of the neurons with their axonal and dendritic processes located outside of the bundles [57]. The somatic nervous system comprises nine such spatially localized clusters, viz., anterior, dorsal, lateral, ventral, retrovesicular, posterolateral, preanal, dorsorectal and lumbar ganglia, with the remainder belonging to the ventral cord. Comparison of the distributions of intra-ganglionic lineage distances (i.e., between pairs of neurons located in the same ganglion) with that of inter-ganglionic lineage distances (i.e., between neurons in different ganglia) provides an insight into how these bundles can be interpreted from a developmental perspective.

**Fig 3:**
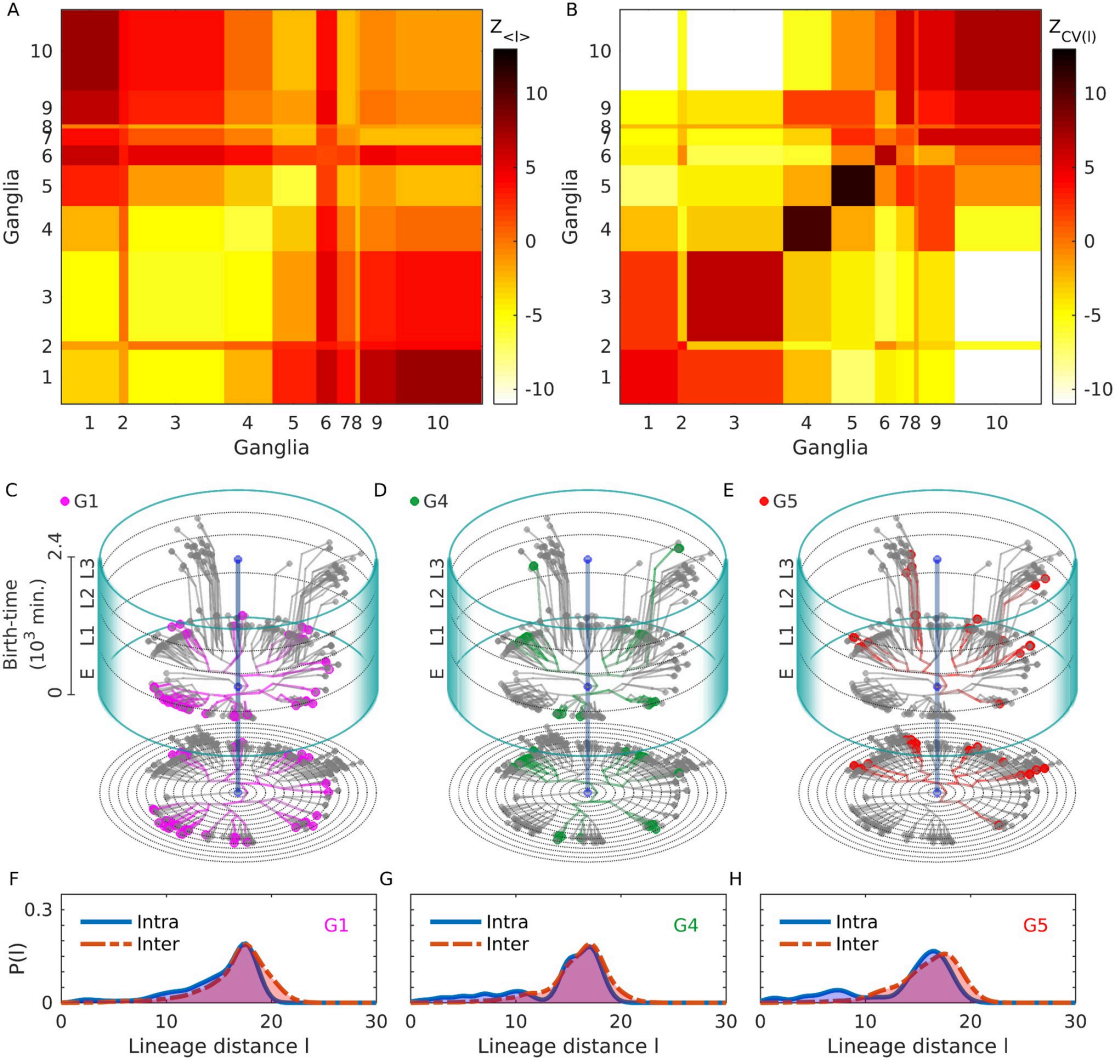
Lineage distance reveals developmental patterns of ganglia. (A-B) Statistically significant features of the distribution of intra and inter-ganglionic lineage distances, quantified by deviations of the mean 〈*l*〉 (A) and coefficient of variation *CV* (B), from a surrogate ensemble of randomized lineage trees of neurons in the C. elegans somatic nervous system. These deviations (measured by *z*-score) show that the mean intra-ganglionic lineage distances (represented by diagonal blocks of the matrix) are significantly lower than that of the inter-ganglionic lineage distances (off-diagonal blocks), with the exception of G6 and G9. By contrast, CV for the intra-ganglionic lineage distances are significantly higher than that of the inter-ganglionic lineage distances. (C-E) Developmental chrono-dendrograms for three representative ganglia (viz., G1, G4 and G5) show that each comprises multiple localized clusters of neurons occurring at different locations on the developmental lineage tree, explaining the statistically significant deviations of the mean and CV for intra-ganglionic lineage distances. Colored nodes represent neurons belonging to the specified ganglion while gray nodes show the other neurons. Branching lines trace all cell divisions starting from the single cell zygote (located at the origin) and terminating at each differentiated neuron. The time and rung of each cell division are indicated by its position along the vertical and radial axis respectively. The entire time period is divided into four stages, viz., Embryo (indicated as E), L1, L2 and L3. A planar projection at the base of each cylinder shows the rung (concentric circles) of each progenitor cell and differentiated neuron. (F-H) The probability distribution functions for the intra-ganglionic lineage distances show bimodality (unlike that of the inter-ganglionic distances), which is consistent with the segregation of a ganglion into multiple clusters along the chrono-dendrogram. The different ganglia are indicated by symbols G1-G9 (1: Anterior, 2: Dorsal, 3: Lateral, 4: Ventral, 5: Retrovesicular, 6: Posterolateral, 7: Preanal, 8: Dorsorectal and 9: Lumbar) and the Ventral cord as G10.

We first note that the mean of the lineage distances 〈*l*〉 within a given ganglia are typically much smaller than those between different ganglia. Moreover, as seen from Fig 3(A), the mean of the intra-ganglionic lineage distances for most ganglia are significantly small, which we determine by comparing with values of 〈*l*〉 obtained from ensembles of 10^3^ surrogate lineage trees where the identity of each of the leaf nodes (i.e., the differentiated neurons) has been randomly permuted. This randomization decouples the ganglionic membership of the neurons from their position on the lineage tree while keeping the lineage distances between cells invariant, consistent with our null hypothesis that the ganglion to which a neuron belongs is independent of its developmental history. The observed mean intra-ganglionic lineage distances deviate markedly from those obtained from the surrogate trees (as measured by *z*-score, see Methods), indicating that neurons in a ganglion are much more closely related to each other than expected by chance. We note however that, the Posterolateral (G6) and Lumbar (G9) ganglia are exceptions to the rule, in that they do not exhibit a significantly negative *z*-score like the other ganglia.

However, when we consider the coefficient of variation (*CV*), a relative measure of the dispersion in the lineage distances within a ganglion or between two ganglia, we note that this is almost always greater for intra-ganglionic, compared to the inter-ganglionic, lineage distances [Fig 3(B)]. We can again establish the statistical significance by measuring the same quantities for the ensemble of surrogate lineage trees mentioned above and quantifying the difference between the actual tree and the randomized ensemble using *z*-scores. The large values of *z* for CV in most of the diagonal blocks (corresponding to intra-ganglion dispersion) shown in Fig 3(B), suggests that the relatedness between neurons in a ganglion shows a much larger variability than expected by chance.

The apparent contradiction between the results mentioned above, viz., that a majority of the neurons in a ganglion have a shared lineage while, at the same time, exhibit a high degree of diversity in their lineage relations, is easily resolved on inspecting the chrono-dendrograms that visually represent the complete developmental trajectory for each of the ganglia [shown in Fig 3(C)–3(E), for the anterior, ventral and retrovesicular ganglia; see Supporting Information, S7–S9 Figs for the others]. While the lineage tree shown in each of these figures is, of course, identical, the neurons that belong to a particular ganglion are distinguished (by color) in the corresponding chrono-dendrogram, allowing us to note at a glance how all the members of the given ganglion relate to each other. We note that the differentiated neurons that constitute a ganglion are typically organized into multiple clusters, each of which are highly localized on the lineage tree. In other words, a ganglion comprises several ‘families’ of neurons emanating from different branches of the tree, with each family composed of closely related cells sharing a last common ancestor separated from them by only a few cell divisions.

The grouping of the cells belonging to a particular ganglion into distinct clusters, which are widely separated on the lineage tree, is reflected in the bimodal nature of the distribution of intra-ganglionic lineage distances [Fig 3(F)–3(H)]. In contrast to the unimodal distribution seen for inter-ganglionic lineage distances, the neurons within a ganglion could either have (i) extremely low distances to cells which belong to their own ‘family’ or (ii) large distances to cells belonging to the other ‘families’ that constitute the ganglion. These manifest, respectively, as a smaller peak at lower values and a larger peak at higher values of *l* seen in Fig 3(F)–3(H). The bimodality gives rise to a large dispersion and hence a value for the CV of lineage distances that is higher than expected. Note that the peak at higher *l* for this distribution almost coincides with the peak of the inter-ganglionic *l* distribution, which is expected as the latter is dominated by cells that are not closely related. Thus, the presence of the second peak at lower values of *l* in the intra-ganglionic distribution reduces the mean lineage distance for cells within a ganglion, compared to that for cells belonging to different ganglia. Conversely, the absence of multiple peaks in the inter-ganglionic distribution provides for a smaller value of the CV compared to the case for the intra-ganglionic distribution. Thus, these results explain the apparently contradictory coexistence of low mean value and high CV for lineage distances of neurons within a ganglion, which is related to the localization of the developmental trajectories of cells belonging to it into distinct groups visible in the lineage tree. This clearly demonstrates that the spatial segregation of neurons into ganglia is shaped by the relations between the constituent cells which arise from their shared developmental history.

#### Birth time and lineage relation together constrain the physical distance between cell bodies of connected neurons

**Fig 4:**
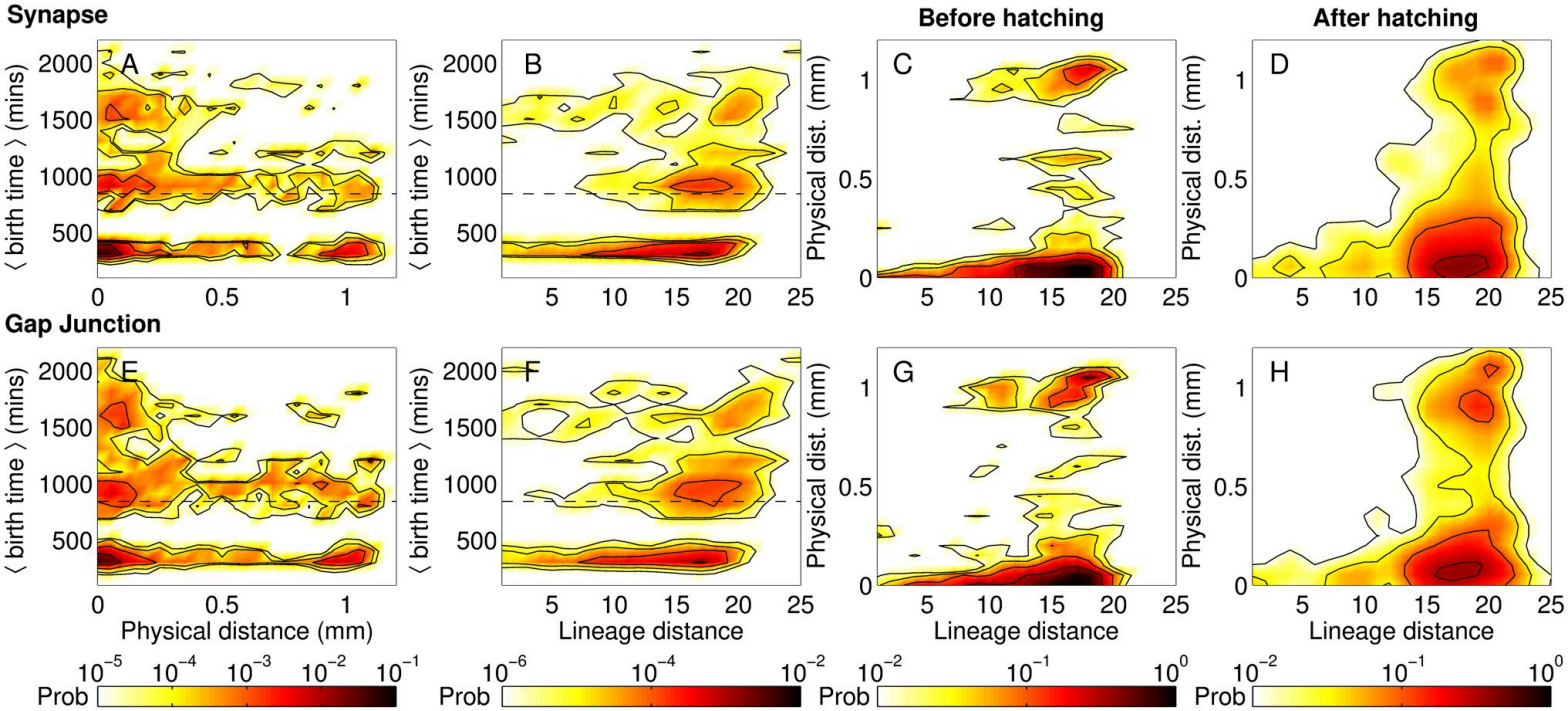
Birth times and lineage distances constrain connections between neurons whose cell bodies are spatially distant from each other. (A-B) The mean birth time of synaptically connected pairs of neurons exhibit a trimodal distribution, with connections clustering into three temporal groups corresponding to those (i) between neurons that are both born early, i.e., in the embryonic stage, (ii) between one born early and the other born late (i.e., in the post-embryonic stage), and (iii) between neurons that are both born late. The hatching time *h*_*t*_ separating the embryonic from other developmental stages is indicated by the broken line. We note from panel (A) that when both neurons are born late (corresponding to the uppermost cluster of connections), synaptic connections are more likely to occur between neurons whose cell bodies are located close to each other. (C-D) Synaptic connections between neurons that are closely related to each other in terms of lineage (*l* < 10) occur almost always when their cell bodies are in proximity, regardless of the time of birth of the neurons. We note that this restriction is more pronounced than observed in Fig 2(E), where *P*(*D*, *l*) shows a prominent peak at the lower end of *D* for small *l* suggesting that most closely related neurons (whether connected or not) typically have short distances between their cell bodies. (E-H) Neurons connected by gap junctions show patterns similar to those seen in the case of synaptic connections.

Having considered the distribution of physical distance, lineage distance and birth-time differences between all neuronal pairs in the somatic nervous system, we now focus on the subset of connected pairs to see how the above factors may constrain the probability that a neuron has a direct interaction with another. Fig 4 shows the inter-relations between similarity of ancestry, spatial separation and birth times for each pair of neurons that are linked either by synapses (top row) or gap junctions (bottom row). The clustering of mean birth times of the connected pairs into three distinct groups (seen in panels A-B and E-F) is a consequence of the two bursts of neuronal differentiation widely separated in time [seen in Fig 2(B)]. Thus, the lower and upper clusters correspond to connected neurons both of which appear in the course of the same developmental burst (early and late, respectively), while connections between neurons that arose during different bursts populate the intermediate cluster.

In Fig 2(F) we had already seen that closely related neurons tend to have similar birth times. This helps explain why, as seen in Fig 4(B), whenever synaptically connected neurons have short lineage distance to each other, they also happen to belong to the same developmental burst epoch. However, apart from the relative differences in the birth times, the actual time of differentiation also determines the occurrence of a synapse between neurons. Indeed, it is known from Ref. [36] that about 68% of long-range synaptic connections occur between neurons both of which are born in the early burst of neuronal differentiation. This is complemented by Fig 4(A) which shows that synapses between neurons, whose cell bodies are separated by large distances, mostly occur when at least one of the neurons was born early. Conversely, when both neurons are born in the late burst, such long-range links become extremely unlikely. Indeed, the distribution of distances between cell bodies of connected neurons (see Supporting Information, S10 Fig, that compares the empirical data with degree-preserved randomized networks where the connections are made according to constraints imposed by the length of processes of each neuron) show that long-range connections in the nematode typically do not occur significantly more often than that expected by chance, given the process lengths of the neurons. Thus, specific mechanisms for explaining the occurrence of such connections maybe unnecessary given that *en passant* synaptic contacts form between neighboring parallel neuronal processes. In contrast, short range connections are much more numerous than that seen in the random surrogate networks. This suggests that active processes may be driving synaptogenesis [21, 22] between neurons lying in close proximity, for example, chemoattractant diffusion [6, 8, 58]. Furthermore, the exceptional feature of early pre-synaptic neurons having long-range connections to late post-synaptic neurons much more often than is expected by chance could suggest a possible role of fasciculation in this process [9]. For instance, late-born neurons could be following the extended processes of earlier neurons to connect to cell bodies placed far away.

In Fig 4(C) and 4(D) we compare explicitly the pre- and post-hatching scenarios in order to see whether early and late-born neurons differ in terms of how the synaptic connections between them are influenced by the lineage and/or physical distances between them. We note that for both groups of cells, closely related neurons that are connected by synapse also happen to occur at spatially proximate locations. This is consistent with Fig 2(E) where the peak in the joint probability distribution of all neuronal pairs with lineage distance *l* and physical distance *D* is observed to occur at low *D* when *l* is small. Qualitatively similar results are observed when we consider neuronal pairs connected by gap junctions [see panels E-H of Fig 4].

The results reported above provide remarkable evidence for the role that developmental attributes (viz., lineage distances and birth-times of neurons) play in shaping the spatial organization of cell bodies and the topological structure of the connections in the somatic nervous system of the worm. However, the process length homophily described earlier appears to be independent and cannot be explained as a consequence of lineage homophily. The chrono-dendrograms (see Supporting Information, S11 Fig) showing the positions of neurons with short, medium and long processes, respectively, on the lineage tree indicate that neurons having a particular process length do not cluster together. This suggests that neurons with extremely similar lineage may have very different process lengths (and vice versa), so that the observed bias in the connection probability between neurons having processes of similar length cannot simply be attributed to a common lineage.

#### Bilateral symmetric pairing homophily

The major fraction (≈ 66%) of neurons belonging to the somatic nervous system of *C. elegans* occur in pairs. These are located along the left and right sides of the body of the nematode in a bilaterally symmetric fashion. While there are instances of bilaterally symmetric neurons exhibiting functional lateralization (e.g., ASEL/R, see Ref. [59]), the vast majority of the left/right members of such pairs remain in the symmetrical “ground state”, i.e., they are indistinguishable functionally, as well as, in terms of anatomical features and gene expression [60]. In particular, whenever one member of a bilaterally symmetric pair occurs in any of the known functional circuits obtained through behavioral assays [61–63] (discussed later), the other also appears in it without exception. While it is known that this symmetric nature is manifested in the spatial arrangement (e.g., location of the cell bodies) and connection structure of paired neurons, here we ask whether bilaterally symmetric neurons share a similar network neighborhood, i.e., whether there is a high degree of overlap between the neurons that each of them connect to, or indeed whether they have a significantly higher probability of being connected to each other. The latter assumes importance in view of the fact that it is the direct contact between the paired cells AWCL/R that trigger asymmetrical gene expression resulting in differential expression of olfactory-type G-protein coupled receptors in the neurons [64].

**Fig 5:**
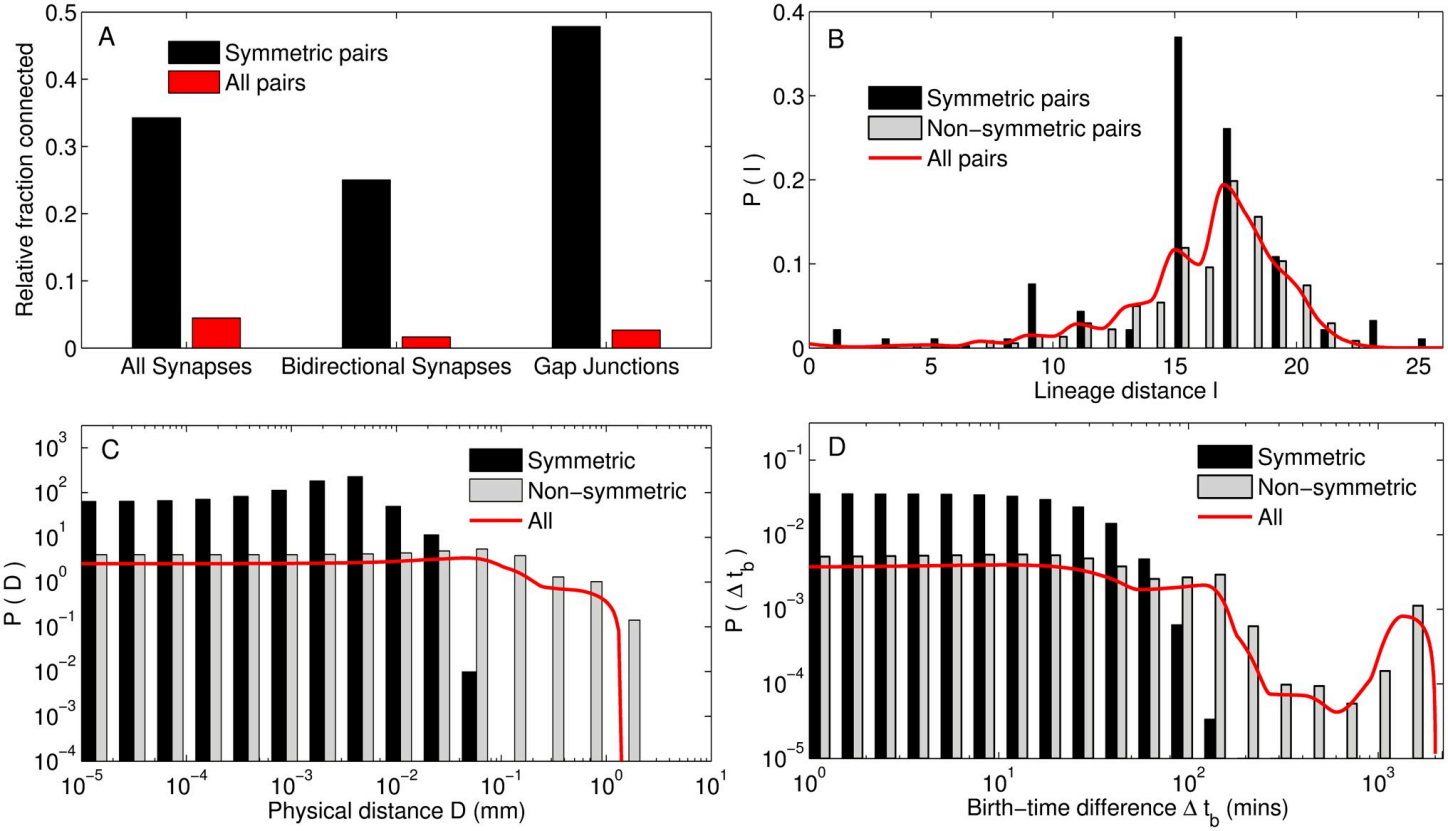
Symmetrically paired neurons have a high probability of being connected and also exhibit strong association in their birth times and spatial positions. (A) Bilaterally symmetric neurons that are positioned on the left and right of the body axis of the organism tend to have a much higher probability of synaptic, as well as, gap junctional connections between them, compared to that for all pairs of neurons. In addition, the synapses are highly likely to be reciprocal (bidirectional). (B) The distribution of lineage distances between paired neurons shows that the mean value is lower than that for all neurons. We note that almost all lineage distances between symmetric neurons are odd-valued suggesting that they occur at the same rung of the lineage tree. (C-D) Symmetrically paired neurons have cell bodies located in physical proximity of each other (C) and are born close in time as indicated by low birth-time differences Δ*t*_*b*_ (D), compared to all pairs of neurons.

Fig 5(A) shows that indeed the left/right members of a symmetric pair have a much higher probability of connection between them than any two arbitrarily chosen neurons belonging to the somatic system. Moreover, 25% of the bilaterally symmetric pairs have reciprocal synaptic connections with each other, compared to less than 2% of all neuronal pairs being connected in such a bidirectional manner. We can further distinguish the symmetric neuron pairs into those which originate from an early division across left/right axis of the common ABp blastomere (i.e., they have similar lineage differing only in the early cell division event ABpl/r) and those where members of a pair originate from non-symmetric blastomeres (e.g., ABal and ABpr) [59]. These two distinct origins of the bilaterally symmetric neurons are reflected in the two peaks of the distribution of lineage distance between the left/right members of each pair seen in Fig 5(B), with only the latter category of paired neurons that do not share a bilaterally symmetric lineage history having low values of *l*. The synaptic connection probability between the members of pairs belonging to these two classes differ only by a small amount (0.34 for the former and 0.35 for the latter, with the corresponding numbers reducing to 0.13 and 0.24, respectively, when we consider reciprocal synapses). The occurrence of gap junctions between bilaterally symmetric neurons is seen to be exceptionally high (47.8% of such pairs being connected) compared to that for the entire system, with no distinction in numbers being observed between the two categories of symmetric pairs. This preponderance of gap-junctional connections between bilaterally symmetric neurons (also indicated by the band diagonal structure of the connectivity matrix shown in panel (B) of Fig 1) suggests that their activity is highly coordinated. This may possibly explain the co-occurrence of both members of a symmetric pair in the different functional circuits.

In addition to exhibiting a high probability of being connected directly, bilaterally symmetric neurons are also characterized by a high degree of neighborhood similarity. S12 Fig in Supporting Information shows the magnitude of overlap between the neurons that each member of a pair is connected by a synapse (either pre- or post-synaptically) or a gap junction, which is seen to be much higher than that for any two arbitrarily chosen neurons. This is consistent with the left/right neurons in the majority of bilaterally symmetric pairs having an identical role in terms of the mesoscopic organization of the network (discussed later, see mesoscopic functional roles). The large number of neighbors that paired neurons share in common is a striking feature that cannot be explained from their physical proximity alone.

We note that almost all lineage distances between symmetric neurons are odd-valued suggesting that they are born at the same rung of the lineage tree. The only exception is the pair AVFL/R, whose members have distinct non-symmetric lineage history, with a lineage distance of 8. Given their shared lineage, it is perhaps unsurprising that most bilaterally symmetric paired neurons also exhibit strong associations in their physical locations and birth times. Panels (C-D) show that a large fraction of the left/right members have cell bodies that are located in close physical proximity of each other (C) and are also born close in time as indicated by low birth-time differences Δ*t*_*b*_ (D), compared to all pairs of somatic neurons. Indeed we note that the only exception is the late-born pair SDQL/R with bilaterally symmetric history whose members are located in the anterior and posterior (respectively) parts of the organism, the physical distance between the cell bodies being 0.5 mm.

#### Relative importance of the different types of homophily in determining the network connectivity

We have demonstrated here the existence of four different types of homophily, i.e., preference of neurons to connect to other neurons having identical or similar attribute(s). We identify these attributes to be (i) process length, (ii) birth cohort, (iii) shared lineage and (iv) bilateral symmetric pairing. While (i) and (ii) are properties characterizing individual cells that allow neurons to be classified into distinct categories, (iii) is measured in terms of the lineage distance between two neurons and is, hence, an attribute of a pair, as is (iv). Thus, in order to quantitatively demonstrate homophily for these four attributes, we have had to use different measures, viz., modularity *Q* in connections between neurons belonging to the same (as opposed to different) categories for (i) and (ii), correlation between connection probabilities and lineage distances for (iii) and comparison of connection probability between symmetrically paired neurons with that for the entire network for (iv). We have also quantitatively established that these attributes are not dependent on each other (see below).

We can now ask about the relative contributions of the four attributes in determining the connectivity of the *C. elegans* nervous system. While, the lack of a common measure means that we cannot directly compare numerical values characterizing these attributes, we can estimate how strongly each of them affect the connection probability by using logistic regression analysis (see Methods). Here, the connection probability *P* between a pair of neurons is expressed as a function of four independent predictor variables *X*_*p*_, *X*_*b*_, *X*_*l*_ and *X*_*s*_ corresponding to the four attributes that show homophily. For this, we first establish that the predictor variables are not correlated by using Belsley collinearity diagnostics [65] (see Methods). The condition indices for all predictors are less than 5, indicating very weak dependencies among them. Furthermore, following the logistic regression analysis we observed that the *p*-values for each of the predictor variables are extremely low (*p* ∼ 0) which is indicative of very high significance for the dependence of the connection probability on all of the predictors. For synaptic connections, the regression coefficients estimated from the empirical data are $\beta_{p}^{syn}=0.35$ (for process length), $\beta_{b}^{syn}=0.71$ (for birth cohort), $\beta_{l}^{syn}=-0.06$ (for lineage relation) and $\beta_{s}^{syn}=1.78$ (for symmetric pairing). Magnitudes of these coefficients indicate the extent by which connection probability is affected upon altering the numerical value of the corresponding predictor variable by a single unit (keeping the other predictors unchanged). Thus, symmetric pairing seems to have the strongest influence in determining the synaptic connection probability. Birth cohort homophily appears to have the next highest contribution followed by process length. Lineage homophily has the weakest contribution, which may be surprising given the almost linear dependence between lineage distance and connection probability [Fig 2(D)]. This is possibly related to the fact that *X*_*l*_ is the only predictor variable whose numerical values are not confined to be binary but instead ranges between 1 and 25. This suggests that the connection probability for a pair of neurons having a lineage distance of 2 will not differ much from that for a pair having lineage distance 3, as compared to, for instance, the difference between neurons belonging to the same and to different birth cohorts. We note that, increasing the lineage distance between a pair of neurons by 6 units would have approximately the same effect on connection probability as the difference in the probability of connections between neurons belonging to the same process length category and different process length categories, given that |*β*_*p*_| ≈ 6 × |*β*_*l*_|. Using similar arguments, we can see that increasing lineage distance by 12 units would lead to an approximately equivalent change in the connection probability as seen between neurons belonging to the same birth cohort and to different cohorts (|*β*_*b*_| ≈ 12 × |*β*_*l*_|). For gap-junctions, the regression coefficients estimated from the empirical data are $\beta_{p}^{gap}=0.22$ (for process length), $\beta_{b}^{gap}=0.16$ (for birth cohort), $\beta_{l}^{gap}=-0.08$ (for lineage relation) and $\beta_{s}^{gap}=3.22$ (for symmetric pairing). Thus, symmetric pairing and lineage relation have the strongest and weakest contributions, respectively, for this case also. However, unlike synapses, process length homophily has a larger effect on gap-junction connection probability than birth cohort homophily.

### Temporal hierarchy of the appearance of neurons during development is associated with their functional identity

We have been focusing, so far, on the various properties related to the developmental history of neurons which govern their spatial organization as well as their inter-connectivity. The latter, as we have shown above, is guided by several types of homophily, i.e., the tendency of neurons which are similar in terms of certain features—viz., process length, lineage, birth-time and bilateral symmetry—to be connected via synapses or gap junctions. We shall now see how the functional identities of neurons are related to their developmental histories. In particular, we show that classes of neurons distinguished by their (i) type (viz., sensory, motor and interneurons), (ii) functional role in the mesoscopic structural organization of the network and (iii) membership in distinct functional circuits, strongly influences the temporal order of their appearance in the developmental chronology of the nervous system.

#### Sensory, inter, and motor neurons

**Fig 6:**
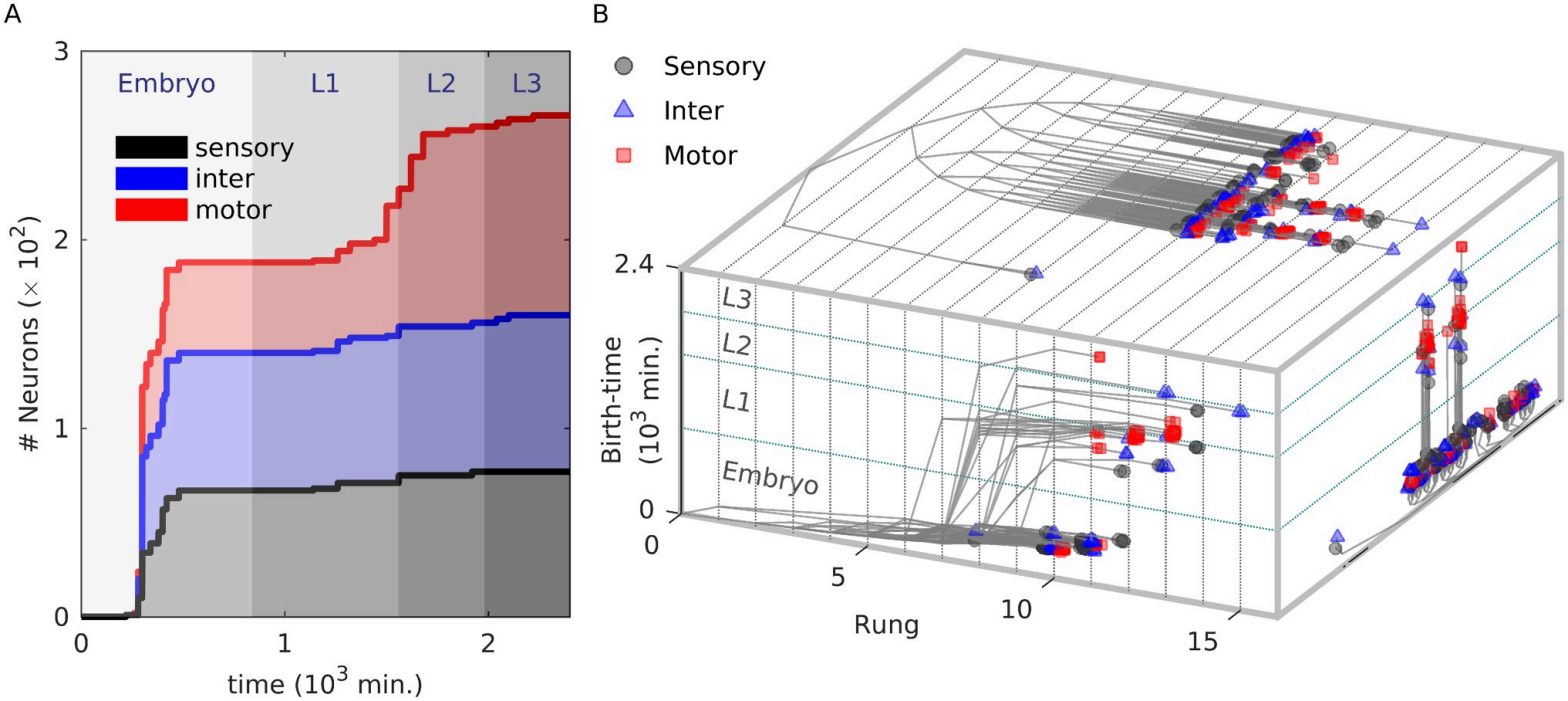
Developmental histories of neurons show a bifurcation into early and late branches, with a predominance of motor neurons in the latter. (A) Bulk of the sensory and interneurons appear early, i.e., during the embryonic stage, while a large fraction of motor neurons differentiate much later (L2 or L3) during development. (B) Planar projections of a three-dimensional representation of the developmental history of the entire somatic nervous system of *C. elegans*. Different colors and symbols have been used to denote distinct neuron types (viz., sensory, motor and interneurons). The projection on the top surface shows the lineage tree with branching lines connecting the single cell zygote (shown at rung 0) to each of the differentiated neurons located on their corresponding rungs. At higher rungs (>11) we see that the differentiated cells are tightly clustered into two bundles of branches with a predominance of motor neurons (also seen in the chrono-dendrogram projection shown at the right face of the base). We note the absence of segregated clusters comprising exclusively the same functional type of neurons (viz., sensory, motor or inter), suggesting that the progenitor cell can give rise to neurons of different types. This in turn implies that commitment to a particular neuron function occurs quite late in the sequence of cell divisions. The projection along the base (left face) shows trajectories representing the developmental history of each final differentiated neuron, indicating the time of each cell division starting from the zygote along with the corresponding rung. For the first few rungs, cell divisions across different lineages appear to be synchronized and occur at regular time intervals, which is manifested as an almost linear relation between time of division and rung. However, between rungs 6-9, we observe a bifurcation of the trajectories into two clusters widely separated in time. One of these comprises cells which differentiate in the embryonic stage (termed as the “early branch”) while the other consists of cells that differentiate much later (“late branch”). This is manifested in a bimodal distribution of birth times for neurons occurring in rungs ≥ 10. In contrast to the regularly spaced cell divisions in the early branch, the trajectories belonging to the late branch are widely dispersed, with relatively little correlation between birth time of neurons and their rungs.

One of the simplest classifications of neurons is according to their position in the hierarchy along which signals travel in the nervous system. Thus, *sensory* neurons receive information from receptors located on the body surface of the organism and transmit them onward to *interneurons*, which allow signals arriving from different parts to be integrated, with appropriate response being eventually communicated to *motor* neurons that activate effectors such as muscle cells. In the mature *C. elegans* somatic nervous system, the motor neurons form the majority (106), while sensory (77) and interneurons (83) are comparable in number. The remaining neurons are polymodal and cannot be uniquely assigned to a specific functional type. In Fig 6(A) we show how the sub-populations corresponding to each of the distinct types evolve over the course of development of the organism. We immediately note that while the bulk of the sensory and interneurons differentiate early, i.e., in the embryonic stage, followed by a more gradual appearance of the few remaining ones in the larval stages, more than half of the motor neurons appear much later after hatching. Moreover, of the 48 motor neurons which appear early, approximately half (23) belong to the nerve ring while the rest are in the ventral cord, where they almost exclusively innervate dorsal muscles (the positions of neurons, classified according to function type and birth time, is shown in Supporting Information, S13 Fig). On the other hand, the 58 late-born motor neurons primarily belong to the ventral cord (with only 4 appearing in the nerve ring). In addition, the majority of them (41) innervate ventral body muscles (see Supporting Information, S4 Table for details). The few (11) late-born motor neurons that do innervate dorsal muscles differ from the early-born ones in that they do not have complementary partners and bring about asymmetric muscle activation [66]. This early innervation of dorsal muscle but late, larval-stage innervation of ventral muscles could embody developmental constraints that deserve further exploration in the future.

Having looked at how neurons emerge according to their functional type at different times and at different locations in the physical space described by the body of the worm, we now consider the appearance of such neurons in the developmental space defined by lineage and birth time [Fig 6(B)]. The projections of the chrono-dendrogram that are shown on the top and the extreme right surfaces, both correspond to representations of the lineage tree that are demarcated by rung and birth time, respectively. We note immediately that the developmental trajectories of the neurons appearing in the late burst of development are clustered into two distinct branches that originate in an early division across left/right axis of the common ABp blastomere (i.e., cells in one branch originate from ABpl, while those in the other emanate from ABpr). Unlike the case seen for neurons belonging to a specific ganglion, we observe that neurons of the same functional type do not form localized clusters in the tree that would have suggested a common ancestry. Thus, progenitor cells can give rise to neurons of each of the different functional types, suggesting that the commitment to a sensory/motor/interneuron fate happens later in the sequence of divisions during development.

The projection on the remaining bounding surface (left face of the base) shows the trajectories followed by cells to their eventual neuronal fate across a space defined by the rung of the lineage tree along one axis and the time of cell division along the other. These trace the developmental history of the entire ensemble of neurons comprising the somatic nervous system. We observe that in the early phase of embryonic stage (corresponding to rungs ≤ 6) there is a linear relation between the time at which a cell divides and the rung occupied by the resulting daughter cells. This implies that cell divisions across different branches of the lineage tree occur at regular time intervals in a synchronized manner. Following this, we observe that the trajectories bifurcate and cluster into two branches that are widely separated in time. The ‘early branch’, which results in cells differentiating to a neuronal fate much before hatching, continues to follow the trend seen in the earlier rungs. However, several progenitor cells (that can occur in rungs ranging between 6 and 9) suspend their division for extremely long times, i.e., until after hatching. These comprise the ‘late branch’ where the final neuronal cell fate is achieved in the larval stages (L1-L3). The occurrence of these two branches gives rise to the bimodal distribution of birth-times shown in Fig 2(B). In contrast to the regular, synchronized cell divisions across different lineages seen in the ‘early branch’, the ‘late branch’ exhibits a relative lack of correlation between rung and birth time, manifested as a wide dispersion of trajectories followed by individual cells. We note that the majority of differentiated neurons that eventually result from the late branch are motor neurons, which corresponds to the late increase in the subpopulation of motor neurons seen in Fig 6(A). Although there is little information as to when synapses form, the late appearance of the majority of the motor neurons could suggest that stimuli from neighboring neurons are playing an important role in shaping their connectivity in comparison to that of sensory and interneurons that are primarily guided by molecular cues.

#### Mesoscopic functional roles

Turning from the intrinsic features of neurons to the properties they acquire as a consequence of the network connection topology, we observe that it has been already noted that neurons that have a large number of connections are born early [36],[35]. This could possibly arise as a result of the longer time available prior to maturation of the organism for connections between these early born neurons to be formed with other neurons, including those that differentiate much later. However, as many neurons which have relatively fewer connections are also born in the early stage, there does not seem to be a simple relation between the degree of a neuron and its place in the developmental chronology. To explore in more depth how the connectivity of a neuron is related to the temporal order of their appearance, we therefore consider the role played by it in the mesoscopic structural organization of the network.

**Fig 7:**
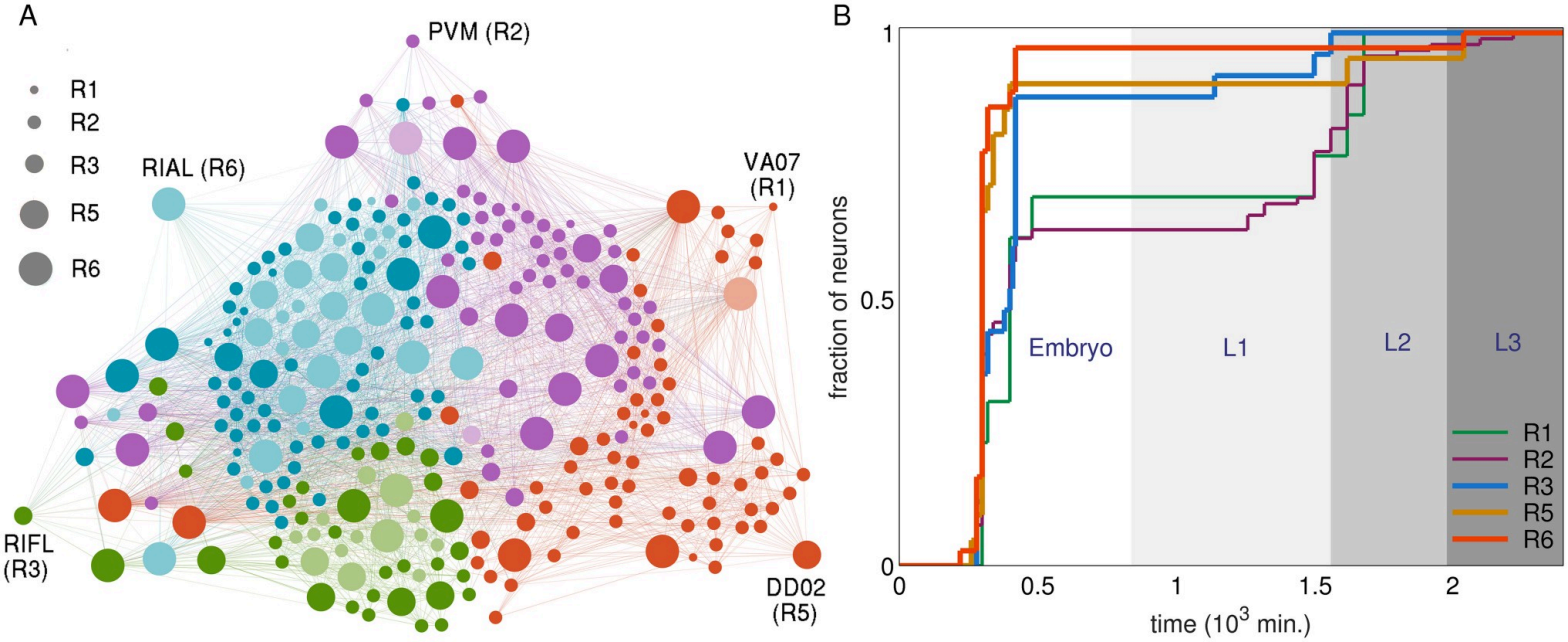
Neurons functioning as connectors between different network modules lead in development. (A) Schematic representation of the network of neurons belonging to the somatic nervous system of *Caenorhabditis elegans*, indicating the role of each neuron (indicated by the node size, see legend) in the mesoscopic structural organization of the network. This organization is manifest in the partitioning of the entire network into six structural modules [34] which are characterized by relatively dense connections among neurons in each module compared to the connections between neurons belonging to different modules (node color representing the identity of a module to which a neuron belongs). Within each module, neurons can be further distinguished into those which have significantly higher number of connections to neurons within their own module (hubs) and those which do not (non-hubs). According to their intra- and inter-modular connectivity, every neuron is then classified into one of seven possible categories (see Methods), viz. R1: ultra-peripheral (non-hub nodes with all their connections confined to their own module), R2: peripheral (non-hub nodes with most of their connections occurring within their module), R3: satellite connectors (non-hub nodes having with many connections to other modules), R4: kin-less (non-hub nodes with connections distributed uniformly among all modules), R5: provincial hubs (hub nodes with a large majority of connections within their module), R6: connector hubs (hub nodes with many connections to other modules) and R7: global hubs (hub nodes with connections distributed uniformly among all modules). One representative neuron from each of the categories is separately indicated with a label identifying them by name (note that there are no neurons in the *C. elegans* somatic nervous system which belong to categories R4 or R7). Neurons which function as connectors, e.g., RIAL (R6) and RIFL (R3), are seen to have links to neurons belonging to many different modules (as indicated by the node color of their network neighborings) while neurons belonging to other categories are connected predominantly to neurons within their own modules (indicated by their network neighborhood being almost homogeneous in terms of node color). Neighbors of labeled neurons are either shown clustered around them (for VA07, PVM, RIFL and DD02) or indicated by a lighter shade of node color (for RIAL). (B) Distributions of differentiation times of neurons belonging to the different network functional role categories indicate that the development of those functioning as connectors and/or hubs (i.e., R3, R5 and R6) lead the other classes of neurons in the embryonic, as well as, L1 stages. In particular, more than 90% of satellite connectors, provincial hubs and connector hubs have appeared before hatching, while for the peripheral categories (R1 and R2), 70% or less of their members would have differentiated by that time.

Specifically, we focus on the six previously identified *topological modules* of the *C. elegans* neuronal network, which are groups of neurons that have markedly more connections with each other than to neurons belonging to other modules [34]. We classify all the neurons by identifying their function in terms of linking the elements belonging to a module, as well as, connecting different modules to each other [67]. This is done by measuring (i) how significantly well connected a neuron is to other cells in its own module by using the within-module degree *z*-score, and (ii) how dispersed the connections of a neuron are among the different modules by using the participation coefficient *P* [68]. Cells are classified as hub or non-hub based on the value of *z* (see Methods for details). The hubs can be further classified based on the value of *P* as (R5) *local or provincial* hubs, that have most of their links confined within their own module and (R6) *connector* hubs, that have a substantial number of their connections distributed among other modules. The measured value of *P* is also used to divide the non-hub neurons into (R1) *ultra-peripheral* nodes, which connect only to members of their own module, (R2) *peripheral* nodes, most of whose links are restricted within their module and (R3) *satellite connectors*, that link to a reasonably high number of neurons outside their module. Fig 7(A) shows the roles (indicated by node size) played by each neuron in the somatic nervous system of *C. elegans* using a schematic representation of the network. In principle, while it is also possible to have (R7) *global* hubs and (R4) *kinless* nodes, viz., hub and non-hub nodes that may connect to other neurons homogeneously, regardless of their module, none of the neurons appear to play such roles in the network.

Earlier investigation [34] has already established that the connector hubs are crucial in coordinating most of the vital functions that the *C. elegans* nervous system has to perform. Their importance to the network is further reinforced by observing from Fig 7(B) that all but one of the neurons belonging to the R6 category appear early in the embryonic stage, exception being PVNR, which differentiates in the L3 stage. About 90% of (R3) *satellite connectors* and (R5) *provincial* hubs are differentiated before hatching. By contrast, peripheral categories (R1) and (R2) have a much smaller fraction of their members appear in the early burst of development and have to wait till the L1, L2 or L3 stage for the development of their full complement. In particular, *satellite connectors* (R3) in spite of having relatively lower degrees than (R5) or (R6) hubs, develop at par with the hubs. Previous studies suggested that hubs, having high degrees, are expected to develop early [36], but *satellite connectors* developing as early as hubs suggests that not only the degree, but also the distribution of the connections of a neuron among the different modules (quantified by the participation coefficient *P*), and thus its functional role in coordinating activity across different parts of the network, which is an important determinative factor for its appearance early in the developmental chronology of the nervous system.

#### Membership in functional circuits

**Fig 8:**
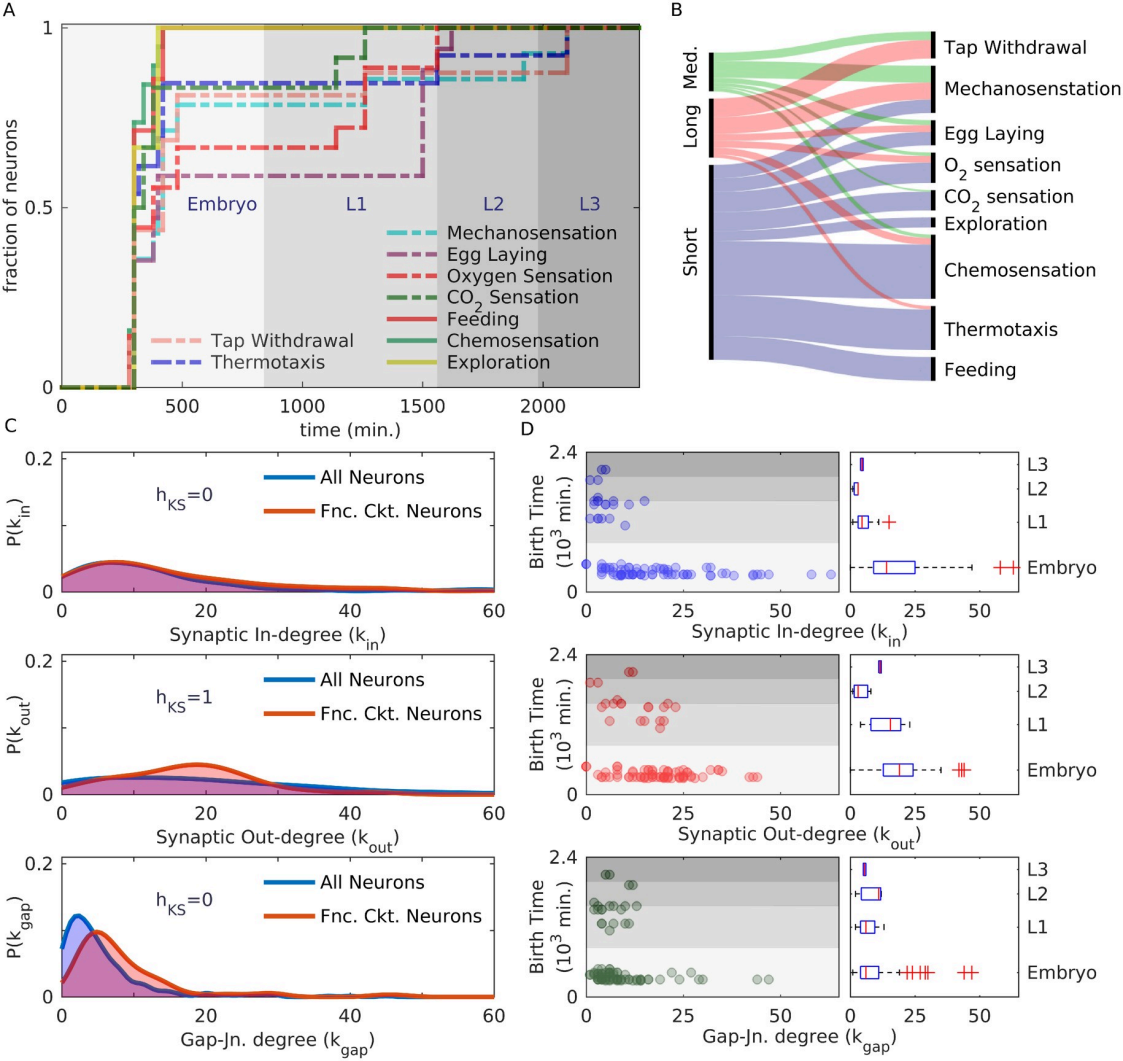
The developmental duration of functional circuit neurons are strongly indicative of their process length and connectivity. (A) Distribution of differentiation times of neurons that belong to any of nine functional circuits identified from behavioral assays. Note that the entire complement of neurons belonging to three functional circuits [shown using solid lines] have differentiated before hatching, while those for others [shown using broken lines] are completed later. (B) The distribution of neurons having short, medium and long processes [indicated at left], among the different functional circuits [right]. We note a correlation between the morphological feature of neurite length and the development time of functional circuit neurons, viz., those in the circuits completed before hatching predominantly have short processes, while those in circuits that are completed later mostly have medium to long processes (the exceptions being thermotaxis and *CO*_2_ sensation circuits that comprise a majority of short process neurons). (C) Comparison between the distributions of the number of incoming and outgoing synaptic connections (*k*_*in*_ [top panel] and *k*_*out*_ [middle panel], respectively), as well as, gap junctions (*k*_*gap*_ [bottom panel]) of neurons in the entire somatic nervous system (blue) and of the subset of functional circuit neurons (red). We note that the distribution of outgoing synaptic connections for the functional circuit neurons is significantly different from that for the entire network, as indicated by the result of a two-sample Kolmogorov-Smirnov test at 1% level of significance (*h*_*KS*_ = 1), but this is not the case for incoming synaptic connections or gap junctions (*h*_*KS*_ = 0). (D) Dispersion of *k*_*in*_ (top panel), *k*_*out*_ (middle) and *k*_*gap*_ (bottom) for the functional circuit neurons differentiating at various times is shown in terms of the adjoining box plots where neurons are clustered into four groups according to the developmental stage during which they are born, viz., Embryo, L1, L2 or L3. In general, the distributions are far more broad for the early born neurons (Embryo) compared to those born later (L1-L3). Focusing on the functional circuit neurons that develop in the embryonic stage, we note that the distribution of incoming connections is more skewed than that for outgoing connections. The distribution of gap junctions is even more skewed, with outliers lying very far from the median.

In order to delve deeper into a possible association between the function(s) that a neuron performs in the mature nervous system and its developmental characteristics, specifically its place in the temporal sequence of appearance of the neurons, we now focus on several previously identified functional circuits of *C. elegans* [61–63]. These are groups of neurons which have been identified by behavioral assay of individuals in which the cells have been removed (e.g., by laser ablation). As their absence results in abnormal or impaired performance of specific functions, these neurons are believed to be crucial for executing those functions, viz., (F1) mechanosensation [45–47], (F2) egg laying [69–71], (F3) thermotaxis [72–74], (F4) chemosensation [48], (F5) feeding [10, 45, 75], (F6) exploration [10, 45, 75] (F7) tap withdrawal [46, 76], (F8) oxygen sensation [77, 78] and (F9) carbon dioxide (*CO*_2_) sensation [79, 80] Note that several neurons belong to multiple functional circuits. Fig 8(A) shows that one can classify these nine functional circuits into two groups based on whether or not all the constituent neurons of a circuit appear during the early burst of development in the embryonic stage. Thus, while circuits for F4-F6 (shown using solid lines in the figure) have their entire complement of cells differentiate prior to hatching, circuits for F1-F3 and F8-F9 lag behind (broken lines), with less than 60% of the egg laying circuit having appeared at the time of hatching. Indeed, for the entire set of neurons for the latter circuits to emerge one has to wait until the late L1 (for F8 and F9), L2 (for F2) or L3 (for F1, F3 and F7) stages [note that out of the 16 neurons in the F7 circuit, 15 are common to those belonging in the F1 circuit, making the former almost a subset of the latter]. While it makes intuitive sense that egg laying circuit does not have all its components in place by the time of hatching (as the function is required only in the adult), it may appear surprising that many circuits mediating functions vital for the survival of the organism (such as mechanosensation, thermotaxis and oxygen sensation) are not completed at the embryonic stage itself. However, upon taking a closer look at the late-born neurons belonging to these functional circuits we note that even though these neurons are essential for the normal execution of the corresponding function, their absence have also been individually shown not to result in a significant decline in the function. For example, it has been shown that the ablation of the late born neurons PVDL/R and AVM, which belong to both the tap withdrawal and mechanosensation circuits, do not significantly impair the response of the worm to a tap stimulus [46]. The same is the case for the late born neurons PDEL/R and PVM belonging to the mechanosensation circuit, whose removal does not significantly affect touch response [76]. The late born neurons PLNL/R and SDQL/R in the oxygen sensation circuit and PQR and AQR belonging to the *CO*_2_ sensation circuits have all been identified as “minor” sensory neurons for the respective gases [81]. It is to be noted that the principal sensory neurons belonging to both of these circuits appear before the worm hatches. In the thermotaxis circuit, the ablation of the late born neurons PVDL/R have been shown not to cause any significant impairment of normal thermotactic behavior [74]. While the thermosensory neurons PHCL/R at the tail that are also born late are indeed essential for thermal avoidance behavior [74], the corresponding neurons FLPL/R in the head are present before hatching ensuring that thermosensory behavior of the worm is not seriously compromised immediately after hatching. Thus, the apparent paradox of how the worm manages to survive after hatching even though several of its functionally critical circuits are not yet complete by that time, is answered by the fact that the role of the late-born neurons belonging to these circuits is often relatively minor. Possibly the sole exception is the egg-laying circuit, in which some of the motor neurons (VC1, VC2, VC3, VC4, VC5) responsible for generating muscle movement appear only after hatching.

An intriguing relation between process lengths of neurons and their occurrence in different functional circuits is suggested by Fig 8(B), from which we see that circuits which have their entire complement of neurons differentiate early, viz., F5-F7, are dominated by neurons having short processes. In contrast, circuits such as F1, F2, F7 and F8 that take much longer to have all their members appear comprise a large number of neurons with medium or long processes (the exceptions being circuits F3 and F9 that have predominantly short neurons). This association between a morphological feature (viz., neurite length) of a functionally important neuron and its time of appearance suggests a possible connection with the process length homophily, viz., preferential connection between neurons having short processes, mentioned earlier. Neurons with short processes that belong to the “early” functional circuits are mostly chemosensory or interneurons that are all located in the head region. To perform their task these neurons only need to connect to each other, whose cell bodies are mostly in close physical proximity of each other. Moreover, having their synapses localized within a small region allows them to be activated by neuromodulation through diffusion of peptides and other molecules [82, 83]. This assumes significance in light of our observation that process length homophily between short process neurons is marginally enhanced in the head. The value of *Q*, a quantitative measure of homophily introduced earlier, is 0.1 (for synapse, for gap junctions it is 0.13) for early born short process neurons which have their cell bodies located in the head region. In contrast, when we consider all short process neurons, *Q* is 0.067 for synapse and 0.085 for gap junction, respectively. Thus, the process length homophily we reported earlier could arise in short process neurons because of functional reasons.

We shall now see how consideration of functional circuits help in obtaining a deeper understanding of the nuanced relation between the degree of a neuron and the time of its birth that was discussed above (in the context of mesoscopic functional roles of neurons). As seen in Fig 8(C), neurons belonging to the functional circuits show a significantly different distribution for the number of synaptic connections (both incoming and outgoing) from that of the entire system, as indicated by the results of two-sample Kolmogorov-Smirnov test (test statistic *h*_*KS*_ = 1) at 1% level of significance. Thus, the set of functionally critical neurons—which, on average, have a larger number of connections than a typical neuron in the somatic nervous system—may need to be treated separately from the other neurons when we examine how synaptic degree correlates with birth time. In contrast, their gap junctional degree distributions cannot be distinguished from that of all neurons, as indicated by the result (*h*_*KS*_ = 0) for the statistical test of significance.

Considering only the neurons that appear in functional circuits, we observe that most of the neurons having a large number of synaptic connections (particularly, incoming ones) do tend to appear early [Fig 8(D)]. On comparing the distributions of synaptic in-degree separately for early and late appearing functionally critical neurons (see Supporting Information, S14 Fig) we note that their difference is indeed statistically significant. When we consider the distribution of the synaptic out-degree we see a very different result. The distributions for the early and late born functionally critical neurons turn out to be statistically indistinguishable (despite the appearance of a few extreme outliers such as the command interneurons AVAL/R. In contrast, the rest of the neurons show a much broader distribution (statistically distinguishable using a two-sample Kolmogorov-Smirnov test) for the neurons that are born early, compared to those which are born late. This is consistent with the assumption that pre-synaptic neurons that exist for a longer period during development, are able to form many more connections than those neurons which appear later (the latter presumably having less time to form connections before the maturation of the nervous system). From this perspective, it is thus striking that the late born functionally critical neurons have as many connections as they do (making them statistically indistinguishable from the early born set), and is possibly related to their inclusion in the functional circuits.

When we consider the gap-junctional degree distributions, we observe that there is no statistically significant difference between the distributions for early and late born neurons, whether they be functionally critical or other neurons. The box plots showing the nature of the distribution at different developmental stages are all fairly narrow [bottom panel of Fig 8(D)], even though the embryonic one shows several outliers with the four farthest ones being the command interneurons AVAL/R and AVBL/R that appear in four functional circuits, viz., those of mechanosensation, tap withdrawal, chemosensation and thermotaxis. These, in fact, correspond to the outlying peaks of the *k*_*gap*_ distribution, located on the extended tail at the right of the bulk [bottom panel of Fig 8(C)]. Indeed, the outliers in each of the distributions (for *k*_*in*_, *k*_*out*_ and *k*_*gap*_), that appear only at the embryonic stage, almost always happen to be command interneurons. Of these, AVAL/R are common across the distributions and the fact that they occur in four of the known functional circuits underlines the relation between function, connectivity and the temporal order of appearance of neurons that we have sought to establish in this paper.

## Discussion

The nervous system, characterized by highly organized patterns of interactions between neurons and associated cells, is possibly the most complex of all organ systems that is assembled in an animal embryo over the course of development [84]. For this neural network to be functional, it is vital that the cells are able to form precisely delineated connections with other cells that will give rise to specific actions. This raises the question of how the “brain wiring problem” is resolved during the development process of an organism. In addition to the processes of cellular differentiation, morphogenesis and migration that are also seen in other tissue, cells in the nervous system are also capable of activity which modulates the development of the neighboring cells they may interact with. Processes extending from the neuronal cell bodies are guided towards designated targets by molecular cues, and the resulting connections are subsequently refined (e.g., by pruning) through the activity of the cells themselves. In this paper we have looked at a more abstract level of guiding principles that can help in connecting the details of cellular wiring at the implementation level of molecular mechanisms with the final result, viz., the spatial organization and connection topology of an entire nervous system. Using the relatively simple nervous system of the model organism *Caenorhabditis elegans*, whose entire developmental lineage and connectivity are completely mapped, we have striven to show how development itself provides constraints for the design of the nervous system.

One of our key findings is that neurons with similar attributes, specifically, (i) the lengths of the processes extending from the cell body (short/ medium/ long), (ii) the birth cohort to which they belong (early/ late), (iii) the extent of shared lineage and (iv) bilateral symmetry pairing (left/ right), exhibit a significant preference for connecting to each other (homophily). Moreover, each of these are manifested by both the connection topology of the network of chemical synapses, as well as, that of electrical gap-junctions, despite the fundamental differences in the nature of these distinct types of links.

We have already discussed earlier a plausible mechanism by which homophily based on lineage would be observed. As neurons are displaced from their initial locations while retaining their connections that have formed already, cells that share common lineage tend to move apart. An alternative possibility that may explain lineage homophily for synaptic connections is related to the suggestion that synaptogenesis could be guided by cellular labels that are specified by a combinatorial code of neural cell adhesion proteins [18]. In this scenario, cells that are close in terms of their lineage will be likely to share several of the recognition molecules that will together determine the label code. Thus, if a sufficiently large number of these determinants match each other, it could promote synapse formation between such cells, resulting in lineage homophily. We would also like to note that, apart from playing an important role in determining the topological structure of the synaptic network, lineage relations between neurons also appear to shape the spatial organization of neurons by segregating them into different ganglia.

In addition to investigating the probability that a connection will occur between a pair of neurons during development, our study also considers how the distance between cell bodies of the neurons thus connected is distributed. Our results suggest that for synapses, the process length of the pre-synaptic neuron is a decisive factor in determining the separation that is allowed between the neuronal partners. Birth time also appears to play a role, particularly, in the case of long-range connections, i.e., between neurons whose cell bodies are separated by more than two-thirds of the worm body length. Specifically, such connections occur between pre-synaptic neurons that are born early and post-synaptic neurons that are born late, much more often than is expected by chance. This suggests the existence of an active process for the formation of such long-range connections, for example, using fasciculation as an axon guidance mechanism [9, 10]. The latter involves a few pioneer neurons with long process lengths acting as supporting pathways that guide axons of the later developing neurons. This may also underlie a triadic closure-like phenomenon in the network [51, 85] (viz., two neurons having links to one or more common neighbors that have an increased likelihood of being connected to each other). Such a process is known to yield strongly clustered networks with high communication efficiency [86, 87] and could be responsible for the appearance of the so-called “common neighbor rule” that has been reported in the *C. elegans* connectome [88].

Our results also indicate that the temporal sequence in which neurons appear during development of the nervous system is linked to their functional identities. The simplest of these identities is simply the basic functional type of a neuron, viz., whether it is sensory, motor or interneuron. The neurons belonging to these different types not only differ in terms of the cells they connect to (for instance, only motor neurons connect to muscles and sensory neurons are the only ones to receive connections from receptors, while interneurons connect to all types of neurons) but also in their molecular inventory. While their lineage does not show any significant differences, the different functional types of neurons do appear to segregate to a certain extent in terms of their time of appearance. Specifically, we find that the bulk of neurons that are born in the late, post-embryonic burst of development are motor neurons. Our results suggest that there may be temporal cues that appear late in the process of development which are responsible for the specialization of neurons into different functional types according to their time of birth.

At a higher, mesoscopic level of organization of the network structure, we have considered the functional role of neurons in coordinating the activity of different topological modules of the network. We show that this allows us to obtain a much more nuanced picture of how the number of connections that a neuron has with other neurons, affects its place in the temporal sequence in which neurons appear during development. Thus, rather than a simple case of just the degree (the total number of connections) of a neuron deciding its precedence in the sequence, it is both the hub (provincial, as well as, connector hubs) and the connector neurons (satellite connectors, in addition to the connector hubs) that appear early. We also examine in detail the subset of neurons that have been identified as belonging to one or more functional circuits in the *C. elegans*. We observe that membership of a specific functional circuit does determine the order in which these neurons appear, with certain circuits, such as those responsible for chemosensation, emerging early (before hatching) while others circuits, such as those for mechanosensation and egg-laying, appear much later (after hatching). In turn, the time of appearance of functional circuits determines to an extent the morphological properties, such as the process lengths, of their constituent neurons.

The observations reported in this paper are an attempt at resolving the “wiring problem” for the *C. elegans* nervous system by focusing at a level that is intermediate between the molecular mechanism-level details of developmental processes and the resulting structural organization of the entire somatic nervous system. Specifically, we have attempted to uncover general strategic principles governing the design of the neuronal network, which will allow linking the complicated molecular machinery involved to the equally complicated spatial and topological description of the nervous system. The next step in this approach will involve delineating exactly how these governing principles (such as the various types of homophily) are implemented by molecular mechanisms, and how genetics may be relating the temporal sequence of appearance of neurons to their functional identities. Experimental and theoretical progress towards this direction would enable us to achieve a seamless understanding of nervous system development involving different scales.

## Materials and methods

### Data

#### Connectivity

Information about the connections between different neurons obtained using serial section electron micrography were first reported in Ref. [10]. Subsequent analysis of the images has led to discovery of many more connections which have been published in Refs. [11] and [89] (which differ marginally in the connections they report). A more recent re-analysis has added further connections to the connectome [90]. We have used the information about connectivity between 279 connected neurons of the *C. elegans* nervous system from the latest dataset which is published in Ref. [90]. We have explicitly verified that our results remain substantially unchanged on using the earlier connectome dataset from Ref. [11]. The differences between the connectivity reported in the above-mentioned databases are visually represented in the Supporting Information, S15 and S16 Figs.

#### Lineage

We have used information about the lineage distance between 279 connected neurons of the *C. elegans* somatic nervous system from the database published in Ref. [11], accessible from an online resource for behavioral and structural anatomy of the worm [81].

#### Time of birth for neurons

We have transcribed the time of appearance of each neuron over the course of development of the organism from lineage charts provided in Refs. [16, 17]. This is provided in S5 Table in the Supporting Information.

#### Time of cell-division for progenitor cells

The information about the time of each cell-division, starting from the zygote, that occurs over the course of development of the *C. elegans* somatic nervous system, and which has been used for generating the chrono-dendrograms shown here, are provided in Refs. [16, 17], accessible from an online interactive visualization application [91].

#### Physical distance

We have used information on the positions of the neurons from the database reported in Ref. [92], accessible online from https://www.dynamic-connectome.org/. The location information provides coordinates of each neuronal cell body projected on a two-dimensional plane defined by the anterior-posterior axis and the dorsal-ventral axis.

#### Neuronal process length

Lengths of the processes extending from each cell body has been estimated from the diagrams of the neurons provided in Appendix 2, Part A of Ref. [55] and from an online resource for the anatomy of the worm [81].

#### Ganglia and functional types

The information about the ganglion to which a neuron belongs and the functional type of each neuron (viz., sensory, motor or interneuron) has been obtained from the database provided in Ref. [93].

#### Functional circuits

The identities of the neurons belonging to each of the functional circuits have been obtained from the original references for each circuit. The functional circuits analyzed in this paper and their respective original references are as follows: (F1) mechanosensation [45–47], (F2) egg laying [69–71], (F3) thermotaxis [72–74], (F4) chemosensation [48], (F5) feeding [10, 45, 75], (F6) exploration [10, 45, 75] (F7) tap withdrawal [46, 76], (F8) oxygen sensation [77, 78] and (F9) carbon dioxide (*CO*_2_) sensation [79, 80] The neuronal composition of each of these functional circuits is given in Supporting Information, S7 Table.

### Modularity

A network can be partitioned into several communities or topological modules, defined such that neurons in a given module have a much higher probability of being connected to other neurons in the module compared to neurons that do not belong to it, by maximizing the modularity value *Q* [50] associated with a given partitioning, viz.,

$$\begin{array}{c} Q=\frac{1}{L}\sum_{i,j}[A_{ij}-\frac{k_{i}^{in}k_{j}^{out}}{L}]\delta_{c_{i}c_{j}}. \end{array}$$ (1)

Here, **A** is the adjacency matrix describing the connections of the network (*A*_*ij*_ = 1, if neuron *i* receives a connection from neuron *j*, and = 0 otherwise). The in-degree and out-degree of a node *i* are given by $k_{i}^{in}=\sum_{j}A_{ij}$ and $k_{j}^{out}=\sum_{i}A_{ij}$, respectively. The total number of links in the network is given by *L* = ∑_*i*,*j*_ *A*_*ij*_. The Kronecker delta function *δ*_*ij*_ = 1, if *i* = *j*, and 0, otherwise. The indices *c*_*i*_, *c*_*j*_ refer to the modules to which the neurons *i* and *j*, respectively, belong. For an undirected network, such as that defined by the set of connections between neurons via gap-junctions, the adjacency matrix is symmetric (i.e., *A*_*ij*_ = *A*_*ji*_) and $k_{i}^{in}=k_{i}^{out}=k_{i}$. The value of *Q* expresses the bias that a neuron has to connect to members of its own community (which could be defined in terms of any distinguishing characteristic of the cells, e.g., process length), relative to the null model. The latter corresponds to an unbiased, homogeneous network where the probability of connection between two nodes is proportional to the product of their respective degrees. A positive value of *Q* that is significantly higher than that obtained from an ensemble of randomized surrogate networks would indicate the existence of homophily.

To further establish homophily, we also show that the bias for connecting to neurons belonging to a particular class (e.g., short process length) can be observed only among neurons in that class, and not between neurons belonging to different classes (e.g., between short and long process neurons). For this, we define *Q* values for each pair of classes (*X* and *Y*, say) as follows:

$$\begin{array}{c} Q_{XY}=\frac{1}{L}\sum_{i,j}[A_{ij}-\frac{k_{i}^{in}k_{j}^{out}}{L}]\delta_{c_{i}Y}\cdot \delta_{c_{j}X}, \end{array}$$ (2)

where the symbols have the same meanings as in Eq 1. Note that, the *Q* for the entire network calculated in terms of Eq 1 is related to these class-specific modularity values as *Q* = ∑_*X*∈all classes_ *Q_XX_*. Homophily is established upon demonstrating that the values of *Q*_*XX*_, *Q*_*YY*_, etc. are significantly higher than that of *Q*_*XY*_, *Q*_*YX*_, etc.

### Bimodality coefficient

The bimodal nature of a probability distribution can be characterized by calculating its bimodality coefficient [52]:

$$\begin{array}{c} BC=\frac{m_{3}^{2}+1}{m_{4}+3\cdot \frac{{\left(n-1\right)}^{2}}{\left(n-2)(n-3\right)}}, \end{array}$$ (3)

where *m*_3_ is the skewness, *m*_4_ is the excess kurtosis and *n* represents the sample size. A distribution is considered to be bimodal if *BC* > BC* where BC* = 5/9. This benchmark value corresponds to a uniform distribution, and if *BC* < BC*, the distribution is considered unimodal.

### Process length randomization

To establish statistically significant evidence for process length homophily, the empirical network is compared with an ensemble of networks obtained from the empirical one by randomly assigning process lengths (short, medium and long) to the neurons while ensuring that the total number of neurons in each process length category, viz., *N*_*S*_, *N*_*M*_ and *N*_*L*_, respectively, (as well as, all other properties of the network, such as connectivity) remains unchanged. In practice, this is done by first partitioning the neurons into three communities according to process length and ordering the neurons in sequence according to the module they belong. Thus, neurons *i* = 1, …, *N*_*S*_ have short processes, neurons *i* = *N*_*S*_ + 1, …, *N*_*S*_ + *N*_*M*_ have medium length processes, and neurons *i* = *N*_*S*_ + *N*_*M*_ + 1, …*N*_*S*_ + *N*_*M*_ + *N*_*L*_ have long processes. Then, to create each member of the surrogate ensemble, this sequence is randomly permuted and the first *N*_*S*_ neurons are assigned short process length, the next *N*_*M*_ neurons are assigned medium process length and the remaining *N*_*L*_ neurons are assigned long process length. The modularity *Q* calculated for networks with such randomized module membership (corresponding to a null model where process length homophily is non-existent by design) is expected to be small.

### Network randomization constrained by neuronal process lengths

An ensemble of surrogate networks is constructed by randomizing the connections of the empirical network, subject to different constraints. Each member of the ensemble is constructed by repeatedly selecting a pair of directed connections, e.g., *p* → *q* and *u* → *v*, and rewiring them such that the in-degree and out-degree of each neuron remains invariant, i.e., *p* → *v* and *u* → *q*. If these new connections already exist, this rewiring is disallowed and a new random selection for a pair of directed connections done. In addition, information about the spatial location of the cell bodies and that of the process lengths of neurons are used to further constrain the connections. This ensures that un-physical connections, such as between two short process neurons (i.e., each has a process length that is less than a third of the body length of the nematode) whose cell bodies are placed apart by more than 2*L*/3 (*L*: total body length of the worm), do not appear through the randomization. In practice, this constraint is imposed as follows. In absence of precise knowledge of the length of each process, depending on the length process category to which each neuron belongs, an uniformly distributed value (lying between [0, *L*/3] for short, between [*L*/3, 2*L*/3] for medium and [2*L*/3, *L*] for long process neurons) is assigned as the process length for a neuron. The distance between the cell bodies of a pair of neurons that have been selected randomly for connection is then compared against the sum of their process lengths. If the latter is greater than the former, the connection is allowed, else not. The rewiring steps are repeated 5 × 10^5^ times to construct each of the randomized networks belonging to the surrogate ensemble. The entire ensemble consists of 100 realizations of such randomized networks.

### Lineage randomization

To establish that neurons belonging to the same ganglion are closely related in terms of their lineage, we compare the properties of the lineage distance distribution within and between ganglia obtained for the empirical network with those obtained upon randomizing the lineage relations. This is done by repeatedly selecting a pair of neurons at random on the lineage tree and exchanging their positions on the tree. This procedure is carried out 10^4^ times for a single realization. This ensures that, in the randomized networks, the lineage relation between neurons is completely independent of whether they belong to the same ganglion or not. In order to compare the properties of the empirical network with its randomized version, an ensemble of 10^3^ realizations is considered. To quantify the deviation of the empirical intra- and inter-ganglionic lineage distance distributions from their randomized counterparts, we measure the *z*-score of the corresponding means and coefficients of variation (CV). The *z*-score is a measure for the extent of deviation of an empirical property *x*_*emp*_ from the mean of the randomized counterparts, 〈*x*_*rand*_〉, scaled by the standard deviation of the randomized counterparts, viz.,

$$\begin{array}{c} z=\frac{x_{emp}-\left\langle x_{rand}\right\rangle }{\sqrt{\left\langle x_{rand}^{2}\right\rangle -{\left\langle x_{rand}\right\rangle }^{2}}}. \end{array}$$ (4)

### Surrogate ensemble for comparison with average cell body distance between connected neuronal pairs

To see whether the distance *d* between cell bodies of connected pairs of neurons [where the members of the pair could belong to either the same or different process length categories, viz., short (S), medium (M) and long (L)] is distributed in a significantly different manner from that between all pairs of neurons, we have constructed surrogate ensembles. For each realization belonging to such an ensemble, a number of cell body distances is sampled from the set of all distances *D* between each neuronal pair, such that the sample size is same as the number of connected neural pairs. The entire ensemble consists of 10^3^ such sampled sets. To see whether the observed difference between 〈*d*_*XY*_〉 and 〈*D*〉_*X*,*Y*_, where *X*, *Y* ∈ {*S*, *M*, *L*}, can be explained simply as finite size fluctuation, we have evaluated the corresponding *z*-scores, viz.,

$$\begin{array}{c} z_{XY}=\frac{d_{XY}^{emp}-\left\langle d_{XY}^{rand}\right\rangle }{\sqrt{\left\langle {\left(d_{XY}^{rand}\right)}^{2}\right\rangle -{\left\langle d_{XY}^{rand}\right\rangle }^{2}}}. \end{array}$$ (5)

### Lineage tree rung determination

The order of the rung in the lineage tree that a cell belongs to is obtained from the lineage information of the cell (available from Ref. [81]). This indicates the series of cell divisions, starting from AB (which results from the division of the single cell zygote) that leads to a particular neuron, e.g., ABprpapaap. The letters a (anterior), p (posterior), l (left) and r (right) which follow AB, indicate the identity of the progenitor cells that result from subsequent cell divisions eventually terminating in a differentiated neuron. As the rung that a neuron belongs to is given by the number of cell divisions (starting from the zygote) that leads to the differentiated cell, we simply count the total number of letters (AB is counted as a single letter) specifying the lineage of a cell to determines its rung.

### Stochastic branching model for lineage tree

To theoretically describe the generative process leading to the observed lineage tree for the cells belonging to the *C. elegans* somatic nervous system, we have used a stochastic asymmetric branching model. Starting from the single cell zygote, each cell division leads to at most two daughter cells, with independent probabilities *P*1 and *P*2 (*P*1 ≥ *P*2) for the occurrence of each of the two branches. Thus, based on the probabilities *P*1 and *P*2, at each step of the generative process any one of the following three events can happen: (i) proliferation occurs along both branches, (ii) only one branch appears (the other branch leading to either apoptosis or a non-neural cell fate), and, (iii) there is no branching so that a terminal node of the tree is obtained (i.e., the cell differentiates into a neuron). Estimation of *P*1 and *P*2 from the empirical lineage tree suggests that proliferation markedly reduces after rung 10. Incorporating this in the model by decreasing the probabilities *P*1, *P*2 after rung 10 results in successive reduction of the branching, eventually coming to a stop. The ensemble of 10^3^ simulated lineage trees produced by the process matches fairly well with the empirical lineage tree in terms of the number of terminal nodes, the distribution of the rungs occupied by each cell and the distribution of lineage distances between the differentiated neurons (see Supporting Information, S6 Fig).

### Classifying neurons according to their role in the mesoscopic structural organization of the network

The functional importance of a neuron *vis-a-vis* its own topological module (defined above in Sec. 1), as well as, the entire nervous system, can be quantified in terms of its intra- and inter-modular connectivity [34]. For this purpose we use the two metrics [67]: (i) the within module degree *z*-score (*z*) and (ii) the participation coefficient (*P*).

In order to identify neurons that have a significantly large number of connections to the other neurons belonging to their module, we calculate the within module degree *z*-score defined as

$$\begin{array}{c} z_{i}=\frac{\kappa_{c_{i}}^{i}-{\left\langle \kappa_{c_{i}}^{j}\right\rangle }_{j\in c_{i}}}{\sqrt{{\left\langle {\left(\kappa_{c_{i}}^{j}\right)}^{2}\right\rangle }_{j\in c_{i}}-{\left\langle \kappa_{c_{i}}^{j}\right\rangle }_{j\in c_{i}}^{2}}}, \end{array}$$ (6)

where $\kappa_{c}^{i}$ is the number of connections that a neuron *i* has to other neurons in its community (labeled *c*) and the average 〈…〉_*j* ∈ *c*_ is taken over all nodes in the community. Following Ref. [34], we identify neurons having *z* ≥ 0.7 as hubs, while the remaining are designated as non-hubs.

The neurons are also distinguished in terms of how many well connected they are to neurons belonging to other communities. For this purpose we measure the participation coefficient *P* of a neuron, which is defined as

$$\begin{array}{c} P_{i}=1-\sum_{c=1}^{m}{\left(\frac{\kappa_{c}^{i}}{k_{i}}\right)}^{2}, \end{array}$$ (7)

where $\kappa_{c}^{i}$, as above, is the number of connections that the neuron has to other neurons in its own module (labeled *c*) and $k_{i}=\sum_{c}\kappa_{c}^{i}$ is the total degree of node *i*. Neurons that have their connections homogeneously distributed among all modules will have a *P* close to 1, while *P* = 0 if all of their connections are confined within their module. Based on the value of *P*, following Ref. [34] we have classified the non-hub neurons as ultra-peripheral (R1: *P* ≤ 0.05), peripheral (R2: 0.05 < *P* ≤ 0.62), satellite connectors (R3: 0.62 < *P* ≤ 0.8) and kin-less nodes (R4: *P* > 0.8), while hub neurons are segregated into provincial hubs (R5: *P* ≤ 0.3), connector hubs (R6: 0.3 < *P* ≤ 0.75) and global hubs (R7: *P* > 0.75).

### Logistic regression

We have employed logistic regression [94] to assess the relative contributions of different factors in determining the probability of connection between a pair of neurons. Here, the probability of occurrence of an event is expressed as a function of one or several independent predictor variables. In our case, the event *Y* denotes the presence (*Y* = 1) or absence (*Y* = 0) of a connection between two neurons. The independent predictor variables correspond to the attributes: (i) process length (*X*_*p*_ = 1 if the process lengths of the two neurons are in the same class [short, medium or long], = 0 otherwise), (ii) birth cohort *X*_*b*_ (= 1 if both neurons are born early or both are born late, = 0 otherwise), (iii) symmetric pairing *X*_*s*_ (= 1 if the two neurons correspond to a symmetric pair, = 0 otherwise) and (iv) lineage relation *X*_*l*_ (= lineage distance *l* ∈ {1, 2, ….25}). Thus, logistic regression models the probability of connection in terms of the above-mentioned variables as

$$\begin{array}{c} P\left(Y=1\right)=\frac{1}{1+e^{-(\beta_{0}+\beta_{p}\cdot X_{p}+\beta_{b}\cdot X_{b}+\beta_{s}\cdot X_{s}+\beta_{l}\cdot X_{l})}}, \end{array}$$ (8)

where the regression coefficients {*β*_*p*_, *β*_*b*_, *β*_*s*_, *β*_*l*_} are estimated from the empirical data. These can be interpreted as change in the logarithm of odds (i.e., $log(\frac{P}{1-P})$) that results from a change in the corresponding predictor variable by a single unit, with other variables kept unchanged. Therefore, the magnitude of the regression coefficients (determined by us using the mnrfit function in *MATLAB Release 2010b*) provides a measure of the relative contribution of each of the factors in determining the probability of connection. We have also explicitly ensured the absence of multicollinearity (i.e., correlations) among the predictor variables, as this would otherwise result in an inaccurate estimation of the regression coefficients. For this purpose, we have used Belsley collinearity diagnostics [65] as implemented in the collintest function in *MATLAB Release 2016b*.

### Statistics

*Two-sample Kolmogorov-Smirnov (KS) test* [95] has been used to compare between pairs of samples (e.g., the degrees of neurons belonging to different categories) to determine whether both of them are drawn from the same continuous distribution (null hypothesis) or if they belong to different distributions. For this purpose we have used the kstest2 function in *MATLAB Release 2010b*, with the value of the parameter *α* which determines threshold significance level set to 0.05.

*Kernel smoothened density function* [96] has been used to estimate the probability distribution functions of different quantities (e.g., distances between cell bodies of neurons). For this purpose we have used the ksdensity function in *MATLAB Release 2010b* with a Gaussian kernel.

### Code availability

The codes and associated data files used for the analysis reported here are accessible from https://github.com/anandpathak31/C_elegans_development.

## Supporting information

S1 Fig Birth cohort homophily is seen specifically for connections between neurons whose cell bodies are in close physical proximity. Frequency distributions of the mean birth time for all pairs that are connected via synapses (A-C) or gap-junctions (D-F). The distributions for the empirical network (shown in red) are compared with distributions obtained from surrogate ensembles of randomized networks (blue curve shows the average over 100 realizations, the dispersion being indicated by the shaded area). The latter are constructed from the empirical network by randomly rewiring the connections while keeping the total number of connections (degree) for each neuron, the spatial location of its cell body and its process length unchanged. In addition, to allow only physically possible connections between neurons, we have imposed process-length constraint which disallow linking two cells if the distance between their cell bodies is greater than the sum of their individual process lengths. The different panels correspond to connections between neurons whose cell bodies are separated by distance *d* which is short (*d* < *L*/3: A, D), medium (*L*/3 < *d* < 2*L*/3: B, E) or long (*d* > 2*L*/3: C, F) relative to the total body length of the worm *L*. As in Fig 4 in the main text, the trimodal nature of these distributions arise from three classes of connected neuronal pairs, viz., (i) where both cells are born early (i.e., in the embryonic stage), (ii) where one is born early while the other late (i.e., in the post-embryonic stage) and (iii) where both are born late. Birth cohort homophily is indicated when the peaks of the empirical frequency distribution, corresponding to connections between neurons that are either both born early or both born late, have significantly higher values than the randomized distribution (the latter corresponding to a null model where connections between cells can occur independent of the time of their birth). This is seen only in panels (A) and (D), i.e., for connections between neurons whose cell bodies are located relatively close to each other.
(TIF)

S2 Fig Spatial distribution of cell bodies of the neurons belonging to the somatic nervous system of *Caenorhabditis elegans*. (A) Projection of the physical locations of the neuronal cell bodies on the two-dimensional plane formed by the anterior-posterior (AP) axis (*x*, along the horizontal) and the ventral-dorsal axis (*y*, along the vertical). Cells having short, medium and long processes are indicated using different symbols. The animal is oriented such that its head is located near the left end and tail near the right end of the plane. (B-D) Probability distributions of the location of the cell bodies along the AP axis (*x*, measured in mm) for neurons having (B) short, (C) medium and (D) long processes. We note that the distributions for neurons having short and long processes, both have an approximately bimodal nature. It suggests that most cells of these two types are localized near either the head or the tail regions, while neurons with medium length processes are distributed across the body of the worm in a relatively more homogeneous manner.
(TIF)

S3 Fig Distribution of distances between cell bodies of synaptically connected pairs of neurons differs from that of all pairs. Comparison of the distributions of distances between synaptically connected pairs (blue) and all pairs (red) of neurons distinguished in terms of their respective process lengths (S: short, M: medium, L: long). The mean value for each of the distributions (distinguished by their color) is marked by broken lines. Note that for all pairs of process length categories (with the exception of LS and ML), the average distance for synaptically connected pairs is less than the average calculated over all pairs of neurons, consistent with the results shown in Supporting Information, S3 Table.
(TIF)

S4 Fig Distribution of distances between cell bodies of pairs of neurons connected by gap-junctions differs from that of all pairs. Comparison of the distributions of distances between pairs connected by gap-junctions (blue) and all pairs (red) of neurons distinguished in terms of their respective process lengths (S: short, M: medium, L: long). The mean value for each of the distributions (distinguished by their color) is marked by broken lines. Note that for all pairs of process length categories, the average distance for pairs connected by gap-junctions is less than the average calculated over all pairs of neurons, consistent with the results shown in Supporting Information, S3 Table.
(TIF)

S5 Fig Dependence of the probability of connection between two neurons on the physical distance between their cell bodies. (A) The variation of the probability of connection between two neurons by either synapse (squares) or gap-junction (circles) as a function of the physical distance between their cell bodies *d* (measured in mm). Linear fitting of the functions show a decay with *d* overall (thick broken lines), but the relation is much weaker compared to that seen between probability of synaptic connection between two cells and their lineage distance *l* [see Fig 2(D) in main text]. In particular, the correlation is diluted by the relatively high probability for synapses to form between neurons whose cell bodies are located at the opposite ends of the worm (corresponding to the peak around *x* = 1 mm). However, when we focus only on connections between cell bodies that are in close physical proximity (*d* < 0.4 mm), the dependence on *d* appears to be much more prominent (thin broken lines). This stronger correlation between connection probability and *d* at short distances is not necessarily an outcome of constraints imposed by the process lengths of the neurons. This is suggested by panel (B), where we focus exclusively on neurons with short processes. (B) The relation between connection probability between neurons, both of which have short processes, and the distance between their cell bodies, *d*, is seen to be not more prominent than that already seen for all neurons [in panel (A)].
(TIF)

S6 Fig A stochastic branching model for the lineage tree of cells involved in the development of the *C. elegans* somatic nervous system. (A) Comparison of the distribution of rung *R* occupied by each cell (progenitor cells of the neurons, as well as, differentiated neurons) in the lineage tree obtained empirically (broken curve) with that generated by the model (solid curve shows the mean computed over an ensemble of 10^3^ realizations, the dispersion being indicated by the shaded area). (B) Comparison of the distribution of lineage distance *l* between pairs of differentiated neurons of *C. elegans* (broken curve) with that obtained from the model (solid curve showing the mean computed over an ensemble of 10^3^ realizations, the dispersion being indicated by the shaded area). The high degree of overlap between the empirical and simulated distributions indicates that the stochastic branching model is a reasonably accurate description of the lineage tree of neurons. (C) The branching probabilities *P*1 (blue curve) and *P*2 (red curve) of a progenitor cell at each rung, estimated from the empirical lineage tree (by definition, *P*1 ≥ *P*2). Note that both of the branching probabilities show a prominent dip after rung 10. Guided by this, in the stochastic branching model, *P*1, *P*2 have been chosen to have a constant high value upto rung 10 (viz., *P*1 = 1, *P*2 = 0.85), after which both are decreased to a constant low value (viz., *P*1 = 0.25, *P*2 = 0.2). The inset shows a schematic of the stochastic branching model where a node, occurring at any rung, can branch (or not) based on the probabilities *P*1 and *P*2 which will result in any one of the following three possibilities: (i) proliferation occurs along both branches, (ii) only one branch appears (the other branch leading to either apoptosis or a non-neural cell fate), and, (iii) there is no branching so that we obtain a terminal node of the tree (i.e., the cell differentiates into a neuron).
(TIF)

S7 Fig Developmental chrono-dendrograms for the anterior (G1, top left), dorsal (G2, top right), lateral (G3, bottom left) and ventral (G4, bottom right) ganglia, showing that each comprises multiple localized clusters of neurons. Colored nodes represent neurons belonging to the specified ganglion while gray nodes show other neurons. Branching lines trace all cell divisions starting from the single cell zygote (located at the origin) and terminating at each differentiated neuron. The time and rung of each cell division is indicated by its position along the vertical and radial axis respectively. The entire time period is divided into four stages, viz., Embryo (indicated as E), L1, L2 and L3. A planar projection at the base of each cylinder shows the rung (concentric circles) of each progenitor cell and differentiated neuron.
(TIF)

S8 Fig Developmental chrono-dendrograms for the retrovesicular (G5, top left), posterolateral (G6, top right), preanal (G7, bottom left) and dorsorectal (G8, bottom right) ganglia, showing that each comprises multiple localized clusters of neurons. Colored nodes represent neurons belonging to the specified ganglion while grey nodes show other neurons. Branching lines trace all cell divisions starting from the single cell zygote (located at the origin) and terminating at each differentiated neuron. The time and rung of each cell division is indicated by its position along the vertical and radial axis respectively. The entire time period is divided into four stages, viz., Embryo (indicated as E), L1, L2 and L3. A planar projection at the base of each cylinder shows the rung (concentric circles) of each progenitor cell and differentiated neuron.
(TIF)

S9 Fig Developmental chrono-dendrograms for the lumbar ganglion (G9, left) and the ventral cord (G10, right), showing that each comprises multiple localized clusters of neurons. Colored nodes represent neurons belonging to the specified ganglion while grey nodes show other neurons. Branching lines trace all cell divisions starting from the single cell zygote (located at the origin) and terminating at each differentiated neuron. The time and rung of each cell division is indicated by its position along the vertical and radial axis respectively. The entire time period is divided into four stages, viz., Embryo (indicated as E), L1, L2 and L3. A planar projection at the base of each cylinder shows the rung (concentric circles) of each progenitor cell and differentiated neuron.
(TIF)

S10 Fig Connections between neurons born at different developmental epochs are over-represented when the cell bodies are far apart, suggesting the presence of active processes facilitating such links. Frequency distributions of the distance *d* between cell bodies of all neuronal pairs that are connected via synapses (A-D) or gap-junctions (E-G). The distributions for the empirical network (shown in red) are compared with distributions obtained from surrogate ensembles of randomized networks (blue curve shows the average over 100 realizations, the dispersion being indicated by the shaded area). The latter are constructed from the empirical network by randomly rewiring the connections while keeping the total number of connections (degree) for each neuron, the spatial location of its cell body and its process length unchanged. In addition, to allow only physically possible connections between neurons, we have imposed process-length constraint which disallow linking two cells if the distance between their cell bodies is greater than the sum of their individual process lengths. The different panels correspond to the situations where (A,E) both cells in a connected pair are born in the early developmental burst, (B,C,F) one is born early and the other is born late [in (B) it is the pre-synaptic neuron which is born early, while in (C) the post-synaptic neurons appears in the early developmental burst], and (D,G) both cells are born late. When two neurons are born in the same developmental epoch (either early or late), the empirical frequency distribution is seen to have significantly higher values than the randomized distribution at low *d* (seen in panels A and D, and even more prominently in panels E and G), indicating that neurons prefer to connect to other members of their birth cohort whose cell bodies are in close proximity. This is particularly evident for neurons born in the late developmental epoch. Note that this result complements the earlier observation that birth cohort homophily is seen specifically for neurons whose cell bodies are located relatively close to each other (Supporting Information, S1 Fig). More intriguingly, connections between neurons whose cell bodies lie far apart are seen to occur more frequently than expected by chance when the pre-synaptic neuron is born early and the post-synaptic neuron is born late (see panel B). A similar phenomenon is also seen in the case of early- and late-born neurons connected by gap junctions (see panel F). These results suggest the presence of an active process forming connections between neurons born in different epochs.
(TIF)

S11 Fig Absence of segregated clusters in the developmental chrono-dendrograms for neurons having similar process lengths (viz., short, medium and long) suggest that process length is not exclusively determined by lineage. Colored nodes represent neurons having a specified process length, viz., short in (A), medium in (B) and long in (C), while grey nodes show other neurons. Branching lines trace all cell divisions starting from the single cell zygote (located at the origin) and terminating at each differentiated neuron. The time and rung of each cell division is indicated by its position along the vertical and radial axis respectively. The entire time period is divided into four stages, viz., Embryo (indicated as E), L1, L2 and L3. A planar projection at the base of each cylinder shows the rung (concentric circles) of each progenitor cell and differentiated neuron.
(TIF)

S12 FigPhysical proximity alone cannot explain the high degree of overlap between the cells that each member of a bilaterally symmetric pair of neurons connect to.Complementary cumulative probability distributions (CCDF) of the overlap between the sets $N_{G}\left(i\right)$, $N_{G}\left(j\right)$ of the neighbors (defined for a network *G*) of neurons *N*_*i*_ and *N*_*j*_, where the indices *i* and *j* can run over (i) all pairs of neurons (dash-dotted curves), (ii) only bilaterally symmetric pairs (solid curves) or (iii) pairs whose cell bodies are spatially adjacent to each other (*d* < 0.05 mm, broken curves). The overlap is measured in terms of the Jaccard index *J*, defined for the pair *i*, *j* as $J\left(i,j\right)=\left[N_{G}\left(i\right)\cap N_{G}\left(j\right)\right]/\left[N_{G}\left(i\right)\cup N_{G}\left(j\right)\right]$, where ∩ and ∪ refers to intersection and union of two sets, respectively. The different panels correspond to different networks *G* used to define neighbors for a neuron, viz., (A) pre-synaptic neighbors, i.e., cells from which the neuron receives a synaptic connection, (B) post-synaptic neighbors, i.e., cells to which the neuron sends a synaptic connection, and (C) gap-junctional neighbors, i.e., cells to which a neuron is coupled via a gap junction. We note that the overlaps between the neighborhoods (for all three types of network neighbors considered here) of bilaterally symmetric neurons are consistently higher than that of all pairs of neurons, as well as, of pairs whose cell bodies are in close physical proximity. Thus, bilaterally symmetric neurons share neighbors to a much greater extent than that expected by their cell bodies being located close to each other. (D-F) The Jaccard index matrices *J* showing overlaps between the neighbors for every pair of neurons *N*_*i*_, *N*_*j*_ when the network neighborhood defined is that of (D) pre-synaptic partners, (E) post-synaptic partners and (F) gap-junctional partners. The large overlap between neighbors of bilaterally symmetric neurons is indicated by the occurrence of bands of brightly colored entries along the diagonal (note that bilaterally symmetric neurons are always located on adjacent rows/columns).(TIF)

S13 Fig Spatial distribution of the cell bodies of sensory, inter and motor neurons of the somatic nervous system of *Caenorhabditis elegans*. Projections of the physical locations of the neuronal cell bodies, distinguished according to functional type (sensory: circles, inter: triangles and motor: squares) and whether they appear in the early (unfilled symbols) or late (filled symbols) developmental epochs, on the two-dimensional plane formed by the anterior-posterior (AP) axis (*x*, along the horizontal) and the dorsal-ventral axis (*y*, along the vertical). Top panel shows the entire worm, with its body oriented such that the head is located near the left end and tail near the right end of the plane. The bottom panel shows a magnified view of the region near the head (bounded by broken lines in the top panel). We note that almost all cells in this region appear at the embryonic stage, during the early burst of development. By contrast, the ventral cord predominantly comprises neurons that appear in the post-embryonic stage (see top panel).
(TIF)

S14 Fig The number of synaptic connections of a neuron is influenced by its functional criticality, as well as, the developmental epoch in which it appeared. (A-C) Scatter plots indicating the relation between the time of appearance of a neuron and its number of (A) incoming synaptic connections from other cells (synaptic in-degree), (B) outgoing synaptic connections to other cells (synaptic out-degree) and (C) gap junctions with other cells (gap-junctional degree). Filled circles represent neurons belonging to any of nine previously identified functional circuits (see Fig 8 in main text) while unfilled circles show other neurons. (D-F) Probability distributions of different types of connections for neurons categorized in terms of those which are functionally critical, i.e., belong to a functional circuit (red), or not (blue), and whether they appear in the early (solid curve) or late (broken curve) developmental epochs. The different panels correspond to (D) synaptic in-degree, (E) synaptic out-degree and (F) gap-junctional degree. Synaptic in-degree for functionally critical, early-born neurons is seen to have a heavy-tailed distribution which is significantly different from that of the other types of neurons (in terms of a 2-sample Kolmogorov-Smirnov test at 5% level of significance). For the case of synaptic out-degree, however, the distributions for functionally critical neurons that are born at different epochs are statistically indistinguishable. However, for other neurons, the distribution of those that are born in the later, post-embryonic developmental burst are distinct from those that are born early (demonstrated by a 2-sample Kolmogorov-Smirnov test at 5% level of significance). This statistically significant difference between the outgoing connections of early and late-born neurons could arise from the former neurons being present for a much longer period during which they can send out synapses. Distributions of gap junctional connections for all categories of neuron appear to be statistically indistinguishable, suggesting that gap junction formation is relatively unaffected by the functional criticality or time of appearance of the neurons.
(TIF)

S15 Fig Comparison of the *Caenorhabditis elegans* connectome data obtained from Cook *et al*. [90] and Varshney *et al*. [11]. Difference between the adjacency matrices corresponding to the two connectivity data-sets are shown for synapses (left) and gap-junctions (right). Black-colored entries represent connections common to both datasets, while red- and blue-colored entries denote connections that are found exclusively in the Cook and Varshney datasets, respectively. The columns and rows are arranged according to the spatial ordering of the neuronal cell bodies along the head-tail axis. The horizontal and vertical panels for the synapse matrix shows the difference in the out-degree and in-degree, respectively, of each neuron across the two data-sets. For the gap-junction matrix, the horizontal panel shows the change in degree for each neuron across the data-sets. We note that the two data-sets differ substantially, especially in terms of a large number of additional gap-junctions near the tail region that appear in the database of Cook *et al*.
(TIF)

S16 Fig Comparison of the *Caenorhabditis elegans* connectome data obtained from Varshney *et al*. [11] and Haspel *et al*. [89]. Difference between the adjacency matrices corresponding to the two connectivity data-sets are shown for synapses (left) and gap-junctions (right). Black-colored entries represent connections common to both datasets, while red- and blue-colored entries denote connections that are found exclusively in the Haspel and Varshney datasets, respectively. The columns and rows are arranged according to the spatial ordering of the neuronal cell bodies along the head-tail axis. The horizontal and vertical panels for the synapse matrix shows the difference in the out-degree and in-degree, respectively, of each neuron across the two data-sets. For the gap-junction matrix, the horizontal panel shows the change in degree for each neuron across the data-sets. We note that the two data-sets differ relatively little.
(TIF)

S1 Table Process length homophily among neurons segregated into groups comprising cells with long, medium and short processes. The extent of homophily is quantified by the modularity measure *Q* computed over the different classes of neurons (which are considered to be the communities or modules for the purpose of calculation of *Q*). The empirical *Q* values are compared with those calculated from two different types of randomized surrogate ensembles. Members of one of these ensembles are constructed by randomly shuffling the process length categories of the empirical network keeping the network connections invariant, while those belonging to the other ensemble are obtained by degree preserved randomization of the empirical network with the cell positions and process lengths kept unchanged (top and center, respectively). Note that the *Q* values are significantly higher than that expected by chance (as seen for the surrogate ensembles) for the entire network, as well as, individually within each of the different process length categories, suggesting process length homophily in both synaptic and gap-junction connections between neurons. (Bottom) Note that significantly higher values of class-specific *Q* (compared to the randomized surrogate) occur only when we consider pairs of neurons belonging to the same class, further underlining the process length homophily.
(XLS)

S2 Table Birth cohort homophily among neurons segregated into groups comprising cells differentiating before and after hatching (early and late-born, respectively). The extent of homophily is quantified by modularity *Q* computed over the different classes of neurons (which are considered to be the communities or modules for the purpose of calculating *Q*). (Top) The empirical values are compared with those calculated from the corresponding surrogate ensemble obtained by degree preserved randomization with the cell positions and process lengths of neurons kept unchanged. Note that the *Q* values are significantly higher than that expected by chance (as seen from the surrogate ensemble) for the entire network, as well as, individually for the early-born and late-born cohorts. This is indicative of birth cohort homophily for synaptic, as well as, gap-junctional connections between neurons. (Bottom) The *z*-scores for class-specific *Q* values computed with respect to the randomized surrogate ensemble. Significantly higher values of *Q* occur only when we consider pairs of neurons belonging to the same category (early- or late-born) and not for pairs where the constituent neurons belong to different categories, further underlining the homophily.
(XLS)

S3 Table For synaptically connected neurons, process length of the pre-synaptic neuron primarily decides the average distance between the cell bodies. Statistically significant deviation (measured in terms of *z*-score) between the average distance 〈*d*〉 of cell bodies in pairs of connected neurons (having short, medium or long processes) and the average distance 〈*D*〉 between any pair of neurons randomly sampled from the same process length categories. The latter average is calculated over a set having the same number of pairs as for the set of connected pairs. Note that except for two cases (pre-synaptic long process to post-synaptic short process and pre-synaptic medium process to post-synaptic long process, shown in bold font), connections between cells in all other process length categories tend to be much shorter than that expected by chance, as indicated by *z* < 0.
(XLS)

S4 Table Neurons belonging to the somatic nervous system of *C. elegans* segregated into those which are born in the early (embryonic) and those born in the late (post-embryonic) developmental bursts. The respective lineage information and functional description are also provided. Motor neurons are highlighted. We note that motor neurons that appear early mostly innervate dorsal muscles, whereas, motor neurons that appear late primarily innervate ventral muscles. All information shown here is obtained from WormAtlas [81].
(XLS)

S5 Table Time of appearance of neurons in the course of development of the *C. elegans* nervous system. The data has been manually transcribed from lineage charts provided in references [16, 17].
(XLS)

S6 Table Classification of *C. elegans* neurons according to their role in the mesoscopic organization. (XLS)

S7 Table The neuronal composition of different functional circuits in the *C. elegans* somatic nervous system. (XLS)
