# Supplementary figures and images for: Developmental trajectory of Caenorhabditis elegans nervous system governs its structural organization
Source: PLoS Comput Biol. 2020 Jan 2;16(1):e1007602. doi: 10.1371/journal.pcbi.1007602 (PMC6959611; doi:10.1371/journal.pcbi.1007602)

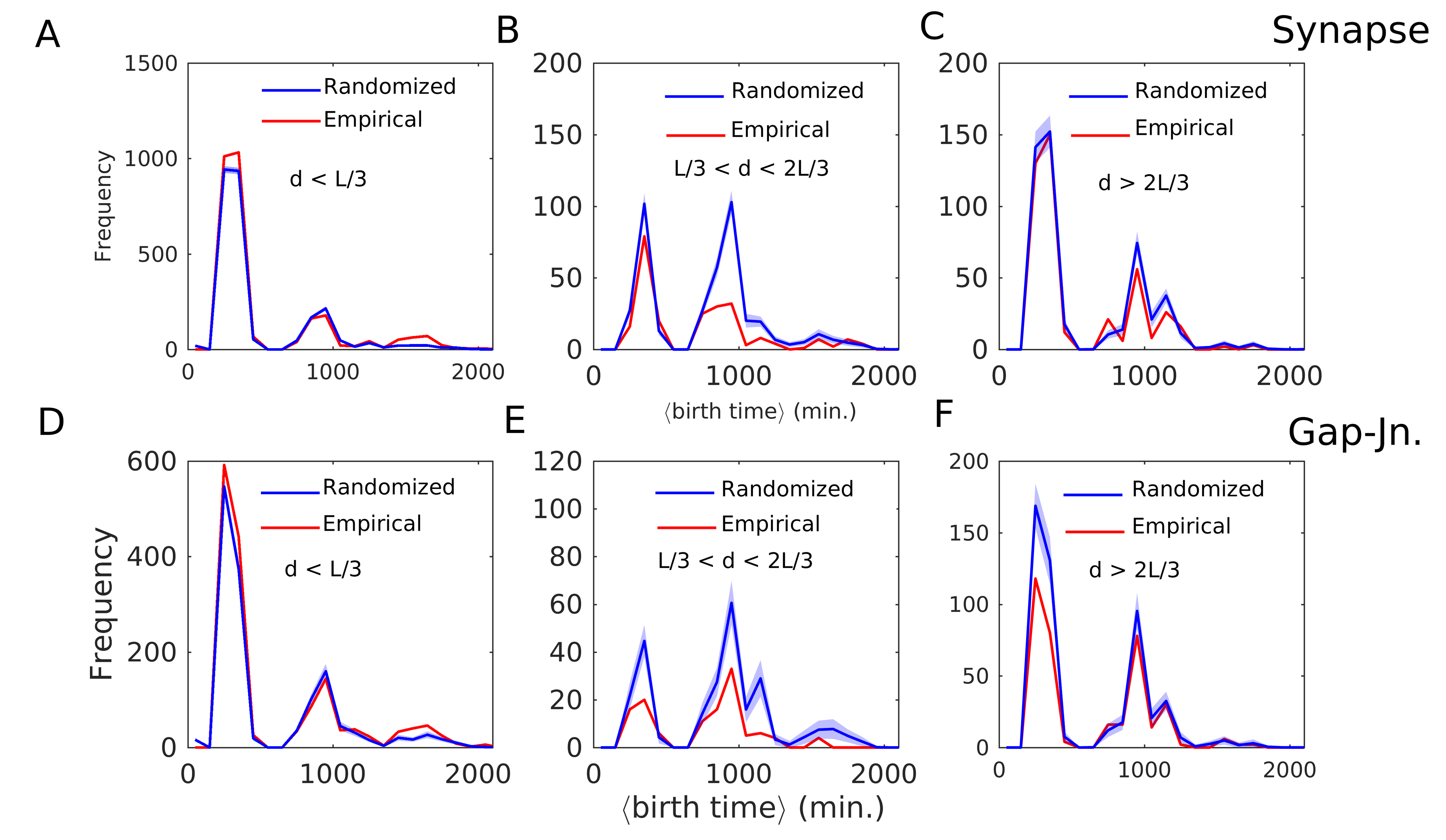

Supplement: S1 Fig — Frequency distributions of the mean birth time for all pairs that are connected via synapses (A-C) or gap-junctions (D-F). The distributions for the empirical network (shown in red) are compared with distributions obtained from surrogate ensembles of randomized networks (blue curve shows the average over 100 realizations, the dispersion being indicated by the shaded area). The latter are constructed from the empirical network by randomly rewiring the connections while keeping the total number of connections (degree) for each neuron, the spatial location of its cell body and its process length unchanged. In addition, to allow only physically possible connections between neurons, we have imposed process-length constraint which disallow linking two cells if the distance between their cell bodies is greater than the sum of their individual process lengths. The different panels correspond to connections between neurons whose cell bodies are separated by distance d which is short (d < L/3: A, D), medium (L/3 < d < 2L/3: B, E) or long (d > 2L/3: C, F) relative to the total body length of the worm L. As in Fig 4 in the main text, the trimodal nature of these distributions arise from three classes of connected neuronal pairs, viz., (i) where both cells are born early (i.e., in the embryonic stage), (ii) where one is born early while the other late (i.e., in the post-embryonic stage) and (iii) where both are born late. Birth cohort homophily is indicated when the peaks of the empirical frequency distribution, corresponding to connections between neurons that are either both born early or both born late, have significantly higher values than the randomized distribution (the latter corresponding to a null model where connections between cells can occur independent of the time of their birth). This is seen only in panels (A) and (D), i.e., for connections between neurons whose cell bodies are located relatively close to each other. (TIF) [file pcbi.1007602.s001.tif]

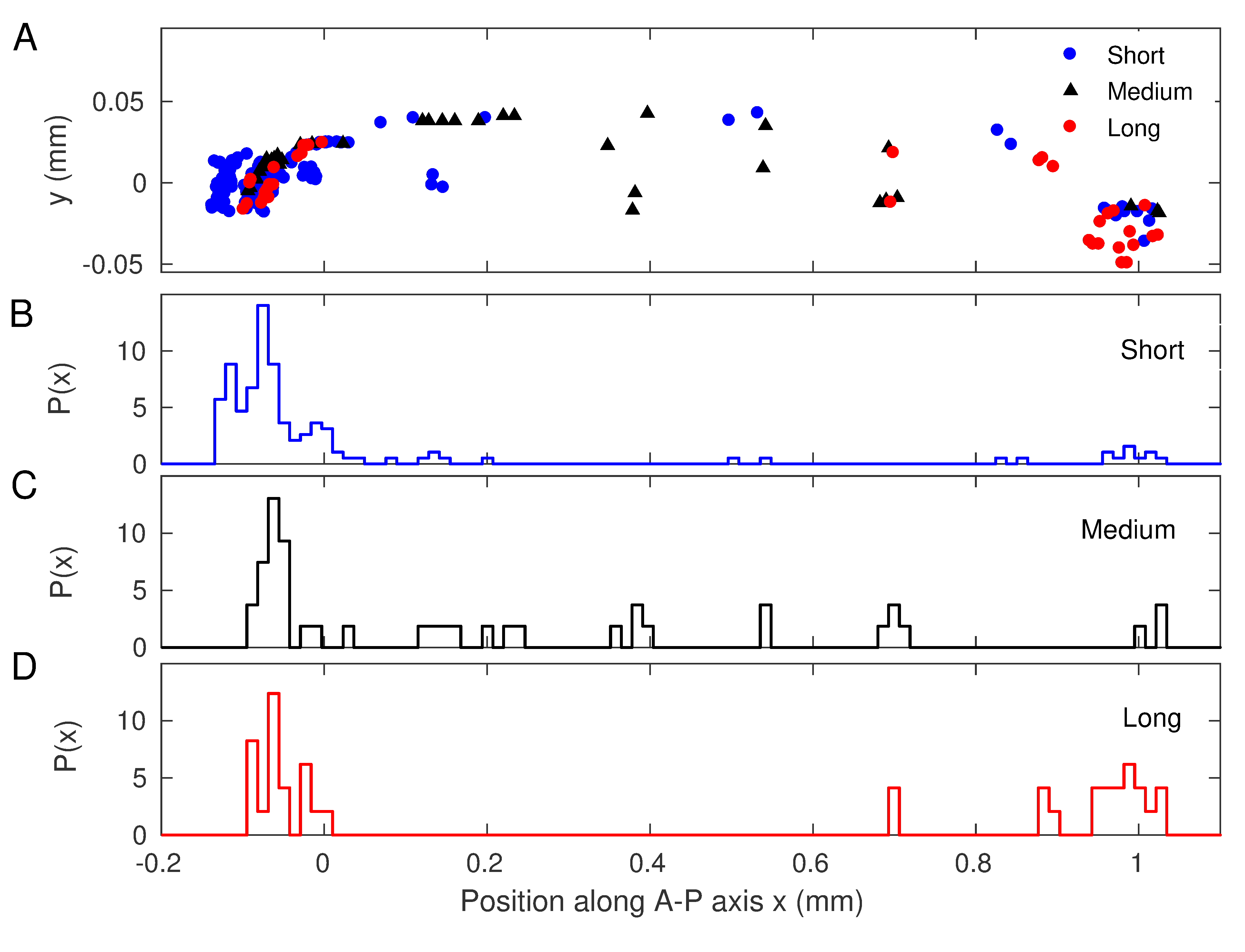

Supplement: S2 Fig — (A) Projection of the physical locations of the neuronal cell bodies on the two-dimensional plane formed by the anterior-posterior (AP) axis (x, along the horizontal) and the ventral-dorsal axis (y, along the vertical). Cells having short, medium and long processes are indicated using different symbols. The animal is oriented such that its head is located near the left end and tail near the right end of the plane. (B-D) Probability distributions of the location of the cell bodies along the AP axis (x, measured in mm) for neurons having (B) short, (C) medium and (D) long processes. We note that the distributions for neurons having short and long processes, both have an approximately bimodal nature. It suggests that most cells of these two types are localized near either the head or the tail regions, while neurons with medium length processes are distributed across the body of the worm in a relatively more homogeneous manner. (TIF) [file pcbi.1007602.s002.tif]

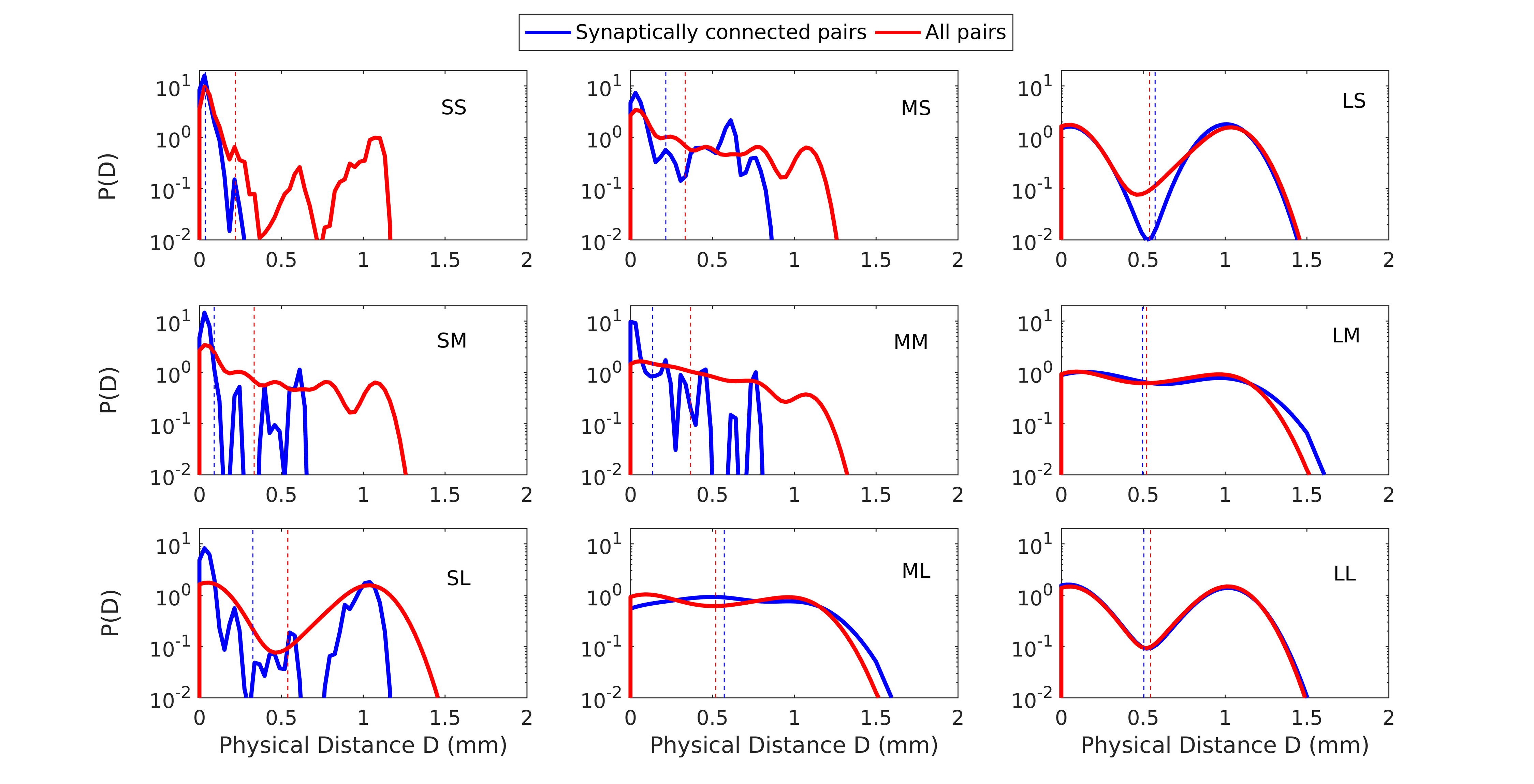

Supplement: S3 Fig — Comparison of the distributions of distances between synaptically connected pairs (blue) and all pairs (red) of neurons distinguished in terms of their respective process lengths (S: short, M: medium, L: long). The mean value for each of the distributions (distinguished by their color) is marked by broken lines. Note that for all pairs of process length categories (with the exception of LS and ML), the average distance for synaptically connected pairs is less than the average calculated over all pairs of neurons, consistent with the results shown in Supporting Information, S3 Table. (TIF) [file pcbi.1007602.s003.tif]

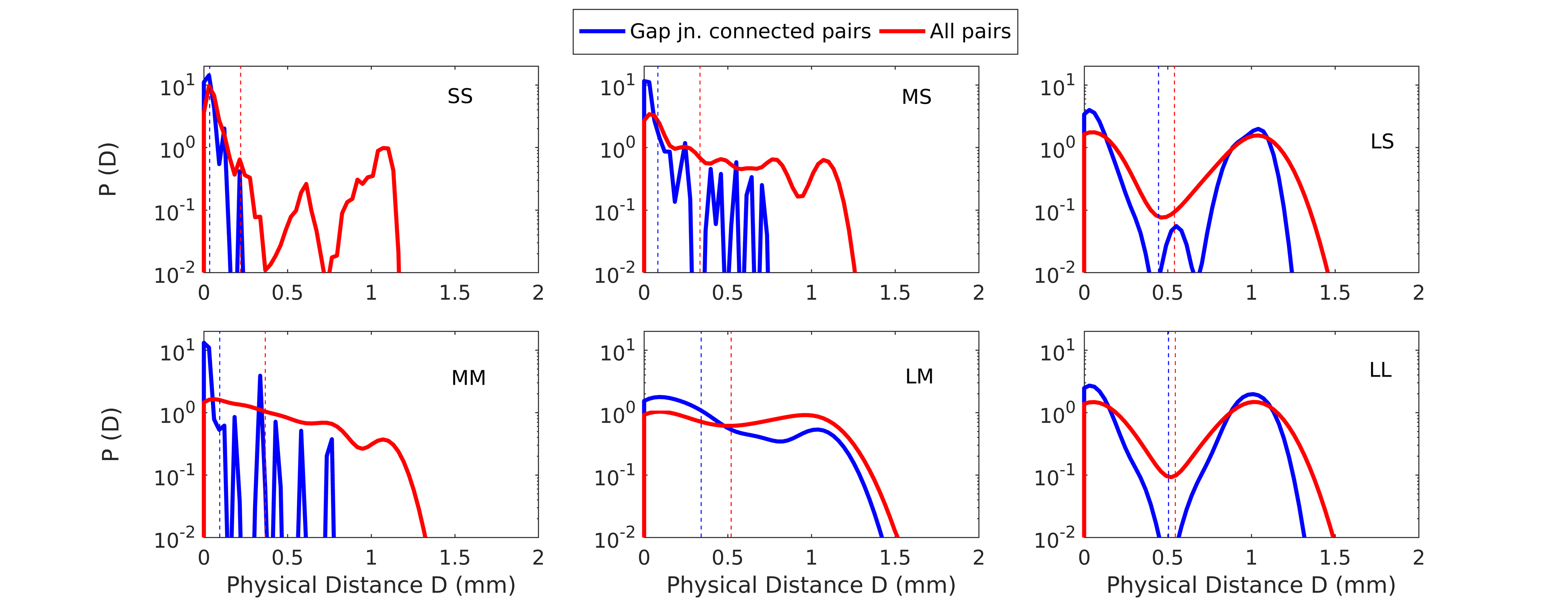

Supplement: S4 Fig — Comparison of the distributions of distances between pairs connected by gap-junctions (blue) and all pairs (red) of neurons distinguished in terms of their respective process lengths (S: short, M: medium, L: long). The mean value for each of the distributions (distinguished by their color) is marked by broken lines. Note that for all pairs of process length categories, the average distance for pairs connected by gap-junctions is less than the average calculated over all pairs of neurons, consistent with the results shown in Supporting Information, S3 Table. (TIF) [file pcbi.1007602.s004.tif]

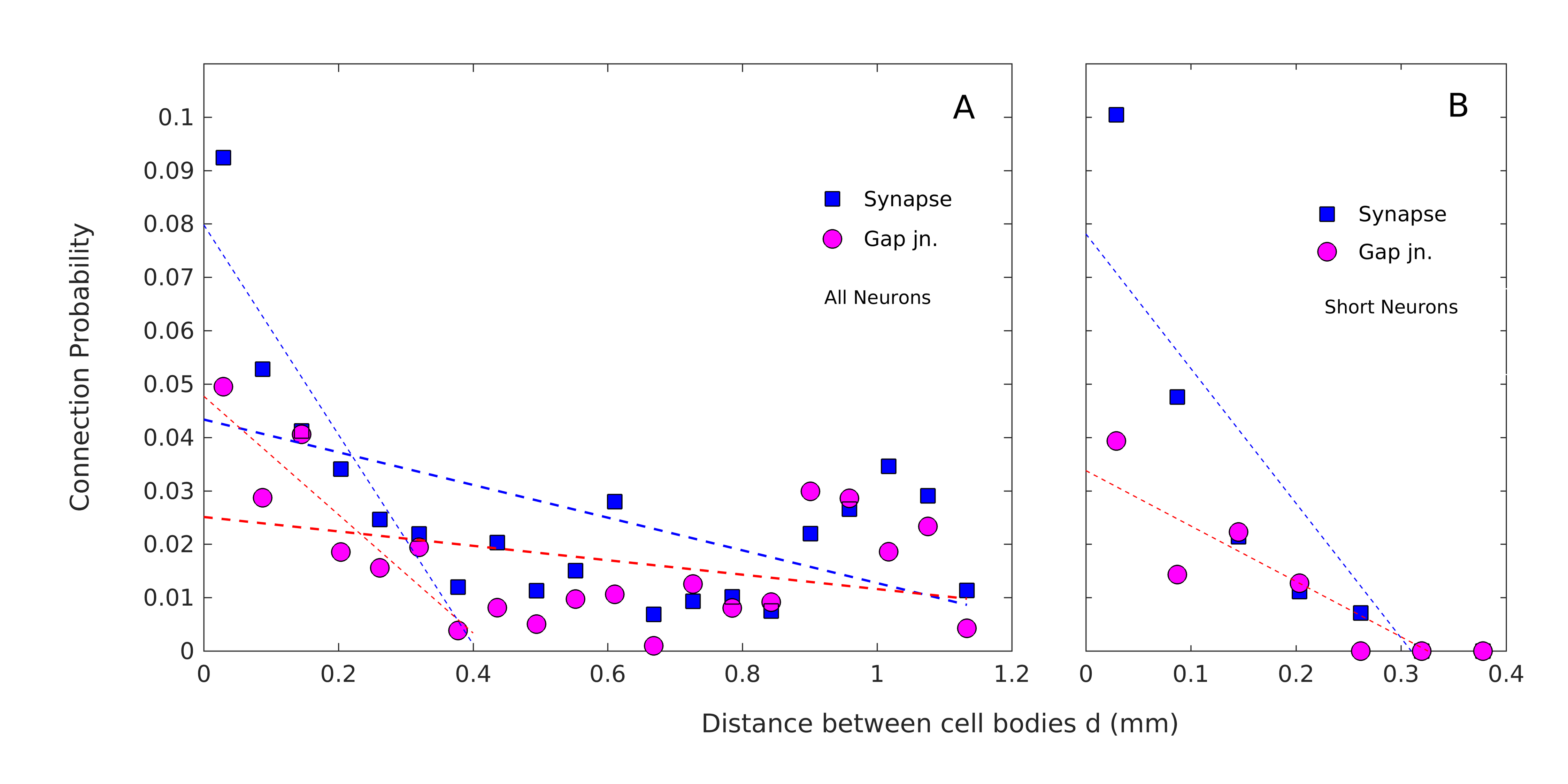

Supplement: S5 Fig — (A) The variation of the probability of connection between two neurons by either synapse (squares) or gap-junction (circles) as a function of the physical distance between their cell bodies d (measured in mm). Linear fitting of the functions show a decay with d overall (thick broken lines), but the relation is much weaker compared to that seen between probability of synaptic connection between two cells and their lineage distance l [see Fig 2(D) in main text]. In particular, the correlation is diluted by the relatively high probability for synapses to form between neurons whose cell bodies are located at the opposite ends of the worm (corresponding to the peak around x = 1 mm). However, when we focus only on connections between cell bodies that are in close physical proximity (d < 0.4 mm), the dependence on d appears to be much more prominent (thin broken lines). This stronger correlation between connection probability and d at short distances is not necessarily an outcome of constraints imposed by the process lengths of the neurons. This is suggested by panel (B), where we focus exclusively on neurons with short processes. (B) The relation between connection probability between neurons, both of which have short processes, and the distance between their cell bodies, d, is seen to be not more prominent than that already seen for all neurons [in panel (A)]. (TIF) [file pcbi.1007602.s005.tif]

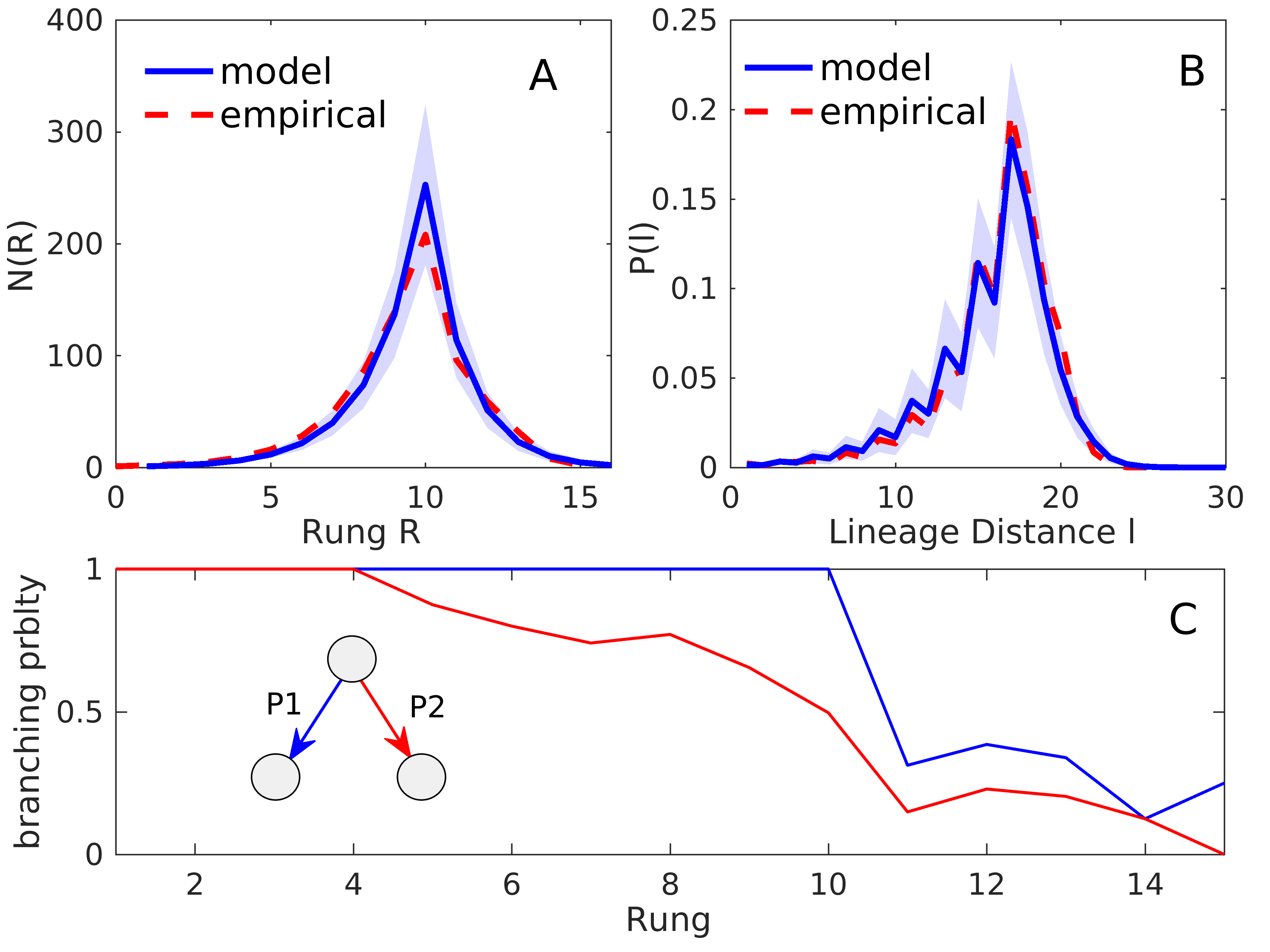

Supplement: S6 Fig — (A) Comparison of the distribution of rung R occupied by each cell (progenitor cells of the neurons, as well as, differentiated neurons) in the lineage tree obtained empirically (broken curve) with that generated by the model (solid curve shows the mean computed over an ensemble of 103 realizations, the dispersion being indicated by the shaded area). (B) Comparison of the distribution of lineage distance l between pairs of differentiated neurons of C. elegans (broken curve) with that obtained from the model (solid curve showing the mean computed over an ensemble of 103 realizations, the dispersion being indicated by the shaded area). The high degree of overlap between the empirical and simulated distributions indicates that the stochastic branching model is a reasonably accurate description of the lineage tree of neurons. (C) The branching probabilities P1 (blue curve) and P2 (red curve) of a progenitor cell at each rung, estimated from the empirical lineage tree (by definition, P1 ≥ P2). Note that both of the branching probabilities show a prominent dip after rung 10. Guided by this, in the stochastic branching model, P1, P2 have been chosen to have a constant high value upto rung 10 (viz., P1 = 1, P2 = 0.85), after which both are decreased to a constant low value (viz., P1 = 0.25, P2 = 0.2). The inset shows a schematic of the stochastic branching model where a node, occurring at any rung, can branch (or not) based on the probabilities P1 and P2 which will result in any one of the following three possibilities: (i) proliferation occurs along both branches, (ii) only one branch appears (the other branch leading to either apoptosis or a non-neural cell fate), and, (iii) there is no branching so that we obtain a terminal node of the tree (i.e., the cell differentiates into a neuron). (TIF) [file pcbi.1007602.s006.tif]

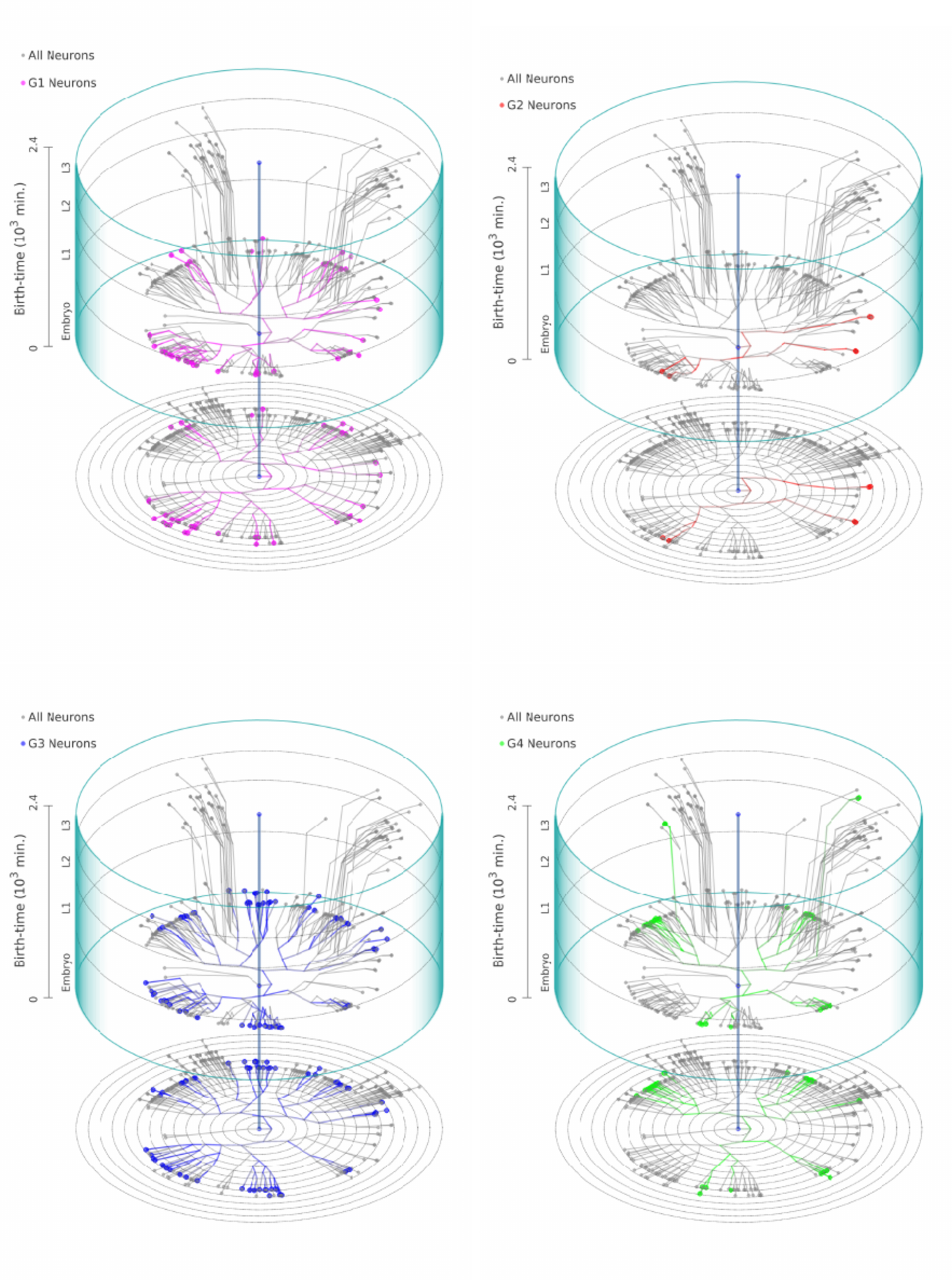

Supplement: S7 Fig — Colored nodes represent neurons belonging to the specified ganglion while gray nodes show other neurons. Branching lines trace all cell divisions starting from the single cell zygote (located at the origin) and terminating at each differentiated neuron. The time and rung of each cell division is indicated by its position along the vertical and radial axis respectively. The entire time period is divided into four stages, viz., Embryo (indicated as E), L1, L2 and L3. A planar projection at the base of each cylinder shows the rung (concentric circles) of each progenitor cell and differentiated neuron. (TIF) [file pcbi.1007602.s007.tif]

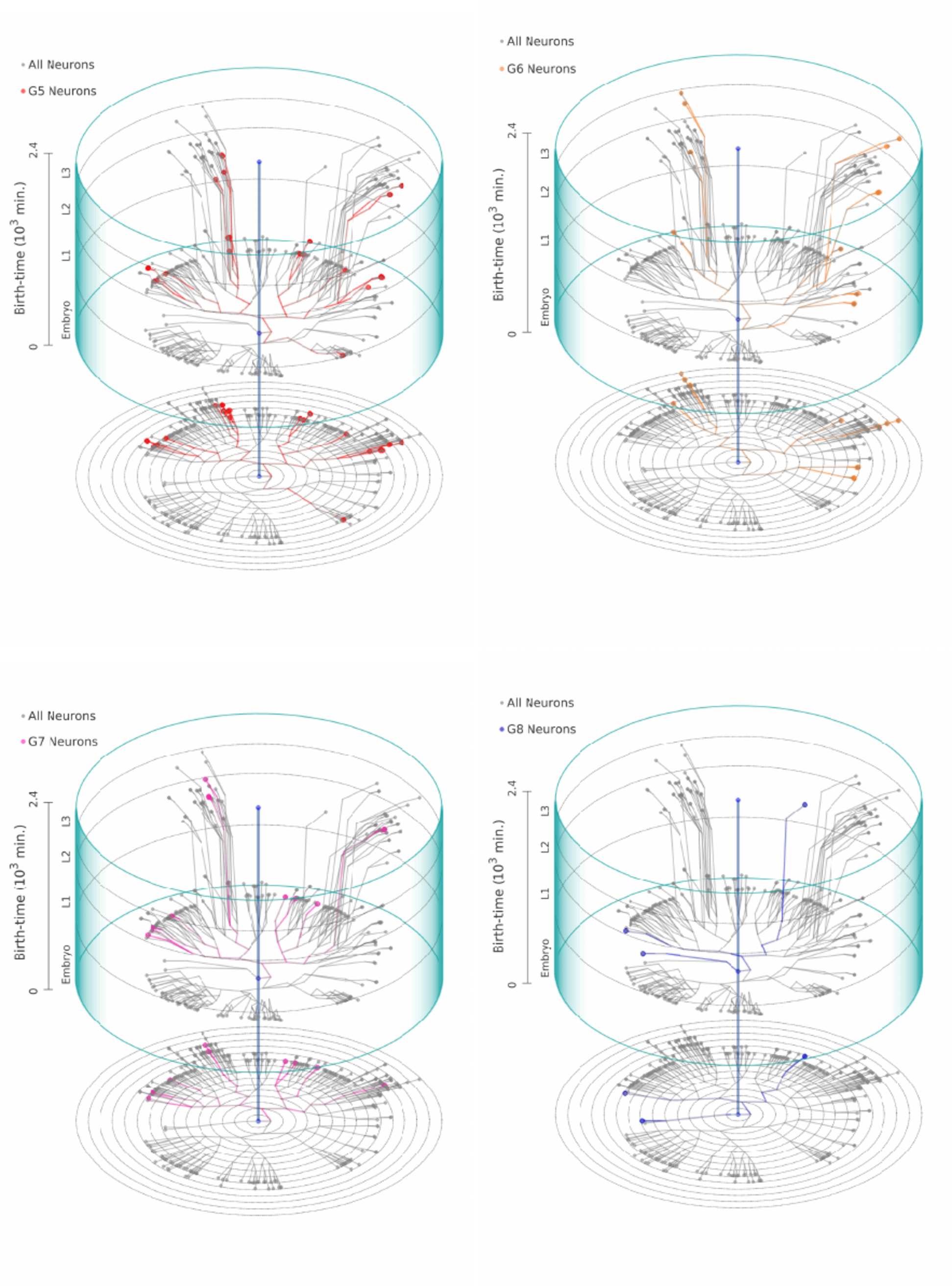

Supplement: S8 Fig — Colored nodes represent neurons belonging to the specified ganglion while grey nodes show other neurons. Branching lines trace all cell divisions starting from the single cell zygote (located at the origin) and terminating at each differentiated neuron. The time and rung of each cell division is indicated by its position along the vertical and radial axis respectively. The entire time period is divided into four stages, viz., Embryo (indicated as E), L1, L2 and L3. A planar projection at the base of each cylinder shows the rung (concentric circles) of each progenitor cell and differentiated neuron. (TIF) [file pcbi.1007602.s008.tif]

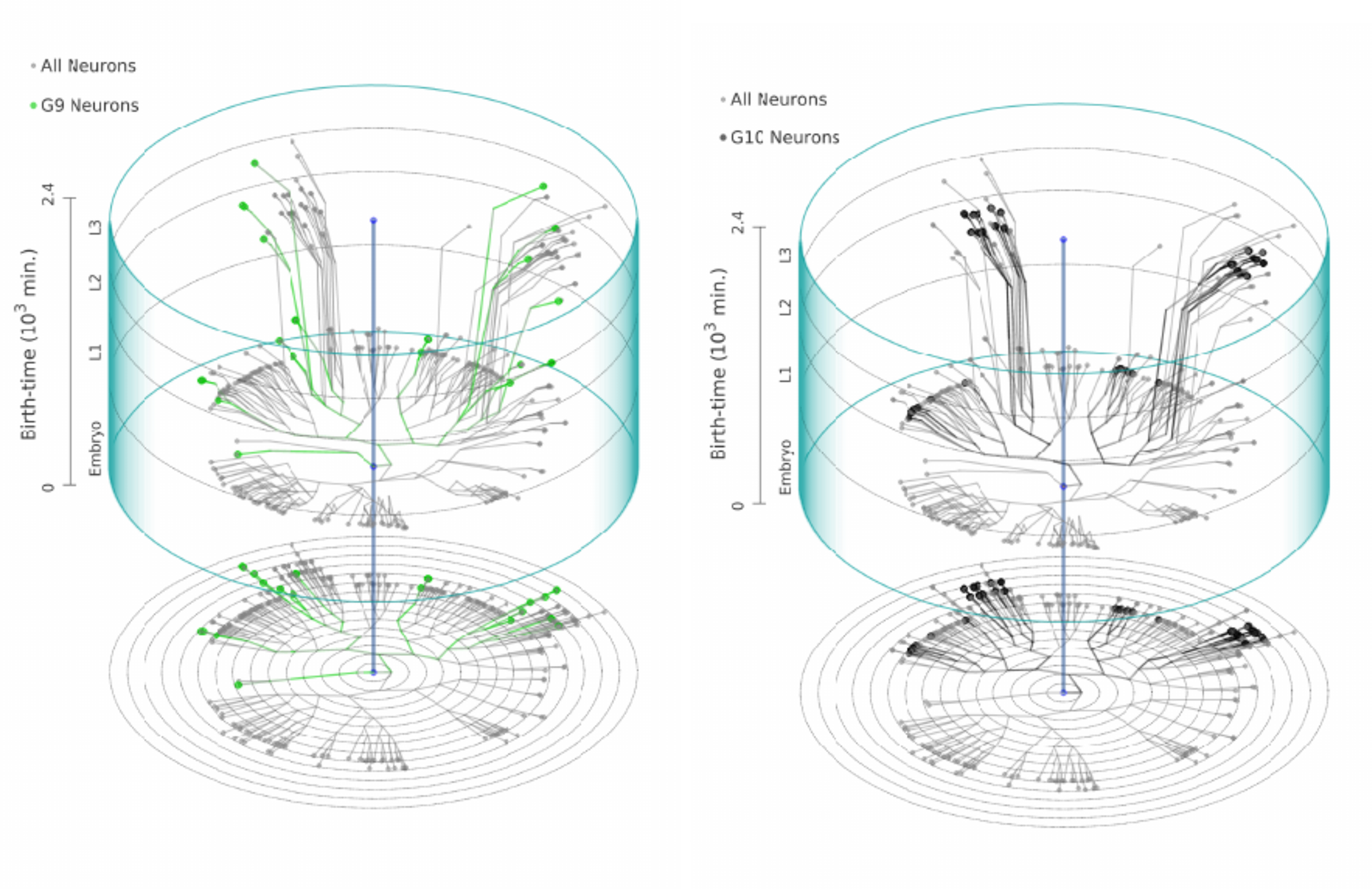

Supplement: S9 Fig — Colored nodes represent neurons belonging to the specified ganglion while grey nodes show other neurons. Branching lines trace all cell divisions starting from the single cell zygote (located at the origin) and terminating at each differentiated neuron. The time and rung of each cell division is indicated by its position along the vertical and radial axis respectively. The entire time period is divided into four stages, viz., Embryo (indicated as E), L1, L2 and L3. A planar projection at the base of each cylinder shows the rung (concentric circles) of each progenitor cell and differentiated neuron. (TIF) [file pcbi.1007602.s009.tif]

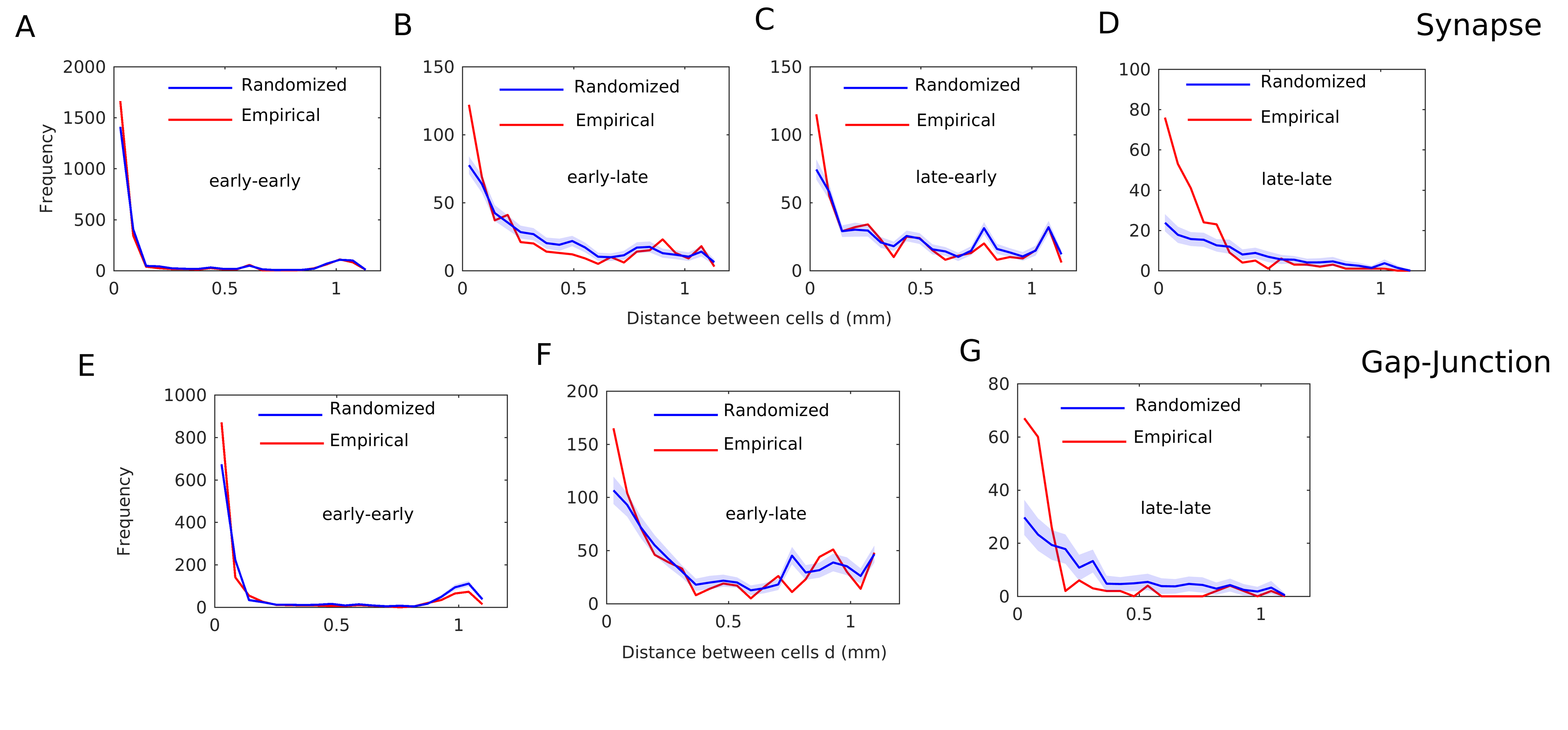

Supplement: S10 Fig — Frequency distributions of the distance d between cell bodies of all neuronal pairs that are connected via synapses (A-D) or gap-junctions (E-G). The distributions for the empirical network (shown in red) are compared with distributions obtained from surrogate ensembles of randomized networks (blue curve shows the average over 100 realizations, the dispersion being indicated by the shaded area). The latter are constructed from the empirical network by randomly rewiring the connections while keeping the total number of connections (degree) for each neuron, the spatial location of its cell body and its process length unchanged. In addition, to allow only physically possible connections between neurons, we have imposed process-length constraint which disallow linking two cells if the distance between their cell bodies is greater than the sum of their individual process lengths. The different panels correspond to the situations where (A,E) both cells in a connected pair are born in the early developmental burst, (B,C,F) one is born early and the other is born late [in (B) it is the pre-synaptic neuron which is born early, while in (C) the post-synaptic neurons appears in the early developmental burst], and (D,G) both cells are born late. When two neurons are born in the same developmental epoch (either early or late), the empirical frequency distribution is seen to have significantly higher values than the randomized distribution at low d (seen in panels A and D, and even more prominently in panels E and G), indicating that neurons prefer to connect to other members of their birth cohort whose cell bodies are in close proximity. This is particularly evident for neurons born in the late developmental epoch. Note that this result complements the earlier observation that birth cohort homophily is seen specifically for neurons whose cell bodies are located relatively close to each other (Supporting Information, S1 Fig). More intriguingly, connections between neurons whose c [file pcbi.1007602.s010.tif]

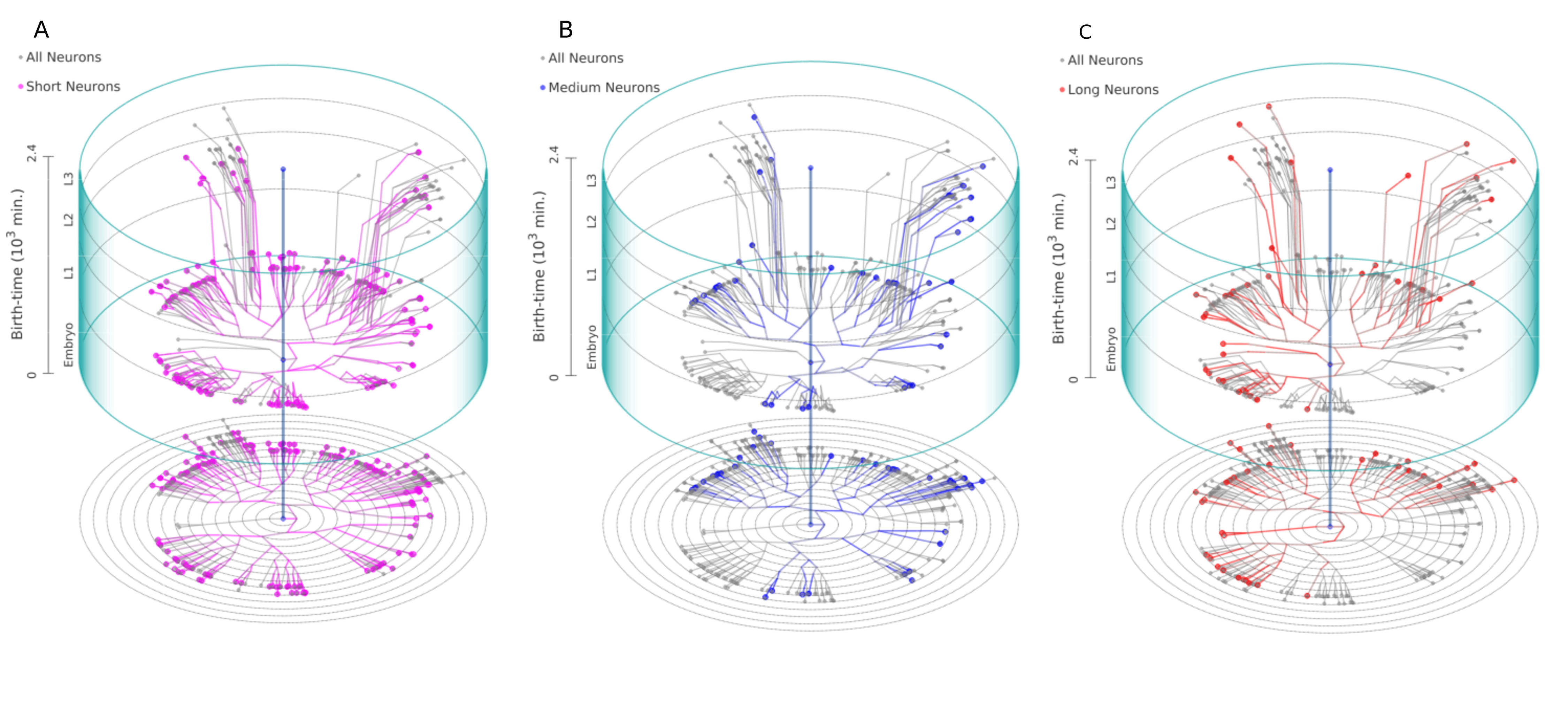

Supplement: S11 Fig — Colored nodes represent neurons having a specified process length, viz., short in (A), medium in (B) and long in (C), while grey nodes show other neurons. Branching lines trace all cell divisions starting from the single cell zygote (located at the origin) and terminating at each differentiated neuron. The time and rung of each cell division is indicated by its position along the vertical and radial axis respectively. The entire time period is divided into four stages, viz., Embryo (indicated as E), L1, L2 and L3. A planar projection at the base of each cylinder shows the rung (concentric circles) of each progenitor cell and differentiated neuron. (TIF) [file pcbi.1007602.s011.tif]

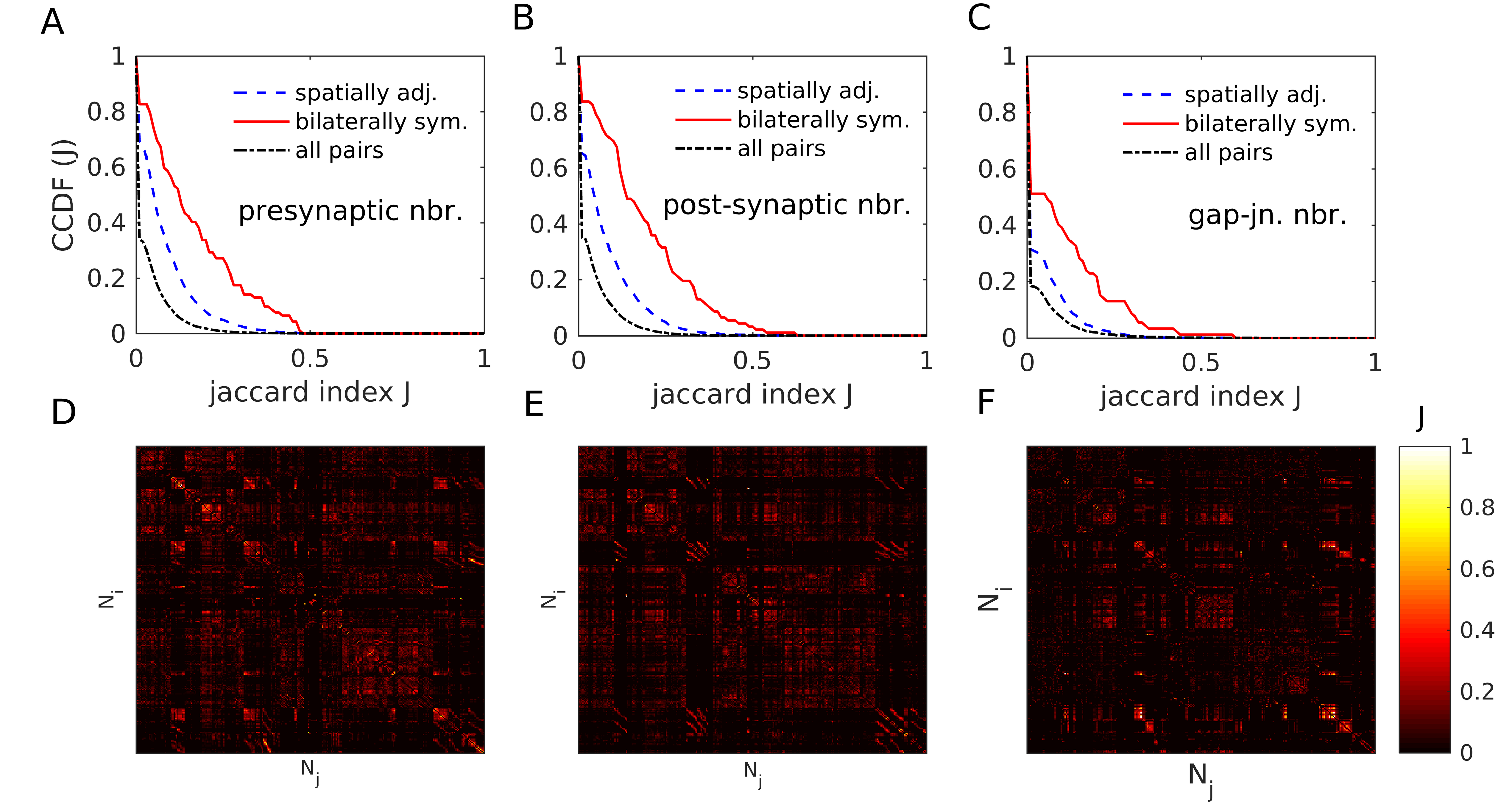

Supplement: S12 Fig — Complementary cumulative probability distributions (CCDF) of the overlap between the sets NG(i), NG(j) of the neighbors (defined for a network G) of neurons Ni and Nj, where the indices i and j can run over (i) all pairs of neurons (dash-dotted curves), (ii) only bilaterally symmetric pairs (solid curves) or (iii) pairs whose cell bodies are spatially adjacent to each other (d < 0.05 mm, broken curves). The overlap is measured in terms of the Jaccard index J, defined for the pair i, j as J(i,j)=[NG(i)∩NG(j)]/[NG(i)∪NG(j)], where ∩ and ∪ refers to intersection and union of two sets, respectively. The different panels correspond to different networks G used to define neighbors for a neuron, viz., (A) pre-synaptic neighbors, i.e., cells from which the neuron receives a synaptic connection, (B) post-synaptic neighbors, i.e., cells to which the neuron sends a synaptic connection, and (C) gap-junctional neighbors, i.e., cells to which a neuron is coupled via a gap junction. We note that the overlaps between the neighborhoods (for all three types of network neighbors considered here) of bilaterally symmetric neurons are consistently higher than that of all pairs of neurons, as well as, of pairs whose cell bodies are in close physical proximity. Thus, bilaterally symmetric neurons share neighbors to a much greater extent than that expected by their cell bodies being located close to each other. (D-F) The Jaccard index matrices J showing overlaps between the neighbors for every pair of neurons Ni, Nj when the network neighborhood defined is that of (D) pre-synaptic partners, (E) post-synaptic partners and (F) gap-junctional partners. The large overlap between neighbors of bilaterally symmetric neurons is indicated by the occurrence of bands of brightly colored entries along the diagonal (note that bilaterally symmetric neurons are always located on adjacent rows/columns). (TIF) [file pcbi.1007602.s012.tif]

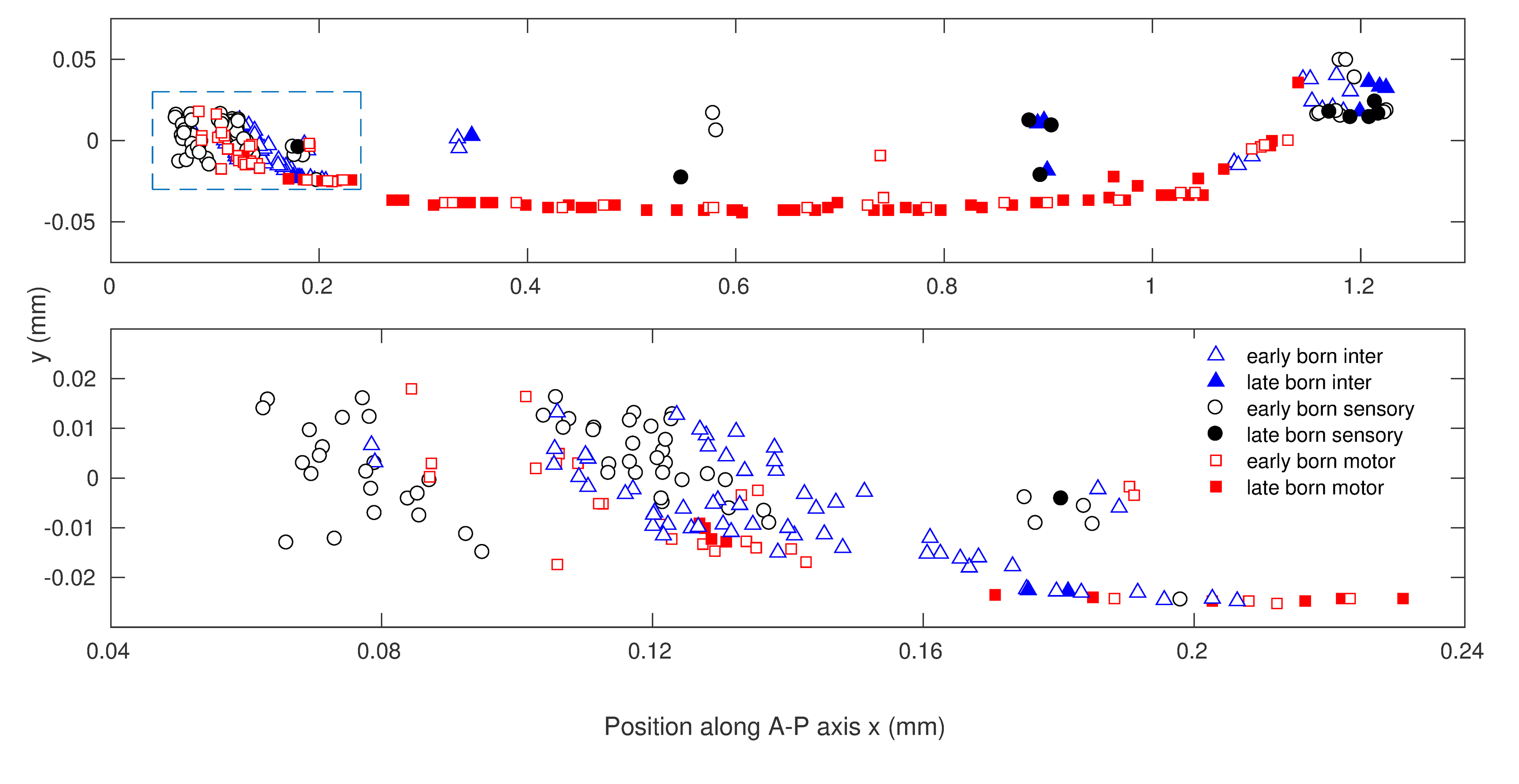

Supplement: S13 Fig — Projections of the physical locations of the neuronal cell bodies, distinguished according to functional type (sensory: circles, inter: triangles and motor: squares) and whether they appear in the early (unfilled symbols) or late (filled symbols) developmental epochs, on the two-dimensional plane formed by the anterior-posterior (AP) axis (x, along the horizontal) and the dorsal-ventral axis (y, along the vertical). Top panel shows the entire worm, with its body oriented such that the head is located near the left end and tail near the right end of the plane. The bottom panel shows a magnified view of the region near the head (bounded by broken lines in the top panel). We note that almost all cells in this region appear at the embryonic stage, during the early burst of development. By contrast, the ventral cord predominantly comprises neurons that appear in the post-embryonic stage (see top panel). (TIF) [file pcbi.1007602.s013.tif]

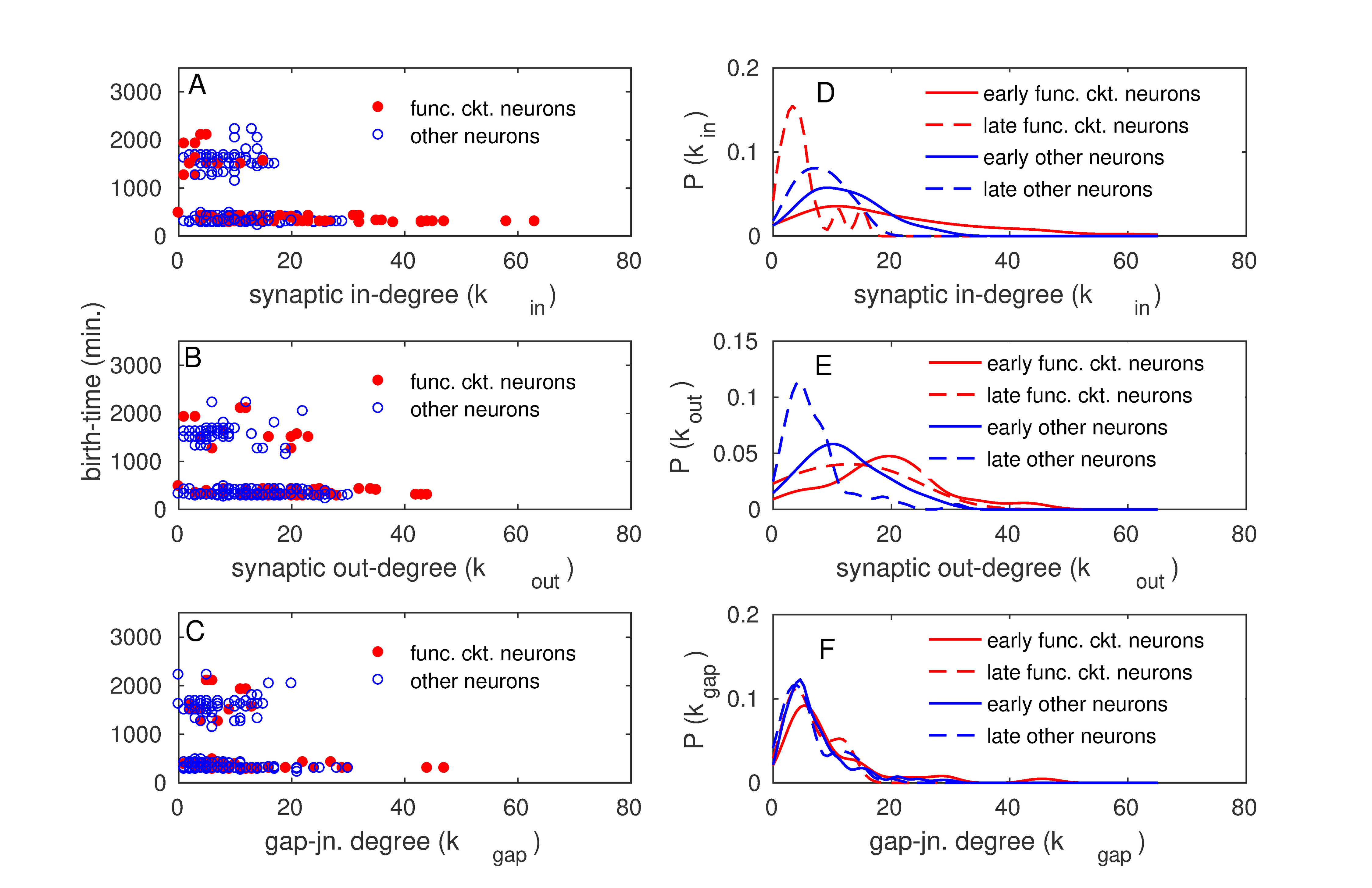

Supplement: S14 Fig — (A-C) Scatter plots indicating the relation between the time of appearance of a neuron and its number of (A) incoming synaptic connections from other cells (synaptic in-degree), (B) outgoing synaptic connections to other cells (synaptic out-degree) and (C) gap junctions with other cells (gap-junctional degree). Filled circles represent neurons belonging to any of nine previously identified functional circuits (see Fig 8 in main text) while unfilled circles show other neurons. (D-F) Probability distributions of different types of connections for neurons categorized in terms of those which are functionally critical, i.e., belong to a functional circuit (red), or not (blue), and whether they appear in the early (solid curve) or late (broken curve) developmental epochs. The different panels correspond to (D) synaptic in-degree, (E) synaptic out-degree and (F) gap-junctional degree. Synaptic in-degree for functionally critical, early-born neurons is seen to have a heavy-tailed distribution which is significantly different from that of the other types of neurons (in terms of a 2-sample Kolmogorov-Smirnov test at 5% level of significance). For the case of synaptic out-degree, however, the distributions for functionally critical neurons that are born at different epochs are statistically indistinguishable. However, for other neurons, the distribution of those that are born in the later, post-embryonic developmental burst are distinct from those that are born early (demonstrated by a 2-sample Kolmogorov-Smirnov test at 5% level of significance). This statistically significant difference between the outgoing connections of early and late-born neurons could arise from the former neurons being present for a much longer period during which they can send out synapses. Distributions of gap junctional connections for all categories of neuron appear to be statistically indistinguishable, suggesting that gap junction formation is relatively unaffected by the functional criticality or [file pcbi.1007602.s014.tif]

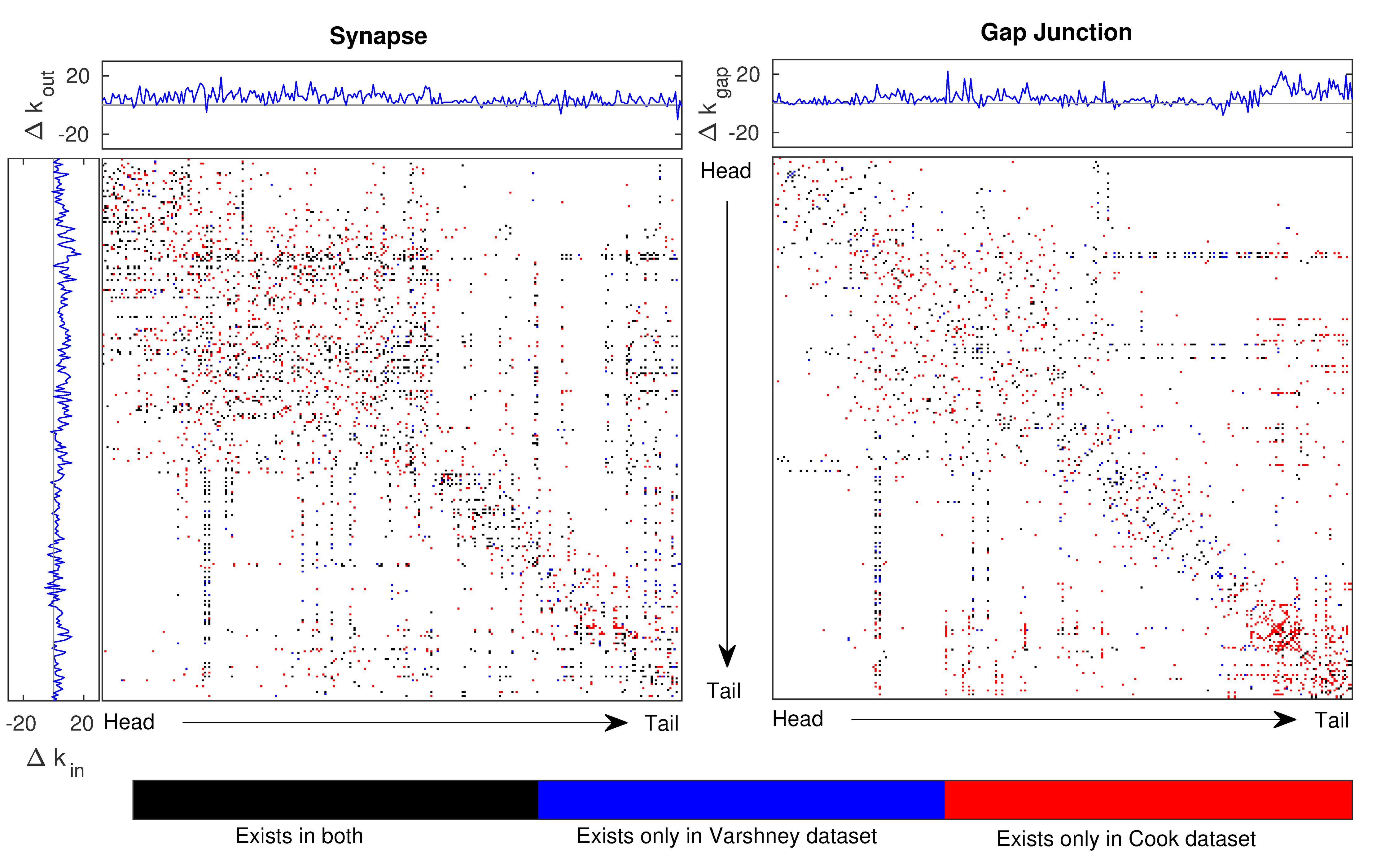

Supplement: S15 Fig — Difference between the adjacency matrices corresponding to the two connectivity data-sets are shown for synapses (left) and gap-junctions (right). Black-colored entries represent connections common to both datasets, while red- and blue-colored entries denote connections that are found exclusively in the Cook and Varshney datasets, respectively. The columns and rows are arranged according to the spatial ordering of the neuronal cell bodies along the head-tail axis. The horizontal and vertical panels for the synapse matrix shows the difference in the out-degree and in-degree, respectively, of each neuron across the two data-sets. For the gap-junction matrix, the horizontal panel shows the change in degree for each neuron across the data-sets. We note that the two data-sets differ substantially, especially in terms of a large number of additional gap-junctions near the tail region that appear in the database of Cook et al. (TIF) [file pcbi.1007602.s015.tif]

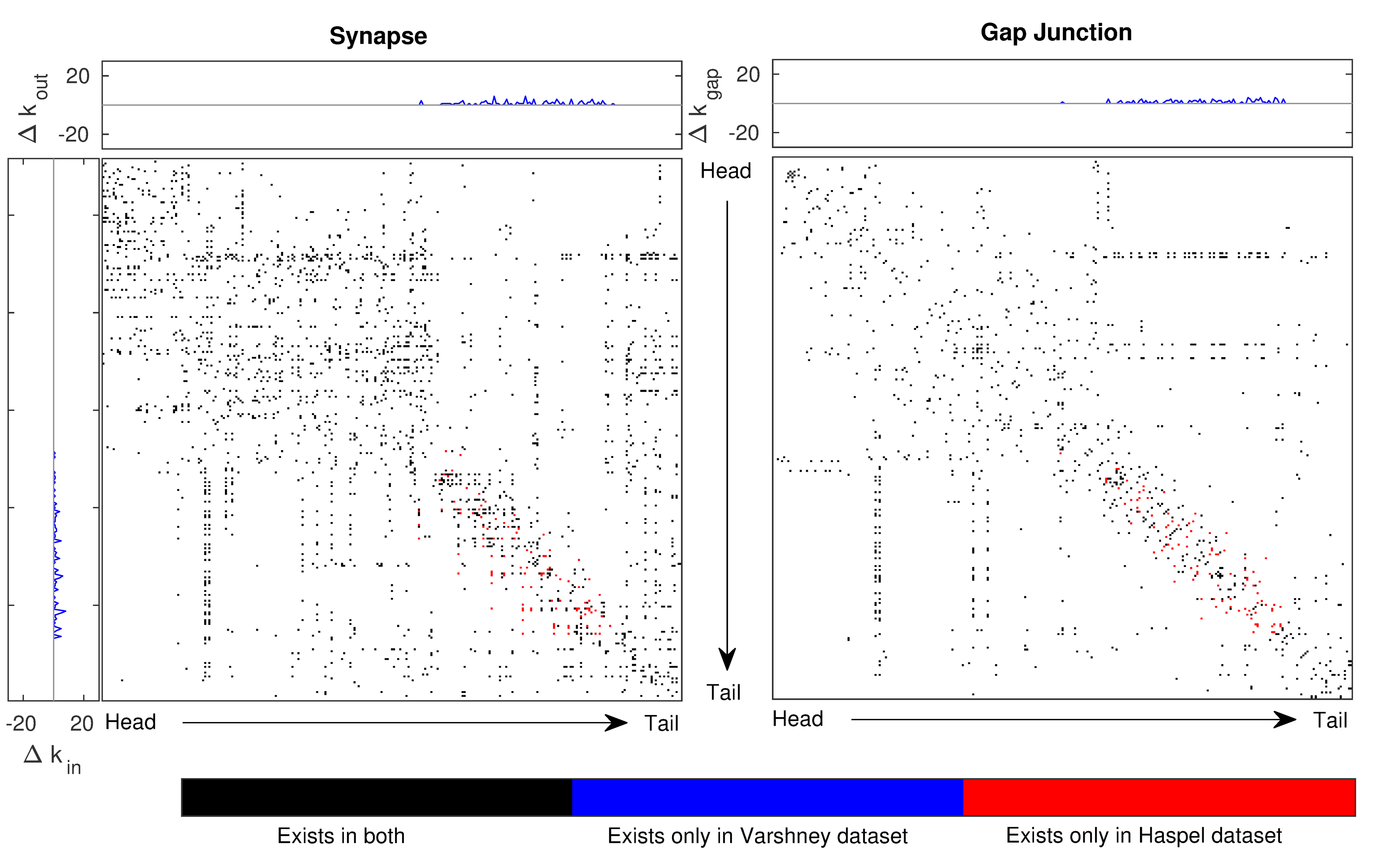

Supplement: S16 Fig — Difference between the adjacency matrices corresponding to the two connectivity data-sets are shown for synapses (left) and gap-junctions (right). Black-colored entries represent connections common to both datasets, while red- and blue-colored entries denote connections that are found exclusively in the Haspel and Varshney datasets, respectively. The columns and rows are arranged according to the spatial ordering of the neuronal cell bodies along the head-tail axis. The horizontal and vertical panels for the synapse matrix shows the difference in the out-degree and in-degree, respectively, of each neuron across the two data-sets. For the gap-junction matrix, the horizontal panel shows the change in degree for each neuron across the data-sets. We note that the two data-sets differ relatively little. (TIF) [file pcbi.1007602.s016.tif]
